# Sustained improvements in human–cat interaction in the shelter sector: a study of a best-practice educational intervention

**DOI:** 10.3389/fvets.2026.1880180

**Published:** 2026-08-10

**Authors:** Elle V. Hatam, Ana M. Barcelos, James Waterman, Avni Bhatia, Lauren Finka

**Affiliations:** 1 Cats Protection, Haywards Heath, United Kingdom; 2 Department of Social Life Sciences – Landbased, Wrexham Glyndwr University, Wrexham, United Kingdom; 3 RedPony Analytics, Llanberis, United Kingdom

**Keywords:** animal handling, animal welfare, behaviour change, best practice guidelines, cat behaviour, shelter cats

## Abstract

Human-cat interaction (HCI) in rescue settings is often inconsistent, driven by individual caregiver beliefs rather than standardised evidence-based practice. This study evaluates the efficacy of an educational intervention for shelter workers, incorporating evidence based HCI principles designed to promote cat comfort and reduce the risk of cat human-directed aggression. A pre-post study followed employees and volunteers across three timepoints: a pre-intervention baseline (T1, *n* = 47), 2 weeks post-intervention (T2, *n* = 47), and a six-month follow-up (T3, *n* = 19). To assess intervention efficacy, participants’ real-time HCI were filmed at each time point and blind-rated by cat behaviour experts based on the degree to which participants’ cat directed behaviour aligned with evidence based best practice HCI principles (either ‘closely’, ‘somewhat’ or ‘not at all’ aligned). Participants also completed self-reported attitude and practice questionnaires that were similarly designed to assess alignment with best practice HCI principles and approaches. Composite ‘attitude’ and ‘practice’ scores were generated with higher scores reflecting greater alignment with best practice HCI principles. Cumulative Link Mixed Models (CLMM) revealed very strong evidence that participants real-time HCI ratings improved post-intervention [LR*χ*^2^(2) = 37.07, *p* < 0.001], with these gains remaining stable at the six-month follow-up. Specifically, the probability of receiving a “closely aligned” HCI rating increased from 2% (±3%) at T1 to 25% (±16%) at T2 (*p* = 0.008), rising further to 37% (±27%) at T3 (*p* = 0.023). At baseline prior to training, higher participant attitude scores were significantly predictive of higher real-time HCI ratings. This association disappeared post-intervention as both HCI ratings and attitude scores increased across the population, effectively reducing score variance and suggesting the training “levelled the playing field” across participants regardless of their starting beliefs. Staff with shorter service lengths (under 2 years) were nearly twice as likely to achieve higher HCI ratings post intervention compared to longer serving staff members, suggesting training may be more effective when delivered within shorter post-recruitment windows. The educational intervention was effective in promoting positive and sustainable improvements in participant’s HCI, highlighting the benefits of evidence-based training delivered to shelter workers.

## 1 Introduction

Approximately 150,000 cats enter rescue settings across the UK annually (53) and recent investigations suggest substantial variability in how cats are managed within the sector (1–4). In these settings, cats often struggle to meet their species-specific needs and express natural behaviours, largely due to the close proximity of other cats, unfamiliar caregivers, and a lack of control over their environment and the routines to which they are exposed (54). There are various interventions that can reduce stress and promote opportunities for cats to experience positive emotions in shelters. These include housing cats singly or in appropriate social groups based on behavioural compatibility (5), ensuring that environmental resources are available in appropriate quantities to the number of cats housed, providing adequate opportunities for safety and concealment (6, 48), and providing enrichment items that encourage play and exploration (48), and that stimulate the cats’ predatory sequence (7, 55). Social interactions with humans may also be beneficial for shelter cats, but only when the human is perceived as a positive stimulus (8). Human-cat interactions are more likely to be perceived positively when they are cat-centred, allowing the cat choice and control over the interaction, respecting individual preferences, and responding appropriately to behavioural signs of comfort or discomfort (9, 10). Animal-centred approaches that prioritise their consent, agency, and comfort during human-animal interactions (HAI) are gaining traction (11, 12), although there is typically a lag between current scientific thinking and its practical implementation. Cats’ capacity to cope well with and benefit from human interaction is likely influenced by their degree of previous human-socialisation (13), individual differences in temperament or personality that shape behavioural responses across time and contexts (14), individual preferences (15, 16), and the humans’ characteristics and behavioural styles (17, 18). High reported rates of human-directed aggression during human-cat interactions (HCI) with socialised cats (56), and humans’ difficulties in effectively inferring cats’ emotional and motivational states (19–21), may suggest a frequent misalignment between the cats’ preferences and humans’ HCI styles. These interaction styles are often governed by individual intuition rather than standardised professional protocols. Direct attempts to increase cats’ tolerance to handling via operant conditioning may help to reduce human-directed aggression under certain conditions (22), however, cat-centred approaches that respect cats’ innate social preferences and instead focus on human rather than cat behaviour change during HCI are also effective (9).

Beyond meeting statutory obligations, there are strong practical and ethical reasons to ensure high-quality education, training, supervision, and continuing professional development (CPD) for animal care professionals. Numerous studies demonstrate that the behaviour and welfare of animals are shaped by the nature of their interactions with caregivers. Structured training programmes targeting caregiver attitudes, beliefs, and behaviours have been shown to positively impact on animal welfare, while also enhancing caregiver job satisfaction and reducing turnover. In addition, given the continual evolution and understanding of optimal approaches to animal care, CPD is essential for maintaining and improving professional competency within animal care roles (59). However, the efficacy of educational programmes may be challenged by entrenched routines, as the ‘unlearning’ of established habits is often as critical to welfare as the acquisition of new skills (23, 24, 55). A potential strategy to address such resistance is the robust evaluation of training efficacy, which ensures that training interventions directly translate into practical animal welfare benefits (25).

A set of ‘best-practice’ human-cat interaction guidelines were previously developed and their positive impact on human-cat and cat-human directed behaviour assessed within a shelter context (9). Using a “CAT” acronym, the guidelines aimed to provide a memorable and simple-to-implement set of instructions to follow during HCI contexts outside of formal veterinary handling. The “C” represents providing the cats with choice and control during the HCI, affording them greater agency by enabling them to both “opt in” and “opt out.” The “A” encourages people to pay attention to the cats’ behavioural and postural responses during interactions, and to moderate their behaviour accordingly. The “T” encourages people to restrict their touching primarily to the cats’ temporal regions. When tested with members of the public, application of the CAT guidelines encouraged more cat-centric approaches to humans’ cat-directed behaviour, leading to real-time improvements in cats’ human-directed behaviours and degree of comfort (9).

In related studies in shelters (26) human individual differences were associated with important variations in their baseline HCI styles as well as the degree to which participants adopted the CAT guidelines, following a brief training intervention. Such findings highlight the importance of considering human individual differences and contextual factors and their potential role in supporting the efficacy of training interventions.

While the CAT guidelines proved to be a useful tool in enabling positive short-term changes to humans’ HCI under supervised conditions, their value as an intervention to support sustainable long-term benefits when adopted by staff within a shelter context remains untested. To this effect, an educational intervention incorporating the guidelines and its underpinning approach was developed and delivered to a group of Cats Protection (CP) employees and volunteers. The intervention was based on the CAT guidelines, which are grounded in cat-centred ethological principles prioritising cat autonomy, comfort and individual preferences (9, 10, 27).

The primary aim of this study was to determine whether the educational intervention was associated with positive and temporally stable (at 2 weeks and 6 months post-intervention) shifts in real-life HCI behaviours among shelter workers and in their self-reported practices and attitudes toward HCI.

The secondary aim was to explore whether participant characteristics, such as length of employment, age, and/or their baseline self-reported attitude and practices toward HCI, were associated with their real-life cat HCI behaviours pre- and post-intervention.

Details of the research questions are available in the subsection 2.3 in the Material and Methods.

## 2 Materials and methods

### 2.1 Research ethics

Ethical approval for this study was granted by Wrexham University Ethics Committee (approval number 2024-627). All participants provided written informed consent prior to participation. Ethical review and approval were not required for the animal component of the study because no experimental procedures were carried out. The study involved observational recordings of naturally occurring interactions between participants and either their own pet cat or a cat in Cats Protection care during routine husbandry or social interactions, and no interventions were applied beyond normal handling. Participants were instructed to prioritise the welfare of both themselves and the cat throughout data collection and were free to stop the interaction or recording at any time if they considered it appropriate to do so.

### 2.2 Overview of the study design

**Figure 1:**
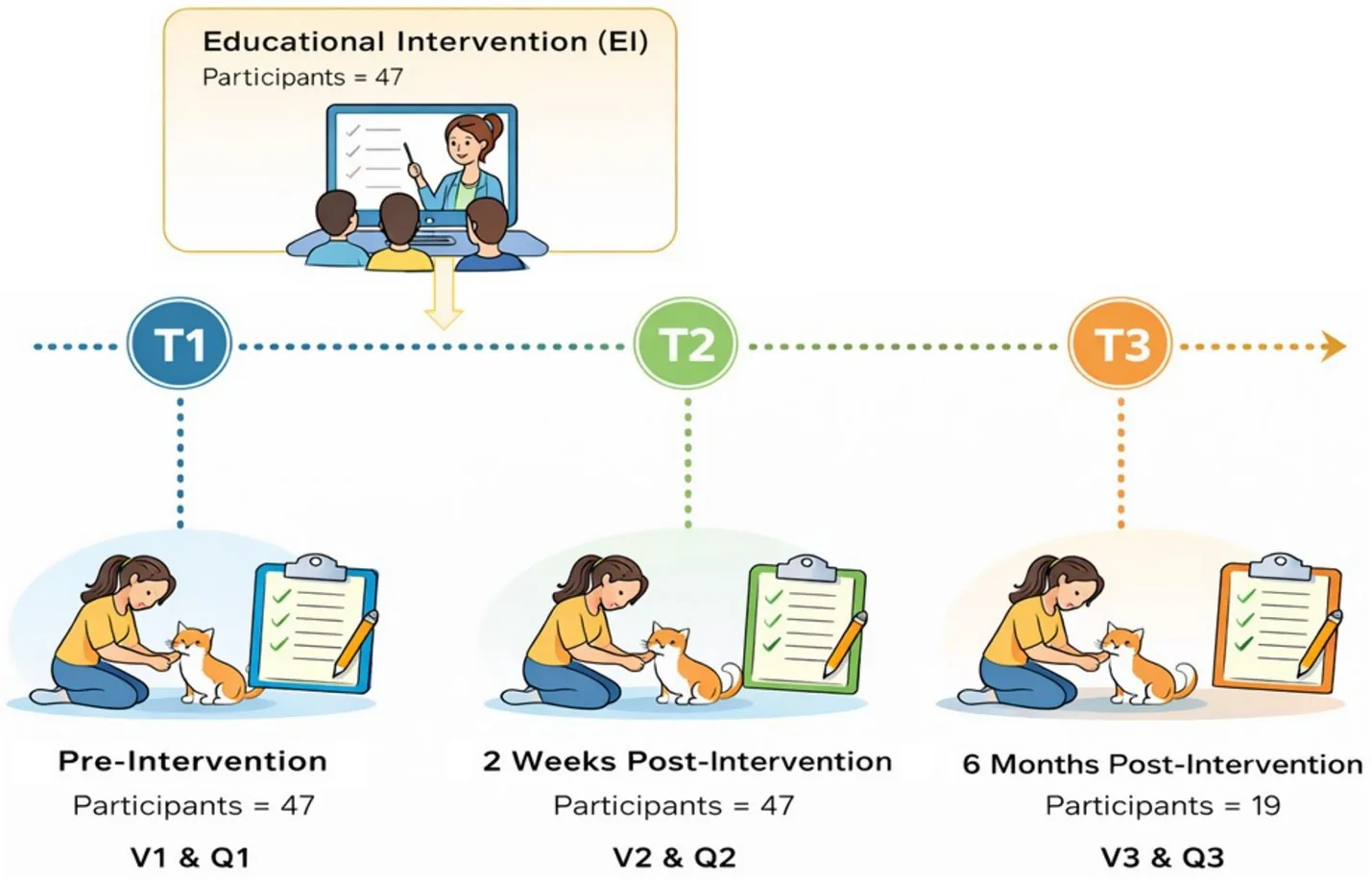
Visual summary of the intervention schedule, data collection points and associated sample sizes. Timepoints T1 (pre-intervention), T2 (2-week post-intervention), and T3 (6-month follow-up) included a 90-s video (V1–V3) of participant-cat interaction and a questionnaire (Q1–Q3). The educational intervention was delivered between T1 and T2.

The study followed participants across three timepoints (T1, T2, T3). At baseline (T1), prior to any training, participants submitted a short, 90 s video of themselves interacting with a cat and answered a questionnaire (details below) about self-reported practices and attitudes around HCI. The intervention was then delivered, with repeated video and questionnaire sampling occurring at 2 weeks (T2) and 6 months (T3) post-intervention. The use of both questionnaires and videos allowed us to track both self-reported perspectives around HCI and observable (real-life) HCI behaviour over time, and to test whether individual human differences at baseline were associated with variations in participant’s real-life HCI. A visual summary of the study design is provided in Figure 1, with key terms and acronyms defined in the accompanying glossary (Table 1).

**Table 1:** Glossary of terms and acronyms used to simplify the study methodology.

Term	Acronym	Description
Behaviour Alignment		A 3-point scale (1 = not at all aligned, 2 = somewhat aligned, 3 = closely aligned) used to rate videos based on how closely aligned with the CAT handling guidelines the participant’s behaviour was at each time point. Ratings were assigned by two blinded cat behaviour and welfare researchers.
Timepoint 1	T1	Pre-intervention baseline data collection.
Timepoint 2	T2	Post-intervention data collection occurring approximately 2 weeks after the educational intervention
Timepoint 3	T3	Post-intervention data collection occurring approximately 6 months after the educational intervention
Questionnaire 1	Q1	A survey assessing participant attitudes and practices regarding HCI, including several demographic questions (e.g., age, work role, prior training). Part of baseline data collection administered at T1; prior to the educational intervention.
Questionnaire 2	Q2	A repeat of Q1 administered to participants at T2; approximately 2 weeks after the educational intervention
Questionnaire 3	Q3	A repeat of Q1 administered to participants at T3; approximately 6 months after the educational intervention
Video 1	V1	Pre-intervention 90-s video of participant interacting with a cat; part of baseline data collection administered at T1; completed prior to Q1 and the educational intervention.
Video 2	V2	Post-intervention video of participant interacting with the same cat as in V1, occurring at T2; approximately 2 weeks after the educational intervention
Video 3	V3	Post-intervention video of participant interacting with the same cat as in V1 and V2, occurring at T3; approximately 6 months after the educational intervention
Attitude domain		Questionnaire section assessing participants’ beliefs and perspectives about human-cat relationships and interaction principles.
Practice domain		Questionnaire section assessing participants’ self-reported behavioural approaches and techniques used during human-cat interactions.
Median attitude		Participant’s median response across all attitude domain questions at a given timepoint (T1, T2, or T3), used to represent conceptual alignment toward best-practice HCI approaches, with a higher median score representing greater alignment
Median practice		Participant’s median response across all practice questions at a given timepoint (T1, T2, or T3), used to represent self-reported real-life approaches toward HCI behaviour with a higher median score representing greater alignment with best-practice

### 2.3 Questionnaire development

The authors developed a 45-item online questionnaire (Supplementary File 1), with 15 ‘Attitude’s’ statements and 30 ‘Practice’s’ questions. The purpose was to capture diversity in people’s perceptions about, and practical approaches toward, human-cat interactions (HCI). Specifically, the statements and questions aimed to gauge the degree to which participants respected the diversity in cats’ social spectrums and interaction preferences, as well as their need for autonomy within human-social contexts. Each ‘Attitude’s’ statement had a 5-point Likert agreement scale (1 - strongly disagree, 2 - disagree, 3 - neither disagree nor agree, 4 - agree, 5 - strongly agree). Each ‘Practice’s’ question had a 5 point-Likert scale (1 - Never, 2 - Rarely, 3 - Sometimes, 4 - Often, 5 - Always). Two examples of the statements included in the attitudes section were “Cats coming in to shelters as strays should always be homed as pet cats” and “It is hard to tell when cats are uncomfortable during interactions.” Two examples of the questions included in the practices section were “Do you typically remain a distance from the cat and wait to see if they approach you or not?” and “Do you typically in situations where you are worried about a cat behaving aggressively, use a fake hand?”

In addition to the 15 statements and 30 questions described above, the baseline (T1) questionnaire also requested demographic information from participants, including age category (26–35 years, 36–45 years, 46–55 years, 56–65 years, or 66–75 years), gender, their work status (i.e., whether they were an employee or volunteer), the length of time that they have been with Cats Protection (0–12 months, 1–2 years, 2–3 years, 3–4 years, 4–5 years, 5–10 years, 10–15 years, 15–20 years, or more than 20 years), and whether they had attended a previous course/training on feline interactions at Cats Protection (yes or no); this previous training did not include information on the current CAT guidelines and its application.

### 2.4 Participant recruitment

To take part in this study, participants were required to be over 18 years old and current employees or volunteers at Cats Protection, although not necessarily involved in the direct care of cats. Participants were recruited by a ‘call to action’ advertisement sent via internal communication channels. Employees and volunteers who expressed interest were provided with additional information about what would be required of them and were asked to sign a consent form (Supplementary File 2).

### 2.5 Data collection pre-intervention (T1)—videos and questionnaire

T1: Before taking part in the educational intervention, protocols were given to the participants (Supplementary File 3), and each was asked to record a 90-s video (V1) of themselves interacting with a cat. This was requested prior to delivery of the baseline questionnaire (Q1) to ensure that the questionnaire did not create a bias in their interaction with the cat.

Participants were asked to record a routine interaction with either one of their own pet cats or a cat in Cats Protection care with which they would normally interact during routine husbandry or social care activities as part of their volunteer or employment duties. No interaction protocol was prescribed, and participants were instructed to capture a naturally occurring interaction rather than perform a standardised handling exercise. Consequently, the videos reflected participants’ usual approaches to human-cat interaction within their normal home or shelter environments. Participants were asked to record interactions with the same cat at each timepoint (T1, T2, and T3). However, because the study focused on human behaviour change and participants had flexibility to choose the cat, they would interact with, the study did not involve a fixed cohort of shelter cats and detailed demographic information (e.g., age, sex, breed or rescue background) of the cats was not collected. Participants were instructed to prioritise the welfare of both themselves and the cat throughout recording and were free to stop the interaction or recording at any time if they considered it appropriate to do so. Participants submitted Video 1 to the author (EVH) using the messaging service ‘WhatsApp’, after which they were sent the baseline questionnaire (Q1) to complete on Microsoft Forms (Microsoft Corporation, Redmond, WA, USA). All data collected before and after the educational intervention were anonymised prior to analysis to safeguard participant confidentiality and obscure individual identities.

### 2.6 Educational intervention

The educational intervention was a 2-h presentation (Supplementary File 4) including a range of discussion points promoting the importance and benefit of cat-centred approaches to HCI, in addition to videos that demonstrated how to practically apply the CAT guidelines within the shelter setting (9). The intervention emphasised three core principles:

- *Choice*—allowing cats to control whether and how they engage in social interaction by respecting their autonomy and avoiding forced contact.
- *Attention*—encouraging participants to observe the cat’s behavioural and postural responses throughout the interaction and adjust their behaviour accordingly.
- *Touch*—restricting tactile interactions primarily to the temporal regions of the head and encouraging participants to pause after approximately 3 s of contact to allow the cat to indicate whether it wished the interaction to continue.

Each session was limited to a maximum of 15 participants to encourage greater individual involvement and facilitate group discussion. The sessions were held online on Microsoft Teams (Microsoft Corporation, USA) and were designed and delivered by members of the Cat Welfare Learning team. Remote sessions allowed for pragmatic use of charity funds and a wide geographical range, ensuring participants had access to training without time and travel constraints.

### 2.7 Data collection post-intervention (T2 and T3)

T2: Within 2 weeks (T2) of the educational intervention, participants were sent a second questionnaire via email (Q2). Q2 contained the same statements and questions as Q1, but the demographic questions were removed. Participants were also asked to record and send a second 90-s HCI video of themselves interacting with a cat (V2) via the messaging service ‘WhatsApp’.

T3: Approximately 6 months (T3) after the educational intervention, the original (baseline) participants were re-contacted via email with a request for them to repeat the 90-s HCI video (V3) and questionnaire (Q3).

### 2.8 Data preparation and scoring of questionnaires and real-life HCI videos

Prior to formal analysis, several preparatory steps were undertaken to ensure data quality, interpretability, and alignment with the study’s analytical framework. All Likert-scale responses from Q1, Q2, and Q3 were converted to ordered factors (1–5) and recoded where needed to ensure a higher score always reflected greater alignment with cat-centred approaches and attitudes toward HCI that respect the cat’s autonomy, comfort and individual preferences (9, 10, 27), facilitating interpretability in downstream analyses and visualization. At this point, four questions were excluded following consensus between the authors due to concerns about item ambiguity and conceptual overlap, which risked undermining the validity and underlying interpretability of attitude and practice composite scores.

In line with Haywood et al. (9), video data were blinded-rated using a 3-point behaviour alignment scale that reflected the degree to which participants’ real-life HCI aligned with the CAT guidelines (9): (1 = not at all aligned, 2 = somewhat aligned, 3 = closely aligned). Twenty videos were randomly selected for inter-rater reliability assessment. These were independently rated by two blinded feline welfare researchers, and agreement was quantified using Intraclass Correlation Coefficients (ICC2). Only behaviour alignment ratings with ICC2 ≥ 0.5 were retained for further analysis, in line with accepted thresholds for moderate reliability (57).

### 2.9 Questionnaire validation

Prior to addressing the aims of the study, we designed a three-stage questionnaire validation process. This was conducted to ensure the reliability and interpretability of the questionnaire (28, 29) and to confirm that the attitude and practice scales functioned as intended and captured meaningful variation in participant responses.

#### 2.9.1 Stage 1: Internal scale behaviour

Internal scale behaviour refers to how participants used the attitude and practice scales over time. We examined whether responses were evenly distributed, whether individuals varied in how they used the scale, how often extreme scores were given, and how response patterns shifted across timepoints for each question. This helped confirm that the scales functioned as intended and could capture meaningful variation. This involved four diagnostic checks: (1) Distribution of Likert scores by domain and timepoint, (2) participant-level response variability, (3) proportion of extreme responses, and (4) question-level mean, variability, and temporal trajectory. See Supplementary materials S1–S5 and T1–T4 for full details.

#### 2.9.2 Stage 2: Cross-domain alignment

We then assessed the relationship between attitude and practice scores to evaluate cross-domain coherence. Specifically, we tested whether participants who achieved higher Likert scores in their ‘Attitude’ items also did so in their ‘Practice’ scores (see Supplementary material S6). This was a construct validation step, examining whether the two domains (attitudes and practices) reflect a shared underlying orientation toward best-practice approaches to HCI. A positive association would not only support the conceptual integrity of the CAT principles but also suggest that participants who internalise best-practice principles cognitively are also more likely to apply them behaviourally.

#### 2.9.3 Stage 3: Long-term vs. short-term participant comparison

To assess whether the participants who returned for the 6-month follow-up at T3 (group 1) were meaningfully different from those who only completed the initial T1 and T2 stages (group 2), we compared the two groups using Q1 data collected at T1. Specifically, we examined median T1 self-reported attitude and practice scores, in addition to relevant demographic information. This helped clarify whether the 6-month follow-up (T3) cohort was broadly similar to the original sample (T1 and T2 samples were the same), and whether T3 results could be interpreted as representative rather than limited to a distinct subgroup effect when trying to establish the longitudinal efficacy of the intervention. Further details are provided in Supplementary material T5.

### 2.10 Data analysis

Prior to modelling, we assessed collinearity among categorical predictors (e.g., age category, employment status) using pairwise chi-square tests and Cramér’s *V*. No problematic associations were identified (Cramér’s *V* < 0.5 across all pairs), indicating that the included variables could be modelled concurrently without risk of multicollinearity. To address model convergence issues and retain these variables in the final models, age category and service length were grouped into broader categories based on sample size and category balance/distribution. The final age groups were 26–35 years, 36–55 years, and 56–75 years; service length was grouped as under 2 years and over 2 years.

All analyses were conducted in R [version 4.5.1; (30)], using packages including *ordinal* (31), *glmmTMB* (32), *emmeans* (33), and *ggeffects* (34). Model diagnostics were performed using simulated residuals via the *DHARMa* package (35), allowing for assessment of fit and detection of potential misspecification. Asymptotic *z*-tests were used for post-hoc comparisons.

For the main analysis, we calculated each participant’s median attitude score and median practice score for each questionnaire (Q1, Q2, and Q3). Medians were chosen over means to respect the ordinal nature of the Likert-scale items and to avoid distortion from skewed or inconsistent responses. This approach reduced the number of parameters, avoided question-level modelling, and allowed us to focus on meaningful belief–behaviour patterns at the participant level.

A range of statistical models were used to address the study aims, as described below:

#### 2.10.1 Determine whether the intervention was associated with sustained improvements in staff’s interactions with cats

To evaluate the potential impact of the educational intervention on real-life HCI, we fitted a cumulative link mixed model (CLMM) with a logit link function. This approach is appropriate for ordinal outcomes such as the behaviour alignment ratings (1 = not at all aligned, 2 = somewhat aligned, 3 = closely aligned). The model included a random intercept for participant ID to account for repeated measures, and a fixed effect of timepoint (T1, T2, T3) to test whether behaviour alignment ratings improved post intervention. The data could not support the inclusion of random slopes for timepoint, so these were removed.

#### 2.10.2 Determine whether the intervention was associated with sustained improvements in staff’s conceptual approaches to HCI

To evaluate whether self-reported practices and attitudes improved following the intervention, we specified two generalised linear mixed models (GLMM), each with a different response variable: one modelling variation in practice scores and the other modelling variation in attitude scores. The models included a fixed effect of timepoint (T1, T2, T3) and a random intercept for participant ID. Median attitude scores and median practice scores were treated as continuous outcomes with a Gaussian error distribution, consistent with their approximately symmetric distribution and conceptual interpretation as a composite score.

#### 2.10.3 Determine whether participant characteristics were associated with their real-life interactions with cats at T1 (pre-intervention/baseline)

To explore the association between participant characteristics (e.g., age, work role, prior training) on behaviour alignment ratings before the intervention (T1), we specified a cumulative link model (CLM). The response variable was behaviour alignment rating, and the model used a logit link function. Fixed effects included median attitude scores at T1, median practice scores at T1, age category, work status, service length, and previous training. The model was restricted to data from the baseline questionnaire to isolate associations prior to any influence of the intervention.

#### 2.10.4 Determine whether participant characteristics were associated with their real-life interactions with cats at T2 (2 weeks post-intervention)

To explore whether baseline participant characteristics (e.g., age, work role, prior training) influenced post-intervention behaviour alignment ratings, we specified another CLM. The response variable was behaviour alignment rating at T2 (2 weeks post-intervention). The fixed effects were the same as for subsection 2.6.1: median attitude scores at T1, median practice score at T1, age category, work status, service length, and previous training. This allowed us to identify potential moderators of the intervention’s impact.

## 3 Results

### 3.1 Demographics

A total of 47 participants (original sample) completed T1, the educational intervention, and T2 (2 weeks post-intervention), with 19 subsequently completing T3 (6 months post-intervention). There was no statistical difference in demographics and baseline questionnaire scores (attitude and practice domains) between the original sample and those who completed T3 [see Supplementary material text 5].

Around half of the original sample were employed at Cats Protection (53.2%, *n* = 25) and the other half (46.8%, *n* = 22) were volunteers. Length of service at Cats Protection ranged from 0 to 12 months to over 20 years, with the most common category being 0 to 12 months (29.8%, *n* = 14). Participants’ ages ranged from 26–35 to 66–75 years, with the most common age group being 36–45 years old (34.0%, *n* = 16). The majority of participants were female (93.6%, *n* = 44), 4.3% (*n* = 2) were male and 2.1% (*n* = 1) preferred not to report their gender. Finally, 23.4% of participants (*n* = 11) reported having previously attended a Cats Protection training session on cat handling.

### 3.2 Questionnaire validation

Prior to analysis, the questionnaire underwent a structured three-stage validation process to ensure reliability and appropriate scale functioning (see Section 2.9). Full details of the validation procedures and results are provided in Supplementary Figures S1–S6 and text T1–T5.

#### 3.2.1 Internal scale behaviour

Descriptive diagnostics indicated that both attitude and practice domains elicited meaningful variation across participants and timepoints. Likert responses spanned the full range of available options, with moderate within-participant variability across T1, T2, and T3 suggesting consistent engagement. To assess whether participants were overusing the endpoints of the Likert scale (i.e., “Strongly Agree” or “Strongly Disagree”), we calculated the proportion of responses falling at each extreme for every question across timepoints. We defined a saturation threshold of 50%, meaning that if more than half of responses for a given item clustered at one endpoint, it could indicate limited discriminative value, response bias, or reduced engagement. No item exceeded this threshold. Most items showed a balanced distribution, with endpoint usage typically ranging from 0 to 33% and varied across participants and timepoints. This confirmed that the scales retained their ability to capture meaningful variation and that extreme responses were present but not dominant. Question level summaries showed that some responses remained stable across timepoints, while others shifted following the intervention. Both attitude and practice domains contained questions that demonstrated change, with several practice questions in particular showing stronger agreement postintervention. These patterns indicate that the scales were both interpretable and responsive to expected shifts. These patterns supported the interpretability and responsiveness of the scales. Both domains demonstrated acceptable internal consistency: attitudes (12 items; *α* = 0.68) and practices (29 items; *α* = 0.88). Full diagnostic plots are available in Supplementary Figures S1 through S6.

#### 3.2.2 Cross-domain alignment

Median attitude and practice scores were positively correlated across timepoints, indicating coherent cross-domain alignment (ADD file). Participants with higher median attitude scores were more likely to have higher median practices scores, supporting the underlying construct validity of the questionnaire as a means to assess participants’ variations best-practice HCI approaches and general principles (9, 10, 27).

While attitude and practice scores were positively correlated across all timepoints, the strength and consistency of this relationship varied (ADD file info). At T1, there was substantial variability in reported practices among participants with similar attitude scores. At T2 (i.e., post-intervention), there was tighter coupling between attitudes and practices and suggesting early internalisation of the intervention’s principles. At T3 the relationship between attitudes and practices remained positive but while many participants retained alignment in their attitudes and practices, others diverged, potentially due to contextual constraints, fading intervention effects, or differential reinforcement over time.

### 3.3 Was the intervention associated with sustained improvements in participants’ interactions with cats?

**Figure 2:**
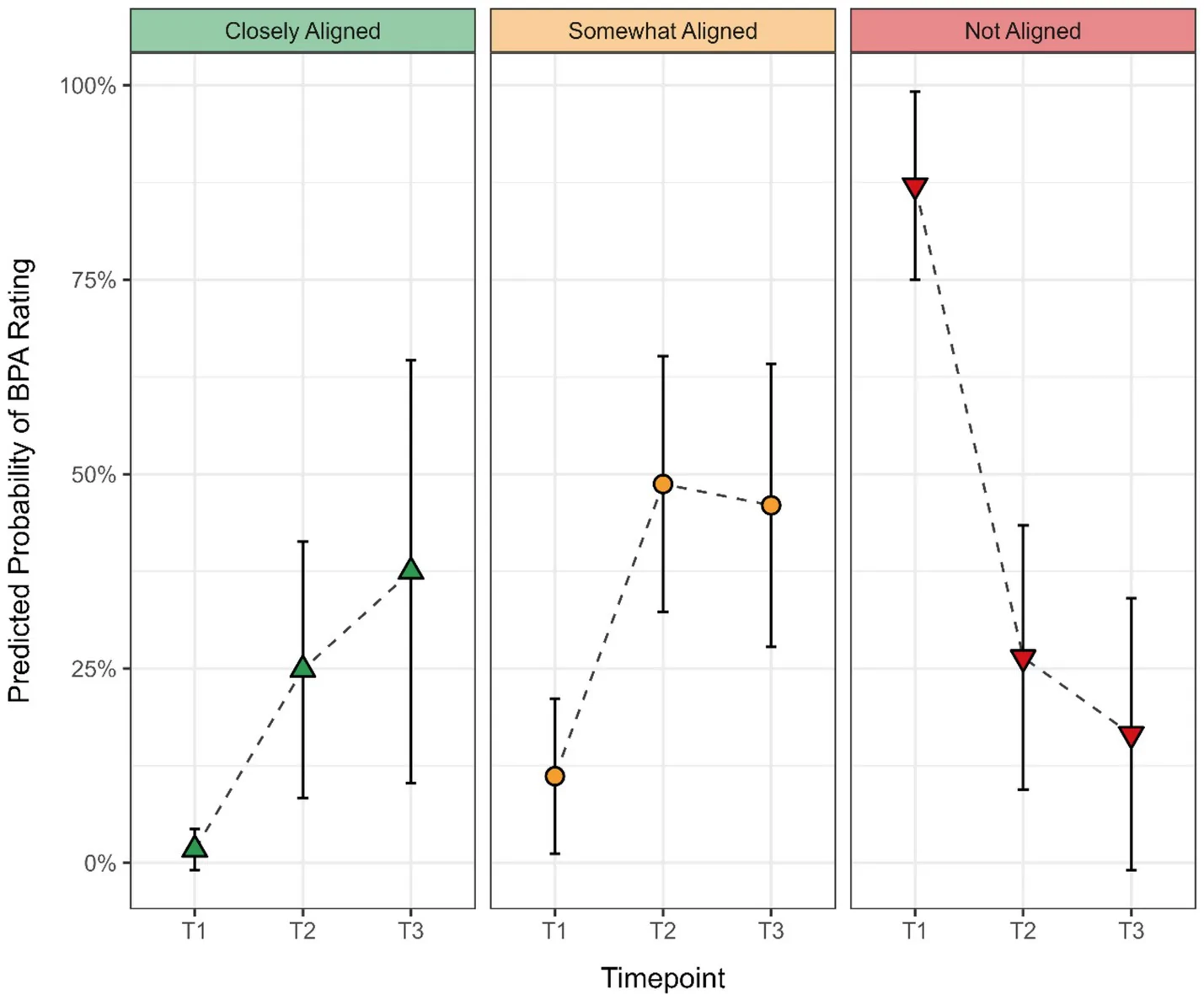
Predicted probabilities of behaviour alignment ratings across the educational intervention timeline. Each panel shows predicted probabilities for one category: “Closely Aligned,” “Somewhat Aligned,” or “Not Aligned.” Points (triangles and circles) represent estimates at each timepoint, with vertical error bars showing uncertainty (95% confidence intervals). Dashed lines connect estimates across timepoints.

There was very strong evidence that staff’s real-life interactions with cats (behaviour alignment ratings) improved post educational intervention [LR *χ*^2^(2) = 37.074, *p* < 0.001]. As shown in Figure 2, post-hoc Tukey HSD tests (adjusted for multiple comparisons) demonstrated that the primary shift in alignment occurred immediately following the intervention, with no significant decay observed at the 6-month follow-up.

Specifically, the probability of being “closely aligned” with best-practice HCI was 2% (±3%) at T1, increasing to 25% (±16%) at T2 (Tukey’s adjusted *z* = 2.97, *p* = 0.008; strong evidence). This increase was stable at 37% (±27%) at T3 (Tukey’s adjusted *z* = 2.63, *p* = 0.023 vs. T1; moderate evidence).

The probability of being “somewhat aligned” also increased post educational intervention from 11% (±10%) at T1 to 49% (±16%) at T2 (Tukey’s adjusted *z* = 3.64, *p* = 0.0008; very strong evidence), and persisted at 46% (±18%) at T3 (Tukey’s adjusted *z* = 3.05, *p* = 0.006 vs. T1; strong evidence).

In contrast, the probability of being “not aligned” declined sharply post educational intervention from 87% (±12%) at T1 to 26% (±17%) at T2 (Tukey’s adjusted *z* = 6.0, *p* < 0.0001; very strong evidence), and remained low at 17% (±17%) at T3 (Tukey’s adjusted *z* = 6.51, *p* < 0.0001 vs. T1; very strong evidence).

### 3.4 Was the intervention associated with sustained improvements in participants’ conceptual approaches to HCI?

**Figure 3:**
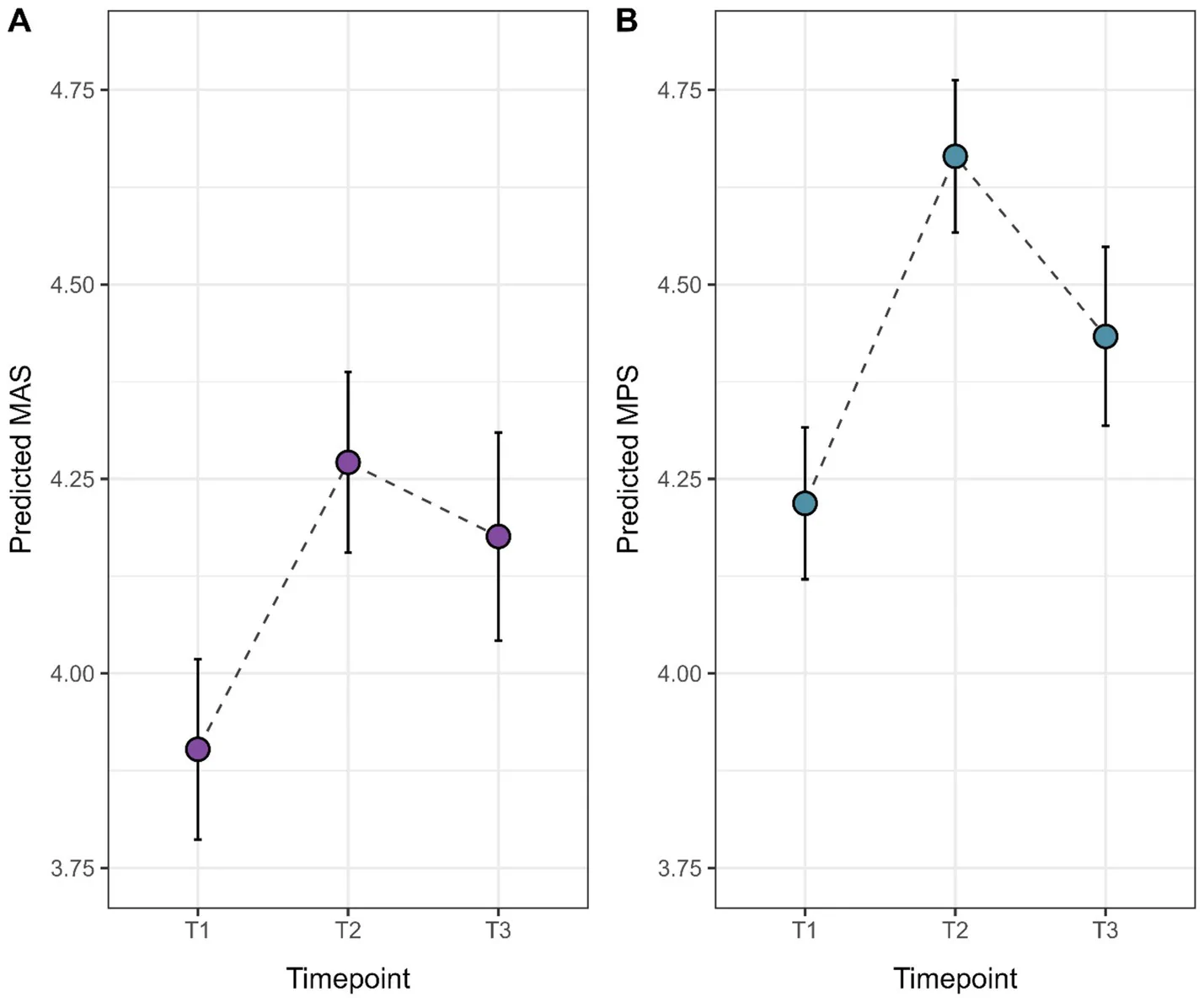
Predicted median attitude scores **(A)** and median practice scores **(B)** across the educational intervention timeline. Points (circles) represent estimates at each timepoint, with vertical error bars showing uncertainty (95% confidence intervals). Dashed lines connect estimates across timepoints.

There was very strong evidence that median attitude scores [LR *χ*^2^(2) = 37.25, *p* < 0.001] and median practice scores [LR *χ*^2^(2) = 55.51, *p* < 0.001] both improved post educational intervention. As shown in Figure 3A, median attitude increased from 3.90 (±0.12) at T1 to 4.27 (±0.12) at T2 (Tukey’s adjusted *t* = 6.75, *p* < 0.0001; very strong evidence). Although scores declined slightly to 4.18 (±0.13) at T3, they remained significantly higher than at T1 (Tukey’s adjusted *t* = 4.25, *p* = 0.0001; very strong evidence).

Median practice scores (Figure 3B) followed the same pattern, increasing from 4.22 (±0.10) at T1 to 4.66 (±0.10) at T2 (Tukey’s adjusted *t* = 9.0, *p* < 0.0001; very strong evidence). Although scores declined to 4.43 (±0.11) at T3, they remained significantly higher than at T1 (Tukey’s adjusted *t* = 3.68, *p* = 0.001; moderate evidence).

### 3.5 Were participant characteristics associated with their interactions with cats at T1 (pre-intervention/baseline)?

**Figure 4:**
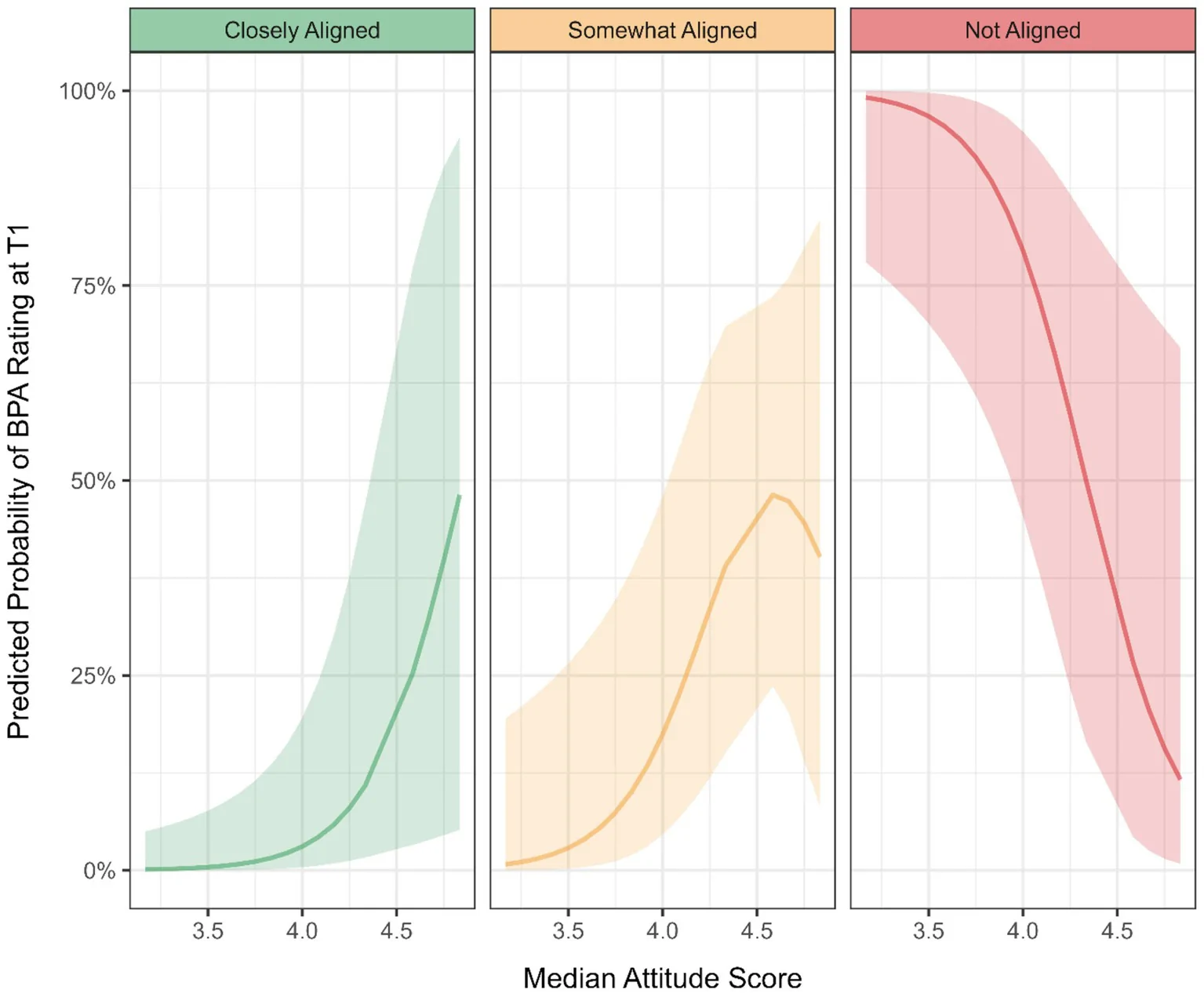
Predicted probabilities of behaviour alignment ratings at T1 across the range of median attitude scores. Estimates are derived from a cumulative link model restricted to pre-intervention T1/Q1 data, with shaded ribbons indicating 95% confidence intervals. Vertical panels represent behaviour alignment rating levels.

There was strong evidence that participants with higher baseline (T1) median attitude scores were more likely to have higher baseline (T1) behaviour alignment ratings [Figure 4; Estimate = 4.06, SE = 1.66, LR *χ*^2^(1) = 7.83, *p* = 0.005]. However, there was little to no evidence of an association between baseline (T1) median practice scores and behaviour alignment (Estimate = −1.95, SE = 1.21, *p* = 0.091).

There was no compelling evidence that any other individual characteristics (e.g., age, work role, service length, prior training) had any influence on baseline (T1) behaviour alignment ratings.

### 3.6 Were participant characteristics associated with their interaction with cats at T2 (2 weeks post-intervention)?

**Figure 5:**
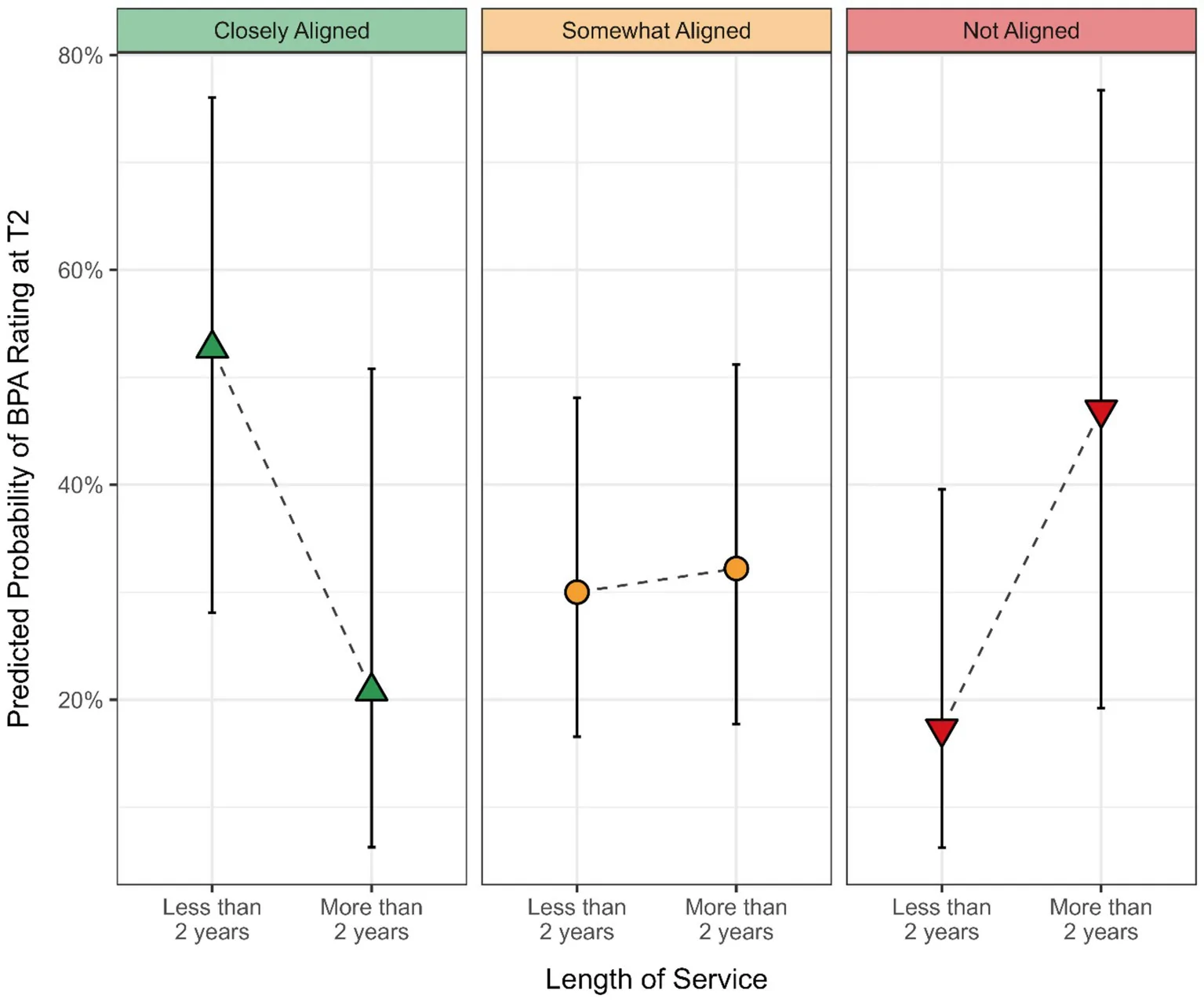
Predicted probabilities of T2 (2 weeks post-intervention) behaviour alignment ratings by length of service. Estimates are derived from cumulative link model using baseline (T1/Q1) participant characteristics. Error bars represent 95% confidence intervals and dashed lines connect estimates within behaviour alignment ratings. Vertical panels represent behaviour alignment rating levels.

Service length was significantly associated with post intervention (T2) behaviour alignment ratings (Estimate = 1.44, SE = 0.71, LR *χ*^2^(1) = 4.47, *p* = 0.034), suggesting the educational intervention had the greatest positive impact on newer staff (those with less than 2 years’ service) compared to those with more than 2 years’ service (Figure 5). No other individual characteristics were associated with post educational intervention (T2) behaviour alignment.

There was no evidence of an association between either baseline (T1) median attitude scores (Estimate = −0.33, SE = 0.88, *p* = 0.703) or baseline (T1) median practice scores (Estimate = 0.86, SE = 0.77, *p* = 0.258) scores and post educational intervention (T2) behaviour alignment ratings.

## 4 Discussion

This study assessed and demonstrated the efficacy of an evidence based educational intervention to support positive and sustainable behaviour change in human-cat interactions (HCI) amongst staff within a shelter setting. The results provide strong evidence that the intervention can facilitate both short-term (2 weeks post-intervention) and long-term (6 months post-intervention) improvements in real-life HCI. Participants demonstrated a substantial shift away from behaviours “not aligned” with best-practice HCI toward “somewhat” and “closely” aligned practices. In addition, their conceptual approaches to HCI (reported data from questionnaires) also shifted positively post-intervention.

Another key finding from this study was that newer staff members (those with less than 2 years’ service) demonstrated the greatest improvements in real-life HCI ratings post intervention, suggesting that the intervention is particularly effective when integrated into early-career professional development. Similarly to Finka et al. (26), participants’ characteristics were also tested in relation to their baseline (pre-intervention) interactions with cats. Crucially, while baseline HCI attitudes (questionnaire data) were associated with baseline real-life HCI, no association was found between baseline HCI attitudes and post-intervention real-life HCI. Therefore, it appears that the intervention “levelled the playing field,” bringing participants into closer behavioural alignment regardless of their starting point.

### 4.1 Sustained improvements in real-life HCI post-intervention

Inconsistent management of cats, including HCI approaches, is a known issue in shelters (1–4). In the present study, substantial improvements in real-life HCI both at 2 weeks and 6 months post-intervention indicate that incorporating the evidence-based CAT guidelines (ref) into educational toolkits can support long-term behavioural change within the shelter sector and, therefore, standardisation in HCI. Potential benefits of such sustained changes span across amelioration in cat and cat carers’ wellbeing, increased cat rehomability, and reduced use of shelter resources toward refresher training.

Furthermore, improvements in the quality of human cat interactions are potentially intrinsically linked to shelter cat rehomability. Cats that are provided with control over their social environment are more likely to engage in the natural, positive behaviours that appeal to potential adopters (8). Thus, the education intervention tested herein could be an additional tool in reducing cat length of stay in shelters. Finally, while CPD is crucial for animal care roles (59), the regularity of specific refresher trainings could be adapted to the duration of its maintenance. Because the educational intervention yielded human behaviour change sustained for 6 months, shelter resources related to refresher training in HCI and/or assessments of HCI quality within a shorter timeframe could be redirected to more urgent needs in shelters.

### 4.2 Improvements in conceptual approaches to HCI post-intervention

A notable finding in this longitudinal study was the divergence between self-reported scores and observable behaviour at the six-month mark. While median attitude and practice scores (self-reported data from the questionnaires) showed a slight decline between 2 weeks follow-up (T2) and 6 months follow-up (T3), participants’ actual behaviour alignment ratings remained stable or continued to improve. This “Say-Do” gap likely indicates a transition from declarative knowledge, the conscious effort to state and follow rules, to procedural habit (58). When participants were “tested” 2 weeks after the educational intervention, the guidelines were likely at the forefront of their conscious thought, leading to peak self-reported scores. By 6 months, these behaviours may have become habituated; integrated into daily routines to the point where participants were less likely to rate their own approach as “exceptional,” even though their real-life performance remained high.

### 4.3 Participants characteristics associated with real-life HCI

Understanding the target audience and the people who need to change is crucial for designing and evaluating human-animal interaction interventions (25). Herein, the intervention efficacy was moderated by the participants’ length of service. Staff/volunteers with less than 2 years’ service were nearly twice as likely to achieve the highest level of alignment 2 weeks post-intervention compared to those with longer service lengths. Interestingly, Finka et al. (26) found that individuals living with cats for longer periods of time showed poorer alignment with best-practice HCI. Together, these findings suggest that individuals with more prolonged exposure to cat care may be less receptive to incorporating new approaches that require altering pre-existing HCI styles. It is possible that the greater degrees of positive behaviour change among people who are “newer” to the shelter sector reflects greater “cognitive pliability” (36) including fewer established habits (37), enabling the adoption of cat-centred protocols as their primary “default” setting (38). Conversely, for those with longer service, the intervention requires not just the acquisition of new skills, but the active unlearning of entrenched routines (39). This finding suggests that educational interventions may be most effective when delivered early in employment, before work routines become well established, whereas staff and volunteers with longer service may benefit from additional behaviour change strategies designed to support modification of existing routines. Therefore, it is recommended that evidence based HCI training is integrated into standard induction processes before suboptimal cat handling and interaction approaches become ingrained. However, where behaviour change is required among more experienced staff, interventions may need to use different strategies, such as those outlined in the Transtheoretical (40) and COM-B (23) models of behaviour change. For example, increasing awareness amongst experienced staff of problematic HCI practices and emphasising that the benefits of change outweigh the perceived costs (40).

Notably, the intervention appeared to standardise HCI behaviours across the cohort, effectively “levelling of the playing field.” At baseline, a significant positive association was observed between participants’ attitudes (questionnaire data). However, 2 weeks post-intervention, HCI quality was no longer associated with baseline attitudes. This suggest that, prior to formal training, HCI quality may have been largely driven by participants’ intrinsic motivation and personal beliefs, whereas post-intervention, it was more strongly guided by a structured and objective framework (i.e., the CAT guidelines).

A shift in shelter cat care away from reliance on intuitive and potentially inconsistent handling and toward more standardised, cat centred and evidence-based approaches, may provide cats with a greater sense of social predictability. Social predictability is a cornerstone of feline welfare, as it can reduce stress associated with inconsistent handling and varying caregivers (41, 42). Conversely, low predictability in care has been linked to stress responses in cats (43).

This shift toward standardised HCI may also help mitigate the risk of blind spots in animal care settings. Even long serving or experienced caregivers can develop “functional fixedness,” where entrenched habits or over-familiarity may lead them to overlook subtle feline stress signals (44). The use of a standardised, evidence-based framework, such as the CAT guidelines, may encourage more “conscious checks” of behaviour, helping to ensure that even those with high baseline experience remain tethered to evidence-based practice (19, 45).

### 4.4 Study limitations and future directions

While the results are encouraging, a few limitations must be acknowledged. First, the study cohort size was modest, particularly at the six-month follow-up (T3), which may limit the generalisability of the long-term findings. Although no statistically significant differences were identified between participants who completed the six-month follow up and the original cohort in terms of demographic characteristics or baseline questionnaire scores, the reduced sample size may nevertheless have introduced the potential for bias by limiting the representation of participants who discontinued the study. Consequently, the sustained improvements observed at T3 should be interpreted with caution until replicated in larger longitudinal studies. Second, participants selected the cats they filmed for assessment, using either their own pet cat or a cat in Cats Protection care with which they would normally interact. Consequently, the study did not involve a fixed cohort of shelter cats, and detailed demographic information (e.g., age, sex, breed or rescue background) was not collected. Variation in cat familiarity or individual temperament may have influenced how they interacted with cats and, therefore, behaviour alignment ratings. Third, there was an overrepresentation of female participants in the study. However, this is a known issue in human-animal interaction studies (26, 46) and therefore may reflect broader sampling biases within this field, potentially limiting the generalisability of the findings. Furthermore, although some evidence suggests that aspects of cats’ behaviour may differ according to caregiver sex, this area remains relatively underexplored (47). Future research should therefore aim to recruit a more balanced representation of male and female participants to determine whether the effectiveness of educational interventions varies by caregiver sex. However, as there was no association between participants’ exposure to another cat handling training pre-intervention and the quality of their interactions with cats at baseline, it is unlikely that other training would have driven the same changes observed herein. Future research should aim to replicate these findings in larger, cross-organisational cohorts to confirm that the intervention remains effective across diverse shelter cultures. Additionally, following participants over longer periods of time (e.g., one, 2 years) post-intervention could help determine the best timeframe for a refresher session, as in this study most participants were still behaving in alignment with best practices 6 months post-intervention.

## 5 Conclusion

This study demonstrates that a brief, evidence-based educational intervention can facilitate sustainable, positive shifts in how humans interact with cats in rescue settings, particularly among newer staff/volunteers. Based on these findings, we recommend that evidence-based cat-centred HCI training, (e.g., the CAT guidelines), be integrated into standard induction protocols for staff and volunteers entering the rescue sector. This proactive approach may help to leverage a “window of receptivity” within early-career professionals, establishing a high-quality “default” HCI style before suboptimal habits can form. However, because this study was conducted within a single organisation in the UK, further research is needed to determine whether these findings are replicated across different organisational, geographical and cultural contexts before the effectiveness of the educational intervention and the broader applicability of the CAT guidelines are generalised. Future research should also investigate whether comparable improvements are observed in multi-species rescue environments, where differences in species composition and management practices may influence intervention effectiveness.

## Data Availability

The raw data supporting the conclusions of this article will be made available by the authors, without undue reservation.

## References

[1] Barcelos AM Waterman J Bhatia A McDonald JL Foreman-Worsley R Roberts C . (2026). Part 3: A sector wide survey of UK/British Isles shelter organisations caring for cats: Caregiver reported approaches to cat socialisation [Manuscript submitted for publication].

[2] Finka L. R. Barcelos A. M. Waterman J. Bhatia A. McDonald J. L. Foreman-Worsley R. . (2026a). Part 1: A sector wide survey of UK/British Isles shelter organisations caring for cats: Caregiver reported approaches to housing, husbandry and general care provision. [Manuscript submitted for publication]. 10.3390/vetsci13060587 PMC13308460 42357785

[3] Finka L. R. Barcelos A. M. Waterman J. Bhatia A. McDonald J. L. Foreman-Worsley R. . (2026b). Part 2: A sector wide survey of UK/British Isles shelter organisations caring for cats: Caregiver reported approaches to assessments, behaviour management and homing decisions. Veterinary Sciences [Manuscript submitted for publication]. 10.3390/vetsci13060590 PMC13307826 42357788

[4] Finka L. R. Barcelos A. M. Waterman J. Bhatia A. McDonald J. L. Foreman-Worsley R. . (2026c). Part 4: A sector wide survey of UK/British Isles shelter organisations caring for cats: Caregiver reported approaches to welfare assessments, management, euthanasia decisions making and perceived barriers to better welfare [Manuscript submitted for publication].

[5] Finka LR Ellis SLH Stavisky J. A critically appraised topic (CAT) to compare the effects of single and multi-CAT housing on physiological and behavioural measures of stress in domestic cats in confined environments. BMC Vet Res. (2014) 10:73. doi: 10.1186/1746-6148-10-73, 24655603 PMC3998042

[6] Van der Leij WJR Selman LDAM Vernooij JCM Vinke CM. The effect of a hiding box on stress levels and body weight in Dutch shelter cats: a randomized controlled trial. PLoS One. (2019) 14:e0223492. doi: 10.1371/journal.pone.0223492, 31609987 PMC6791553

[7] Houser B Vitale KR. Increasing shelter cat welfare through enrichment: a review. Appl Anim Behav Sci. (2022) 248:105585. doi: 10.1016/j.applanim.2022.10558

[8] Gourkow N Fraser D. The effect of housing and handling practices on the welfare, behaviour and selection of domestic cats (*Felis sylvestris catus*) by adopters in an animal shelter. Anim Welf. (2006) 15:371–377. doi: 10.1017/s0962728600030700

[9] Haywood C Ripari L Puzzo J Foreman-Worsley R Finka LR. Providing humans with practical, best practice handling guidelines during human-cat interactions increases cats’ affiliative behaviour and reduces aggression and signs of conflict. Front Vet Sci. (2021) 8. doi: 10.3389/fvets.2021.714143, 34434985 PMC8381768

[10] Rodan I Dowgray N Carney HC Carozza E Ellis SLH Heath S . 2022 AAFP/ISFM cat friendly veterinary interaction guidelines: approach and handling techniques. J Feline Med Surg. (2022) 24:1093–132. doi: 10.1177/1098612X221128760, 36259500 PMC10845437

[11] Fennell DA. Animal-informed consent: sled dog tours as asymmetric agential events. Tour Manag. (2022) 93:104584. doi: 10.1016/j.tourman.2022.104584

[12] Mancini C Nannoni E. Relevance, impartiality, welfare and consent: principles of an animal-centered research ethics. Front Anim Sci. (2022) 3:800186. doi: 10.3389/fanim.2022.800186

[13] McCune S. The impact of paternity and early socialisation on the development of cats’ behaviour to people and novel objects. Appl Anim Behav Sci. (1995) 45:109–24. doi: 10.1016/0168-1591(95)00603-P

[14] Travnik IDC Machado DDS Gonçalves LDS Ceballos MC Sant’Anna AC. Temperament in domestic cats: a review of proximate mechanisms, methods of assessment, its effects on human—cat relationships, and one welfare. Animals (Basel). (2020) 10:1516. doi: 10.3390/ani10091516, 32867072 PMC7552130

[15] Ellis SLH Thompson H Guijarro C Zulch H. The influence of body region, handler familiarity and order of region handled on the domestic cat's response to being stroked. Appl Anim Behav Sci. (2015) 173:60–7. doi: 10.1016/j.applanim.2014.11.002

[16] Soennichsen S Chamove AS. Responses of cats to petting by humans. Anthrozoös. (2002) 15:258–65. doi: 10.2752/089279302786992577

[17] Mertens C. Human–cat interactions in the home setting. Anthrozoös. (1991) 4:214–31. doi: 10.2752/089279391787057062

[18] Wedl M Bauer B Gracey D Grabmayer C Spielauer E Day JEL . Factors influencing the temporal patterns of dyadic behaviours and interactions between domestic cats and their owners. Behav Process. (2011) 86:58–67. doi: 10.1016/j.beproc.2010.09.001, 20837114

[19] Dawson LC Cheal J Niel L Mason G. Humans can identify cats’ affective states from subtle facial expressions. Anim Welf. (2019) 28:519–31. doi: 10.7120/09627286.28.4.519

[20] Mouzon C Sueur C Petit O. Human perception of cats’ communicative cues: human–cat communication goes multimodal. Appl Anim Behav Sci. (2023) 267:105987. doi: 10.1016/j.applanim.2023.105987

[21] Prato-Previde E Cannas S Palestrini C Ingraffia S Battini M Ludovico LA . What’s in a meow? A study on human classification and interpretation of domestic cat vocalizations. Animals. (2020) 10:2390. doi: 10.3390/ani10122390, 33327613 PMC7765146

[22] Fritz JN Fletcher VL Dyer SP. Functional analysis and treatment of aggression exhibited by cats toward humans during petting. J Appl Behav Anal. (2022) 55:169–179. doi: 10.1002/jaba.877, 34449088

[23] Michie S van Stralen MM West R. The behaviour change wheel: a new method for characterising and designing behaviour change interventions. Implement Sci. (2011) 6:42. doi: 10.1186/1748-5908-6-42, 21513547 PMC3096582

[24] Verplanken B Wood W. Interventions to break and create consumer habits. J Public Policy Mark. (2006) 25:90–103. doi: 10.1509/jppm.25.1.90

[25] Glanville C Abraham C Coleman G. Human behaviour change interventions in animal care and interactive settings: a review and framework for design and evaluation. Animals. (2020) 10:2333. doi: 10.3390/ani10122333, 33302506 PMC7764651

[26] Finka LR Ripari L Quinlan L Haywood C Puzzo J Jordan A . Investigation of humans individual differences as predictors of their animal interaction styles, focused on the domestic cat. Sci Rep. (2022) 12:12128. doi: 10.1038/s41598-022-15194-7 35840600 PMC9287547

[27] Finka LR. Conspecific and human sociality in the domestic cat: consideration of proximate mechanisms, human selection and implications for cat welfare. Animals. (2022) 12:298. doi: 10.3390/ani12030298, 35158622 PMC8833732

[28] Kimberlin CL Winterstein AG. Validity and reliability of measurement instruments used in research. Am J Health Syst Pharm. (2008) 65:2276–84. doi: 10.2146/ajhp070364, 19020196

[29] Younas A Porr C. A step-by-step approach to developing scales for survey research. Nurse Res. (2025) 26:14–19. doi: 10.7748/nr.2018.e1585, 30456936

[30] R Core Team. R: A Language and Environment for Statistical Computing. Vienna, Austria: R Foundation for Statistical Computing (2025).

[31] Christensen R. H. B. (2023). Ordinal—Regression Models for Ordinal Data. Available online at: https://CRAN.R-project.org/package=ordinal

[32] Brooks ME Kristensen K Benthem KJ van Magnusson A Berg CW Nielsen A . GlmmTMB balances speed and flexibility among packages for zero-inflated generalized linear mixed modeling. R J. (2017) 9:378. doi: 10.32614/RJ-2017-066

[33] Lenth RV. Emmeans: Estimated Marginal Means, Aka Least-Squares Means. (2025) doi: 10.32614/CRAN.package.emmeans.

[34] Lüdecke D. Ggeffects: tidy data frames of marginal effects from regression models. J Open Source Softw. (2018) 3:772. doi: 10.21105/joss.00772

[35] Hartig F. (2024). DHARMa: Residual Diagnostics for Hierarchical (Multi-Level/Mixed) Regression Models. 10.32614/CRAN.package.DHARMa

[36] Verplanken B Walker I Davis A Jurasek M. Context change and travel mode choice: combining the habit discontinuity and self-activation hypotheses. J Environ Psychol. (2008) 28:121–7. doi: 10.1016/j.jenvp.2007.10.005

[37] Greenwood BN Agarwal R Agarwal R Gopal A. The role of individual and organizational expertise in the adoption of new practices. Organ Sci. (2019) 30:191–213. doi: 10.1287/orsc.2018.1246, 19642375

[38] Thomas GO Poortinga W Sautkina E. Habit discontinuity, self-activation, and the diminishing influence of context change: evidence from the UK understanding society survey. PLoS One. (2016) 11:e0153490. doi: 10.1371/journal.pone.0153490, 27120333 PMC4847906

[39] Lacy AMP Lam KC Bacon CEW. Addressing the habitual practice issue: the role of unlearning in promoting evidence-based practice and lifelong learning. Athl Train Educ J. (2022) 17:174–81. doi: 10.4085/1947-380x-21-050

[40] Prochaska JO DiClemente CC. Stages and processes of self-change of smoking: toward an integrative model of change. J Consult Clin Psychol. (1983) 51:390–5. doi: 10.1037/0022-006X.51.3.390, 6863699

[41] Carlstead K Brown JL Strawn W. Behavioral and physiological correlates of stress in laboratory cats. Appl Anim Behav Sci. (1993) 38:143–58. doi: 10.1016/0168-1591(93)90062-t

[42] Gourkow N. (2001). Factors Affecting the Welfare and Adoption Rate of Cats in an Animal Shelter [PhD Thesis]. Vancouver: University of British Columbia

[43] Stella JL Lord LK Buffington CT. Sickness behaviors in response to unusual external events in healthy cats and cats with feline interstitial cystitis. J Am Vet Med Assoc. (2011) 238:67–73. doi: 10.2460/javma.238.1.67, 21194324 PMC3852887

[44] Whitehead ML Canfield PJ Johnson R O’Brien CR Malik R. Case-based clinical reasoning in feline medicine: 3: use of heuristics and illness scripts. J Feline Med Surg. (2016) 18:418–26. doi: 10.1177/1098612X16643251, 27143043 PMC11132198

[45] Mckenzie B. Veterinary clinical decision-making: cognitive biases, external constraints, and strategies for improvement. J Am Vet Med Assoc. (2014) 244:271–6. doi: 10.2460/javma.244.3.271, 24432957

[46] Herzog H. Women Dominate Research on the human–animal bond. New York City: Psychology Today (2021).

[47] Demirbaş YS Kerman K Atılgan D Ünler M Yildirim T Şimşek S. Greeting vocalizations in domestic cats are more frequent with male caregivers. Ethology. (2026) 132:87–94. doi: 10.1111/eth.70033

[48] Ellis SLH. Environmental enrichment: practical strategies for improving feline welfare. J Feline Med Surg. (2009) 11:901–12. doi: 10.1016/j.jfms.2009.09.011 19857853 PMC11383019

[49] Ellis SLH Rodan I Carney HC Heath S Rochlitz I Shearburn LD . AAFP and ISFM feline environmental needs guidelines. J Feline Med Surg. (2013) 15:219–30. doi: 10.1177/1098612X13477537, 23422366 PMC11383066

[50] Green L Kaler J Liu N Ferguson E. Influencing change: when “best practice” changes and the prototypical good farmer turns bad. Front Vet Sci. (2020) 7:161. doi: 10.3389/fvets.2020.00161, 32296722 PMC7136422

[51] Vetter T. Social (un-) learning and the legitimization of marginalized knowledge: how a new community of practice tries to ‘kick the grain habit’in ruminant livestock farming. J Rural Stud. (2020) 79:11–23. doi: 10.1016/j.jrurstud.2020.08.036

[52] White J Sims R. Improving equine welfare through human habit formation. Animals. (2021) 11:2156. doi: 10.3390/ani11082156, 34438614 PMC8388501

[53] Stavisky J Brennan ML Downes M Dean R. Demographics and economic burden of un-owned cats and dogs in the UK: results of a 2010 census. BMC Vet Res (2012) 8:163. doi: 10.1186/1746-6148-8-163 22974242 PMC3514250

[54] Stella J Croney C Buffington T. Environmental factors that affect the behavior and welfare of domestic cats (Felis silvestris catus) housed in cages. Appl. Anim. Behav. Sci. (2014) 160:94–105. doi: 10.1016/j.applanim.2014.08.006

[55] Buffington CA Westropp JL Chew DJ Bolus RR. Clinical evaluation of multimodal environmental modification (MEMO) in the management of cats with idiopathic cystitis. J Feline Med Surg. (2006) 8:261–8. doi: 10.1016/j.jfms.2006.02.002 16616567 PMC10822542

[56] O’Hanley KA Pearl DL Niel L. Risk factors for aggression in adult cats that were fostered through a shelter program as kittens. Appl. Anim. Behav. Sci. (2021) 236:105251. doi: 10.1016/j.applanim.2021.105251

[57] Koo TK Li MY. A Guideline of Selecting and Reporting Intraclass Correlation Coefficients for Reliability Research. J Chiropr Med. (2016) 15:155–63. doi: 10.1016/j.jcm.2016.02.012 27330520 PMC4913118

[58] Anderson JR Bothell D Byrne MD Douglass S Lebiere C Qin Y. An integrated theory of the mind. Psychol Rev. (2004) 111:1036–60. doi: 10.1037/0033-295X.111.4.1036 15482072

[59] Wieland B Daborn C Debnath N Silva-Fletcher A. ‘Continuing professional development for veterinarians in a changing world’, Revue Scientifique et Technique (Office International des Epizooties). (2021) 40:555–566. doi: 10.20506/rst.40.2.3245 34542094

