# Supplementary material for: Sustained improvements in human–cat interaction in the shelter sector: a study of a best-practice educational intervention
Source: Front Vet Sci. 2026 Aug 10;13:1880180. doi: 10.3389/fvets.2026.1880180 (PMC13500327; doi:10.3389/fvets.2026.1880180)
Supplement: Supplementary file 1 [file Data_Sheet_1.PDF]

Supplementary Materials

The PDF file includes:

Figs. S1 to S6

Text T1 to T4

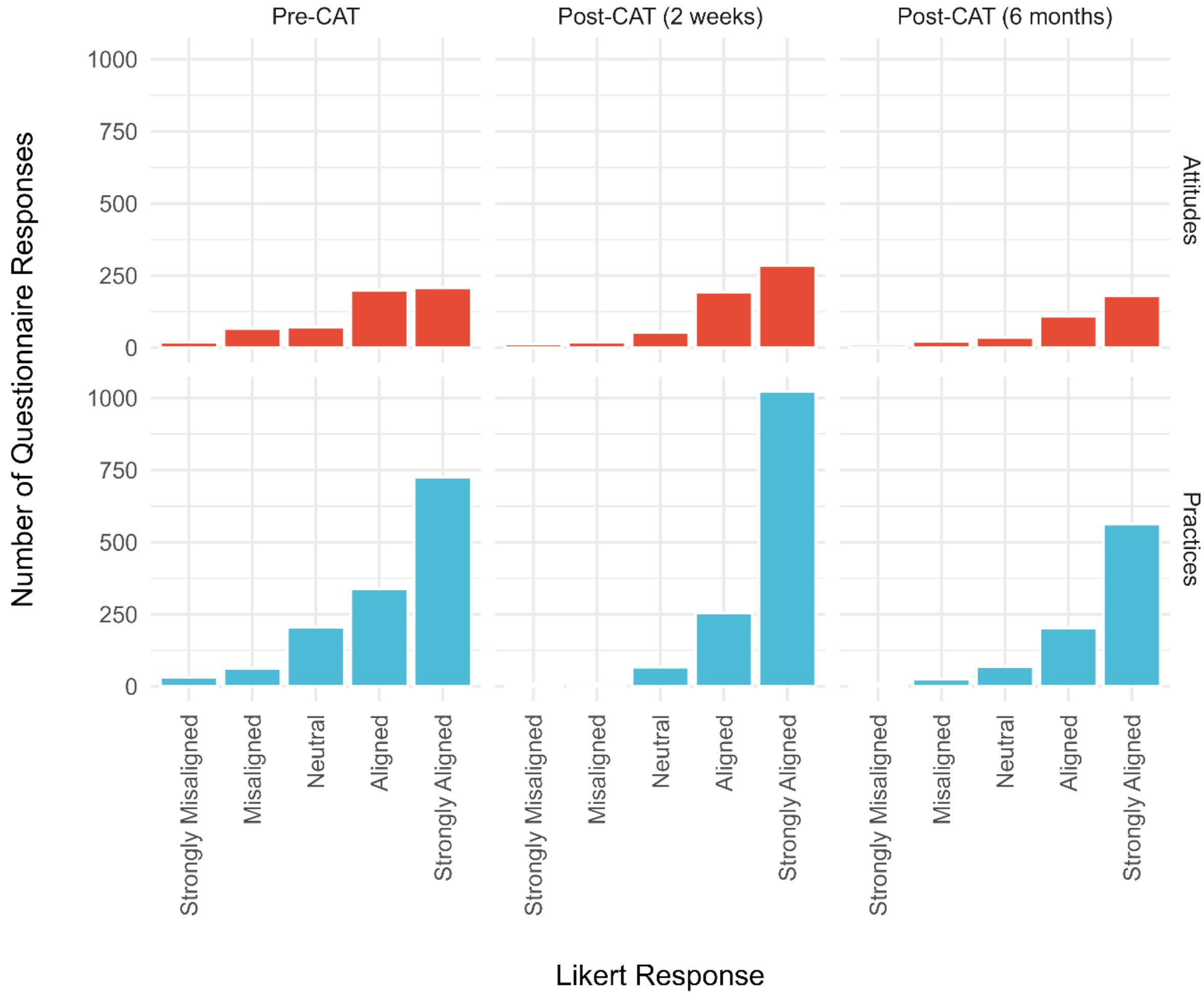

Figure S1. Distribution of Likert (Alignment with Best-Practice) Scores by Question Domain and Condition.

Text T1. Distribution of Likert Scores by Domain and Timepoint

We examined the distribution of Likert responses across attitude and practice questions at three timepoints; Pre-intervention (T1-Q1), 2 weeks post-intervention (T2-Q2) and 6 months post-intervention (T3-Q3). This helped determine whether the full range of response options (i.e., Likert scores from 1 to 5) was being used and whether patterns shifted over time in ways that would be consistent with a positive effect of the intervention.

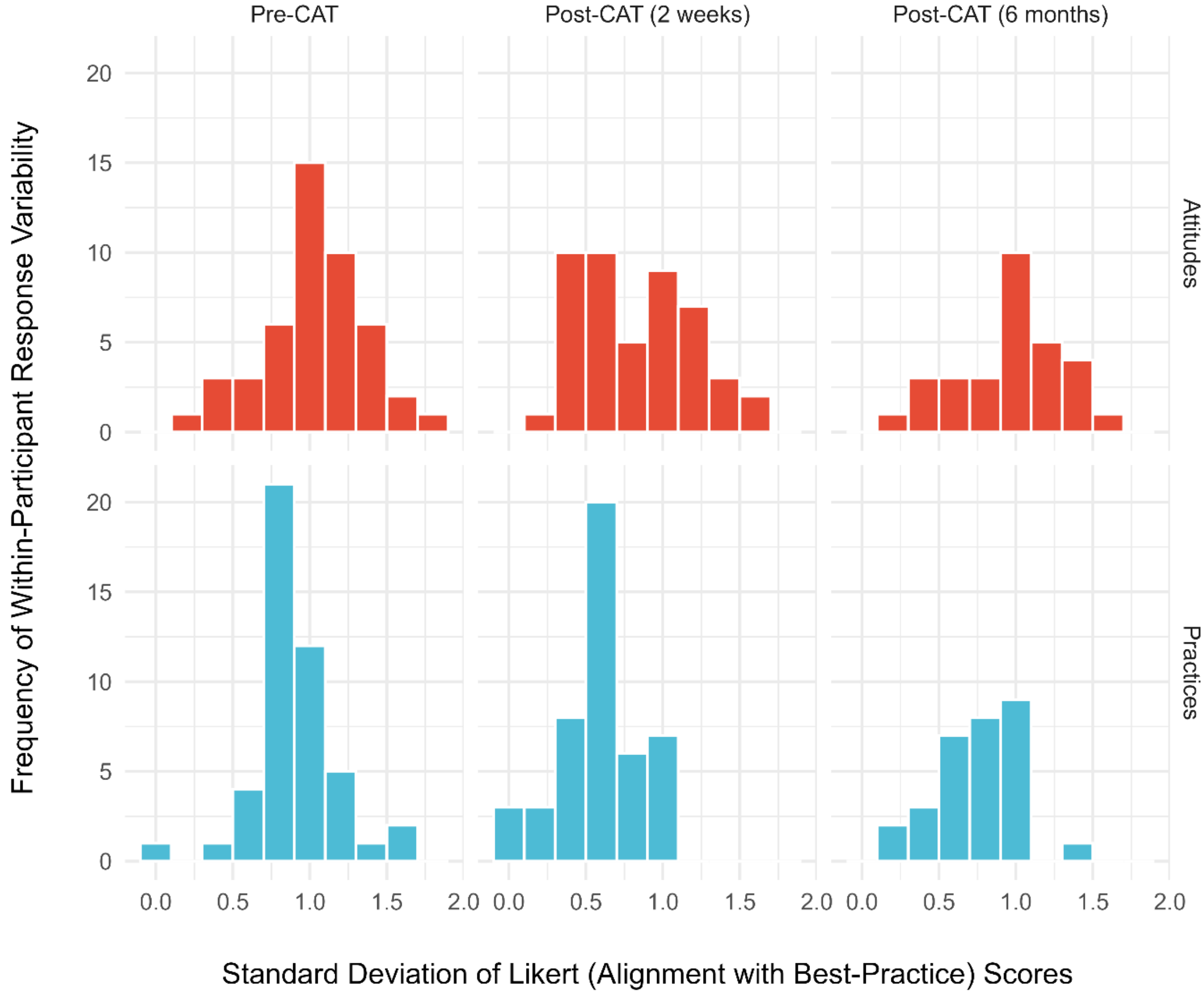

Figure S2. Distribution of Within-Participant Response Variability by Question Domain and Condition. Higher values indicate that a participant gave more varied responses across questions within a domain. Lower values suggest consistent scoring across items of the same type (attitudes or practices) by the same participant.

Text T2. Participant-Level Response Variability

Within-participant variability was calculated across questions in each domain and timepoint to evaluate engagement. Low variability can indicate potential disengagement or response fatigue, while moderate variability suggests thoughtful differentiation.

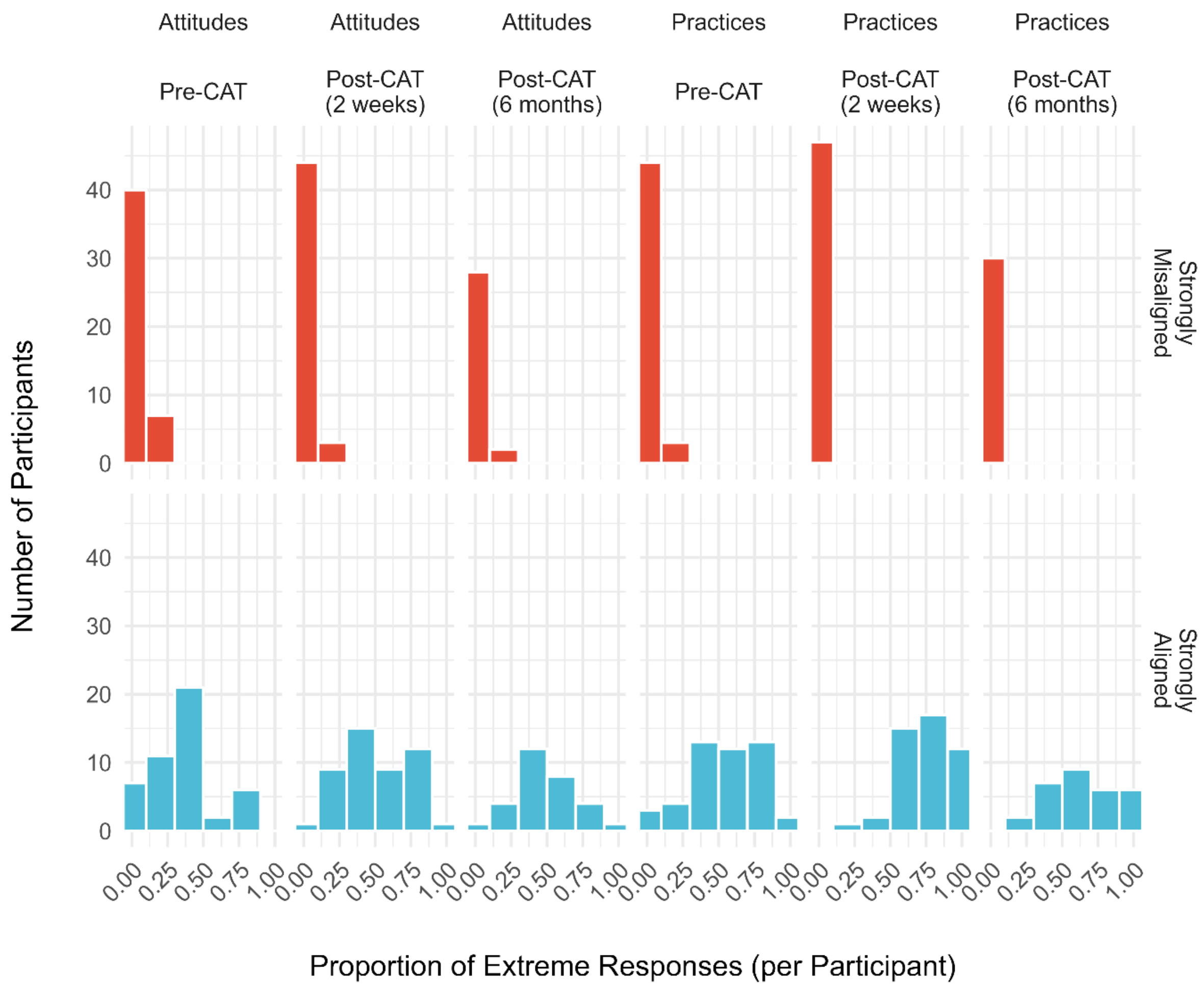

Figure S3. Proportion of Extreme Responses per Participant by Domain and Condition. Histograms show the distribution of participant-level proportions of “Strongly Agree” (top row) and “Strongly Disagree” (bottom row) responses across attitude and practice questions at each timepoint. Higher proportions may indicate ceiling or floor effects, while lower proportions suggest more nuanced or varied responding. Binwidth = 0.2.

Text T3. Proportion of Extreme Responses

We calculated the proportion of “Strongly Agree” and “Strongly Disagree” responses per participant to identify potential overuse of extreme categories. This helped flag questions that may have been too leading, too polarising, or insufficiently nuanced to capture variation in participant views.

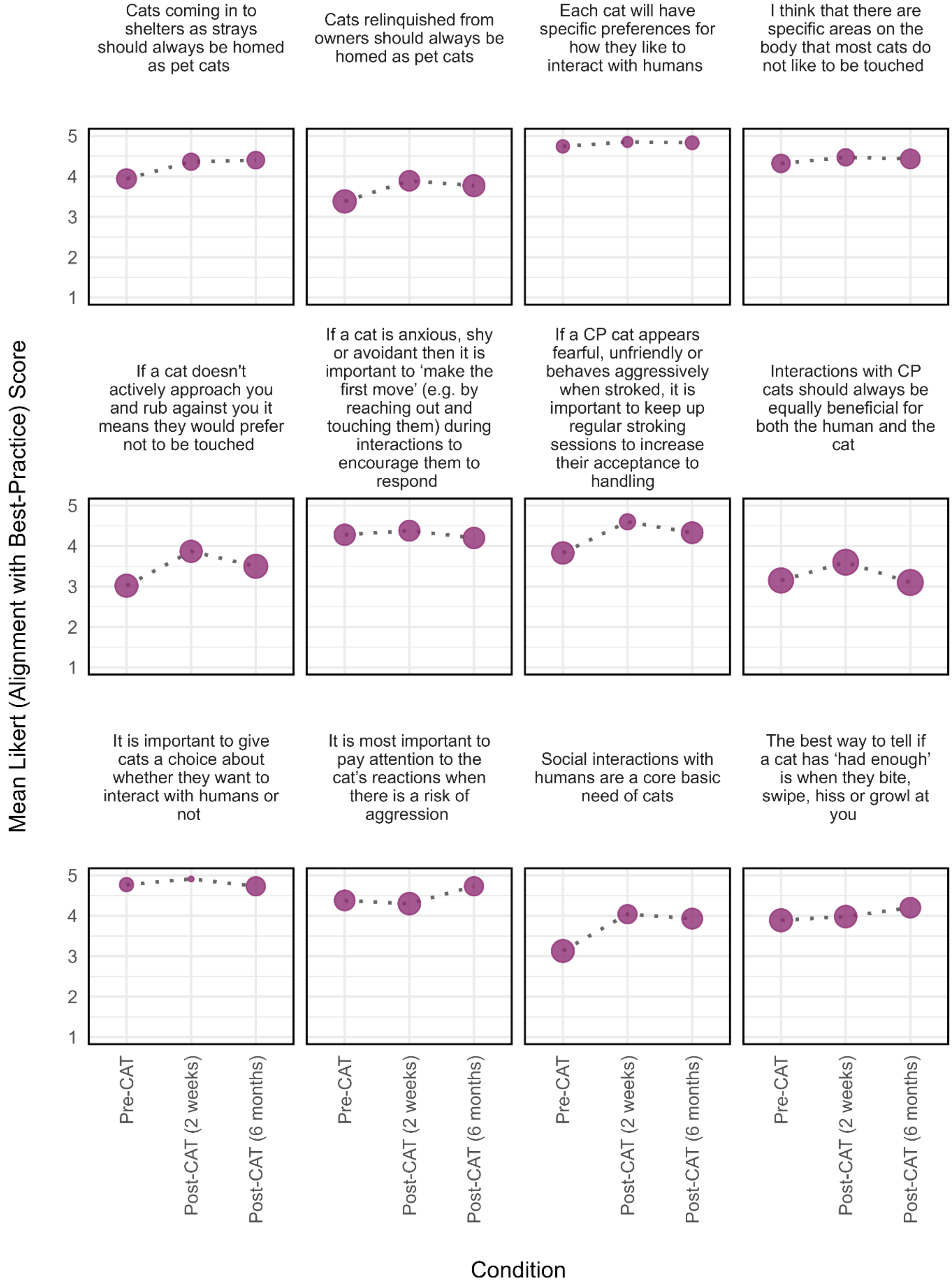

Figure S4. Question-Level Mean (Alignment with Best-Practice) Scores Over Time: Attitude Questions. Point size reflects variability (SD); dotted lines show temporal trajectory.

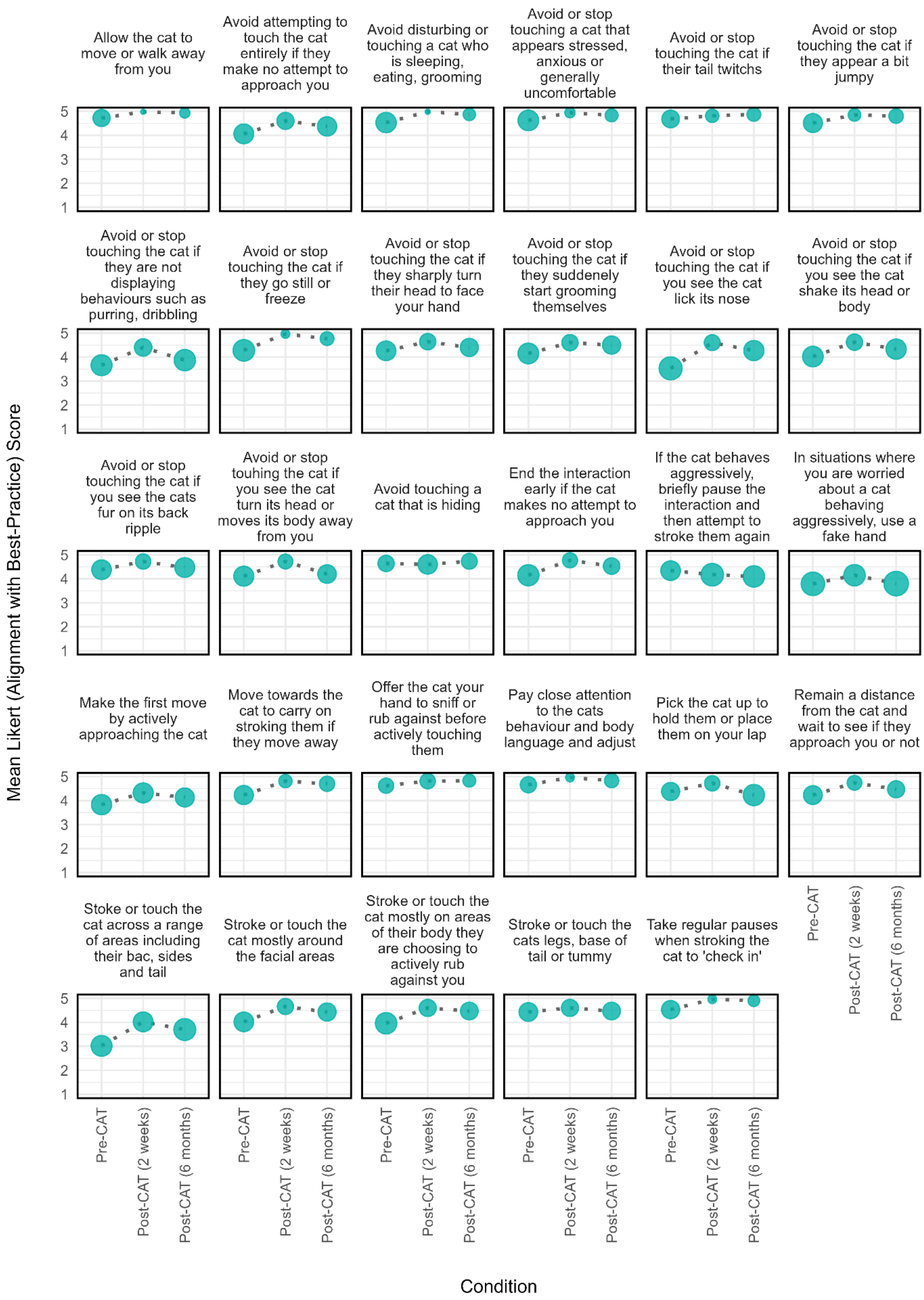

Figure S5. Question-Level Mean (Alignment with Best-Practice) Scores Over Time: Practices Questions. Point size reflects variability (SD); dotted lines show temporal trajectory.

Text T4. Question-Level Mean, Variability, and Temporal Trajectory

For each question we examined mean scores and variability across the three timepoints to understand how responses to that question changed, clustered, or remained stable. This helped us identify questions that were consistently endorsed (suggesting strong consensus), showed little variation (indicating limited discriminative value), or shifted meaningfully over time (suggesting responsiveness to the intervention). These patterns helped flag questions that may have been too obvious, too confusing, or too static to provide useful insight into participant attitudes or practices.

For question-level diagnostics, we used mean scores to examine how responses to individual items changed over time. While medians were preferred for participant-level modelling to respect the ordinal nature of Likert-scale data, means were more appropriate here (S4, S5, S6; T4) because the goal was to explore group-level patterns rather than individual-level inference. Means allowed us to detect subtle shifts in central tendency across timepoints, compare relative endorsement across items, and visualise temporal trajectories. This approach also enabled the use of standard deviation as a measure of response variability, helping to flag questions that were consistently endorsed, poorly discriminating, or highly responsive to the intervention. In this context, the interpretive value of means outweighed the limitations of treating ordinal data as interval-level.

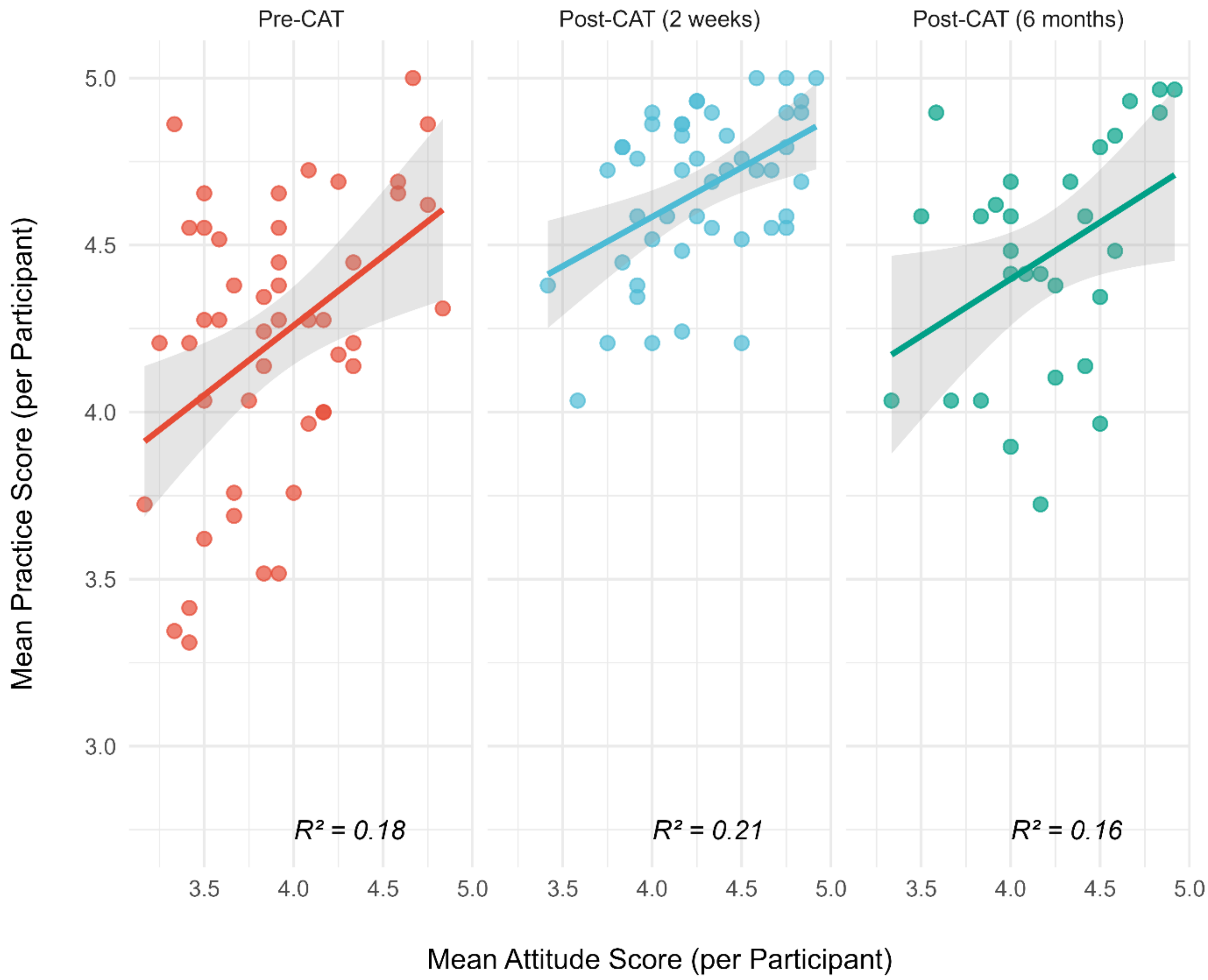

Figure S6. Participant-Level Alignment Between Attitude and Practice Scores. Each point represents a participant's mean scores across attitude and practice items. Scatterplots show participant-level mean scores for attitude (x-axis) and practice (y-axis) domains, faceted by timepoint. Each point represents a single participant. Linear trend lines with 95% confidence intervals illustrate the degree of alignment between cognitive endorsement (attitudes) and reported behaviours (practices). Model fit was modest across timepoints, with R^2 values indicating a weak but consistent association between attitude and practice scores throughout the intervention period.

Text T5. Cohort Comparison Modelling

To formally test differences between the long-term (T1, T2, and T3) and short-term (T1 and T2) participant cohorts, we fitted a binomial generalised linear model (GLM) with cohort membership as the binary outcome. Fixed effects included baseline median attitude and practice scores, age category, work status (employee vs volunteer), prior cat behaviour training, and service length. Each participant contributed a single row, so random intercepts were not required.

The 19 participants who remained in the 6-month follow-up (T3) did not differ significantly in their characteristics from the original 47 participants who completed T1 and T2 (Table S1). However, in terms of absolute values, there were some modest (non-significant) differences, as follows. Higher attitude and practices scores were associated with increased odds of long-term participation (i.e. T3 cohort), suggesting greater alignment with best-practice principles (i.e., higher BPA ratings) among retained participants. Volunteer status and shorter service length also showed positive associations with long-term participation, while older age and prior cat behaviour training were linked to reduced odds of long-term follow-up. Although there was no significant evidence for these effects (see Table S1 below), the overall direction and magnitude of estimates suggest that long-term participants may have been more engaged (returning for T3) and attitudinally aligned (higher mean attitude scores) than those who did not opt into extended participation.

Table S1. Type II Analysis of Deviance (Chisq) comparing participant cohorts (n=47 vs n=19)

Term	Chi-Square	Df	p-value
Median Attitude Score at baseline (T1)	1.032	1.000	0.310
Median Practice Score at baseline (T1)	0.880	1.000	0.348
Age Category	0.001	1.000	0.979
Work Status	1.012	1.000	0.314
Prior Cat Behaviour Training	1.866	1.000	0.172
Service Length	0.017	1.000	0.895
