# Supplementary material for: Sustained improvements in human–cat interaction in the shelter sector: a study of a best-practice educational intervention
Source: Front Vet Sci. 2026 Aug 10;13:1880180. doi: 10.3389/fvets.2026.1880180 (PMC13500327; doi:10.3389/fvets.2026.1880180)

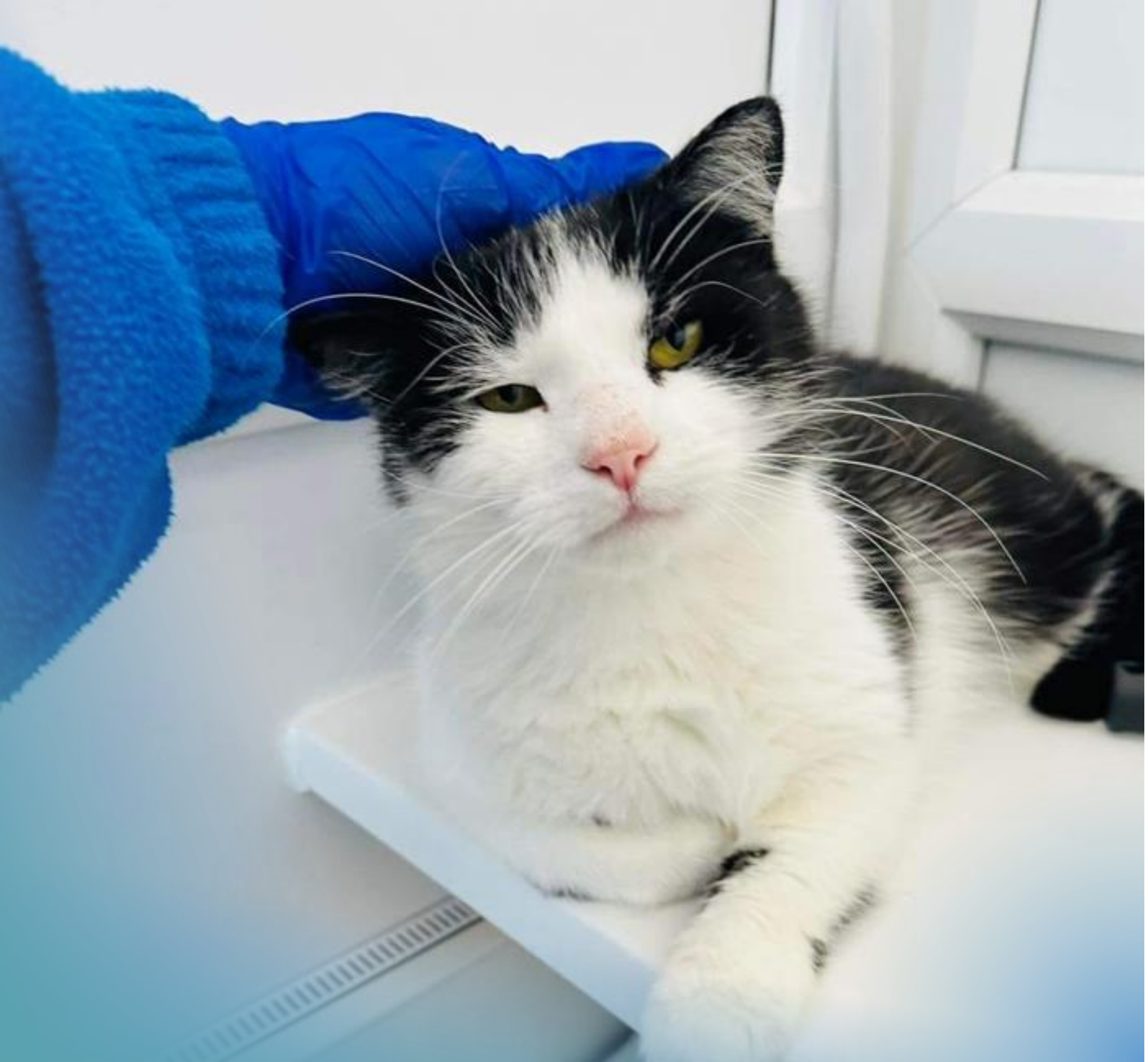

Feline Interactions & Approach

Cat Welfare Learning Workshop

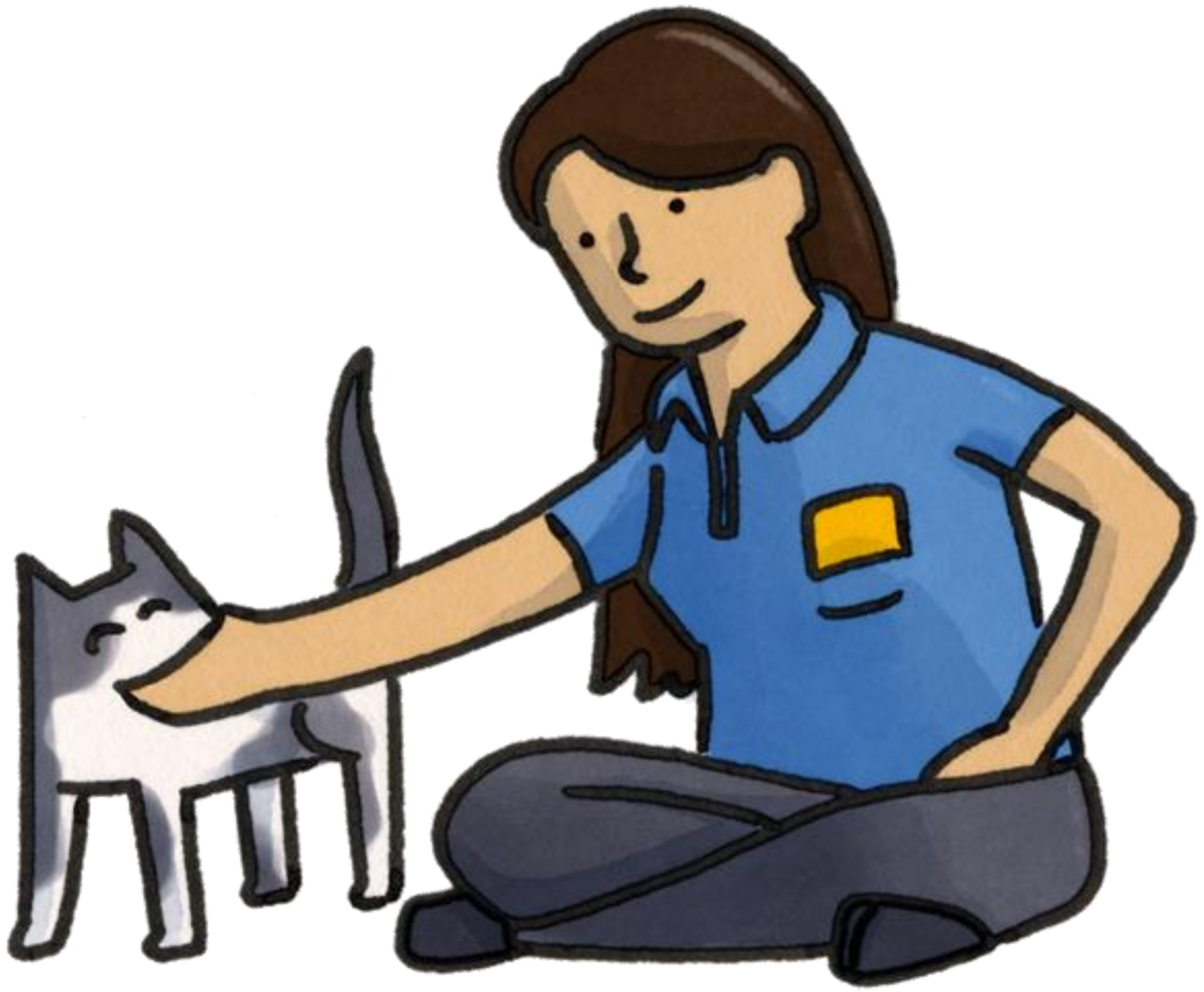

What are we looking at today?

- Explore our relationship with cats and how that affects our interactions with them
- Recognise how stress in cats can impact our interactions with cats
- Identify the importance of positive interactions and approach with cats in our care
- Recognise and adapt to a cat's body language

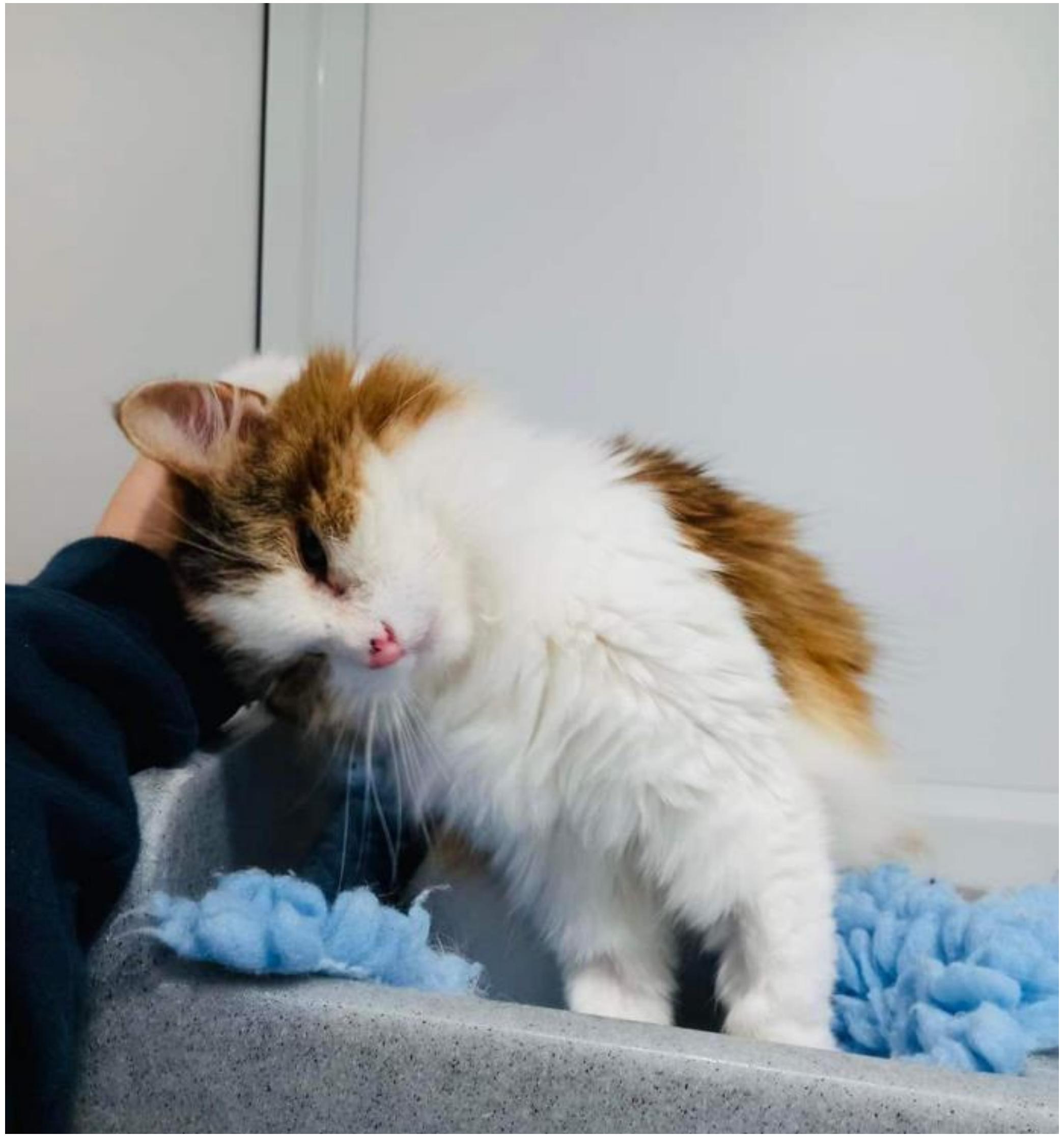

What is an interaction?

- *Communication or direct involvement with someone or something*
- *An action or influence*

What are some examples of intentional and unintentional interactions?

Modern day cats

How have our relationships changed with cats over the years?

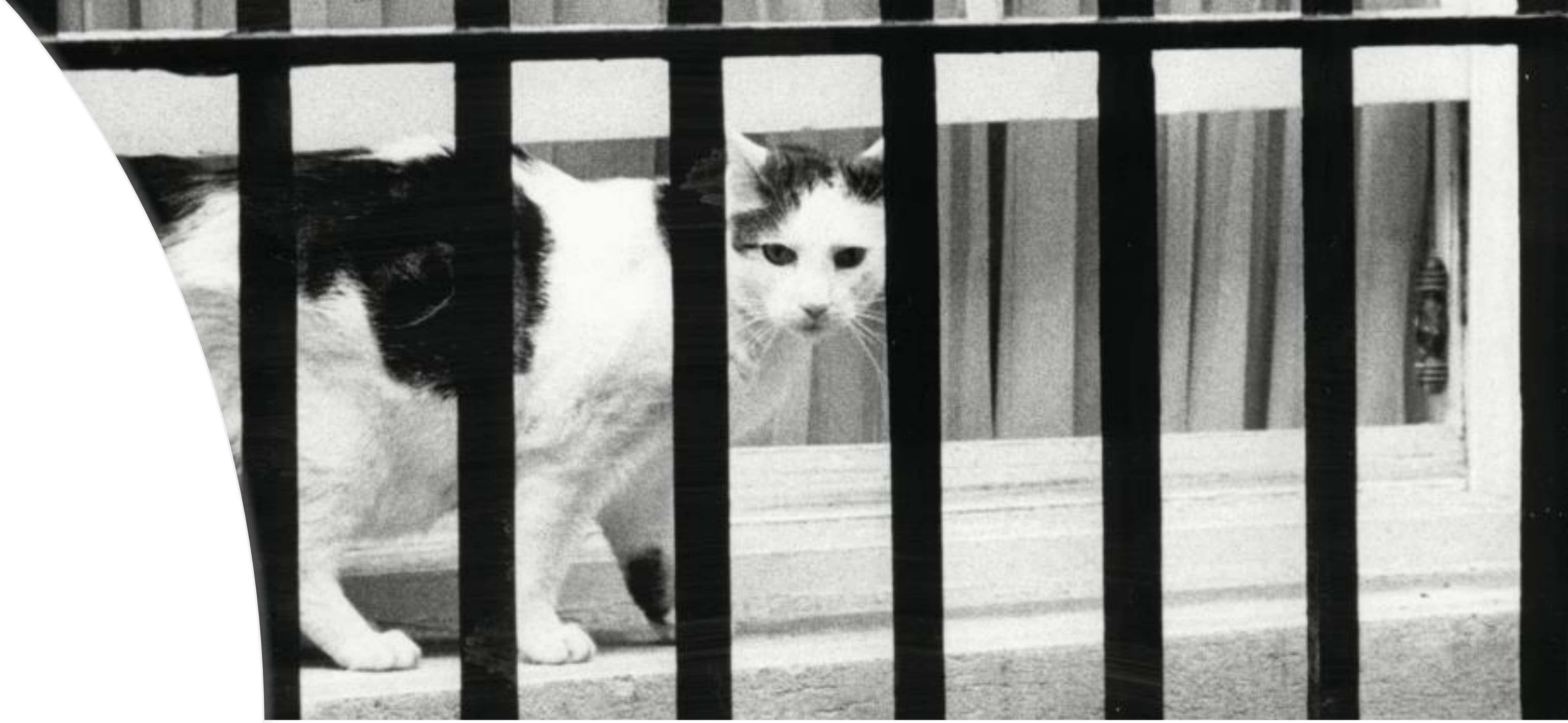

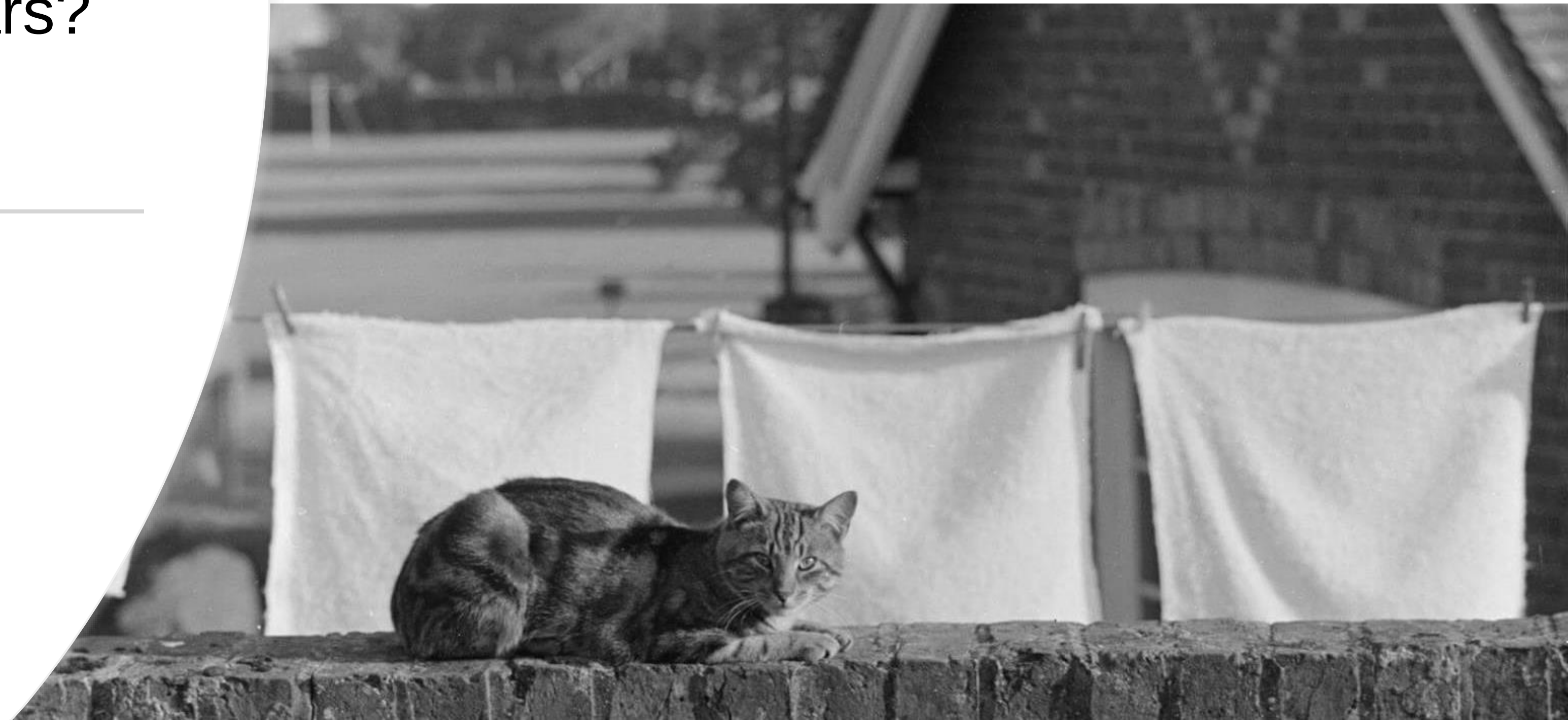

Great expectations

- **Humans:**
  - Social
  - Tactile
  - Verbal communication
  - Control

- **Cats:**
  - Solitary
  - Non-tactile
  - Sense led communication
  - Lack of control

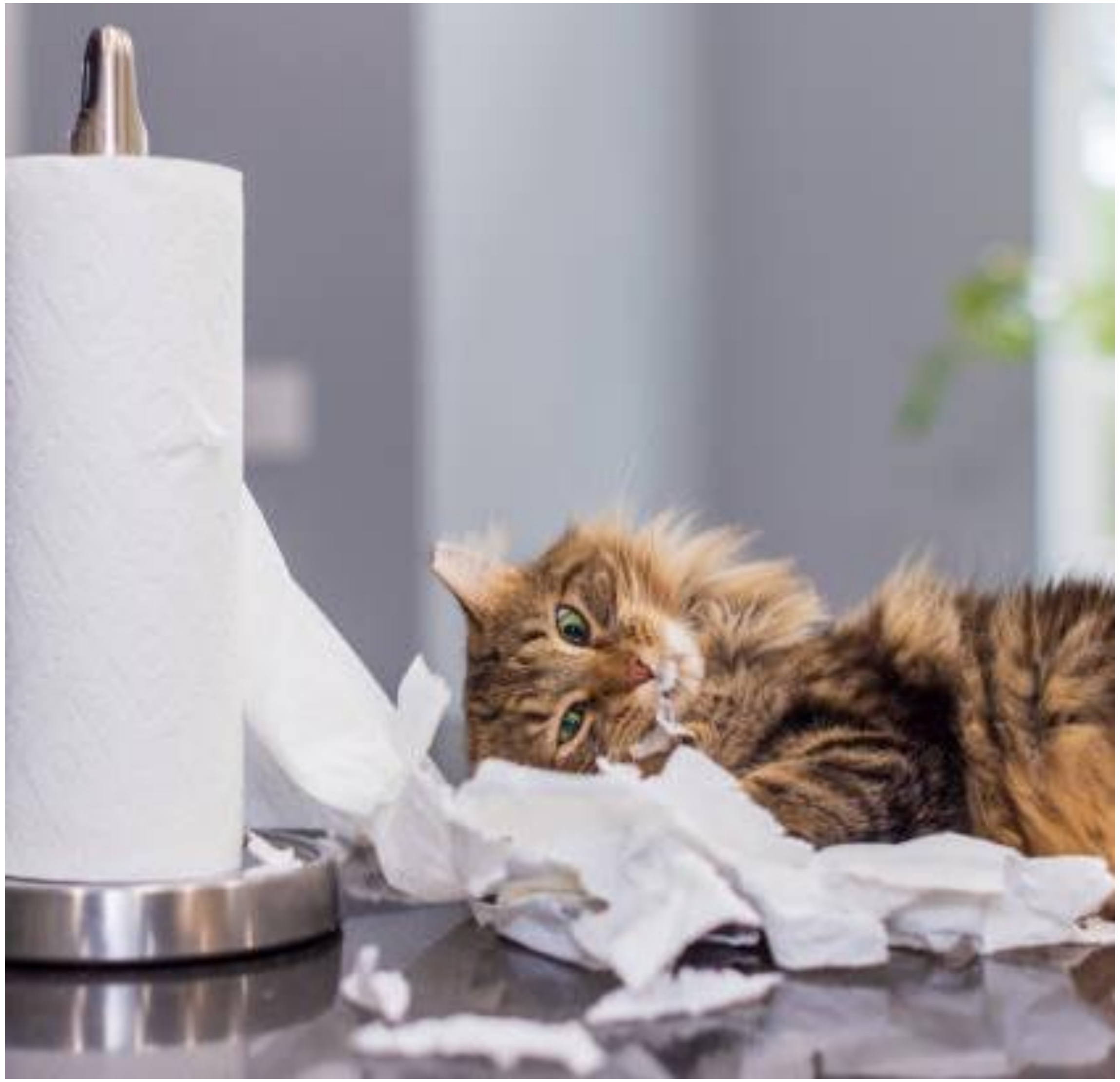

The ‘inbetweeners’

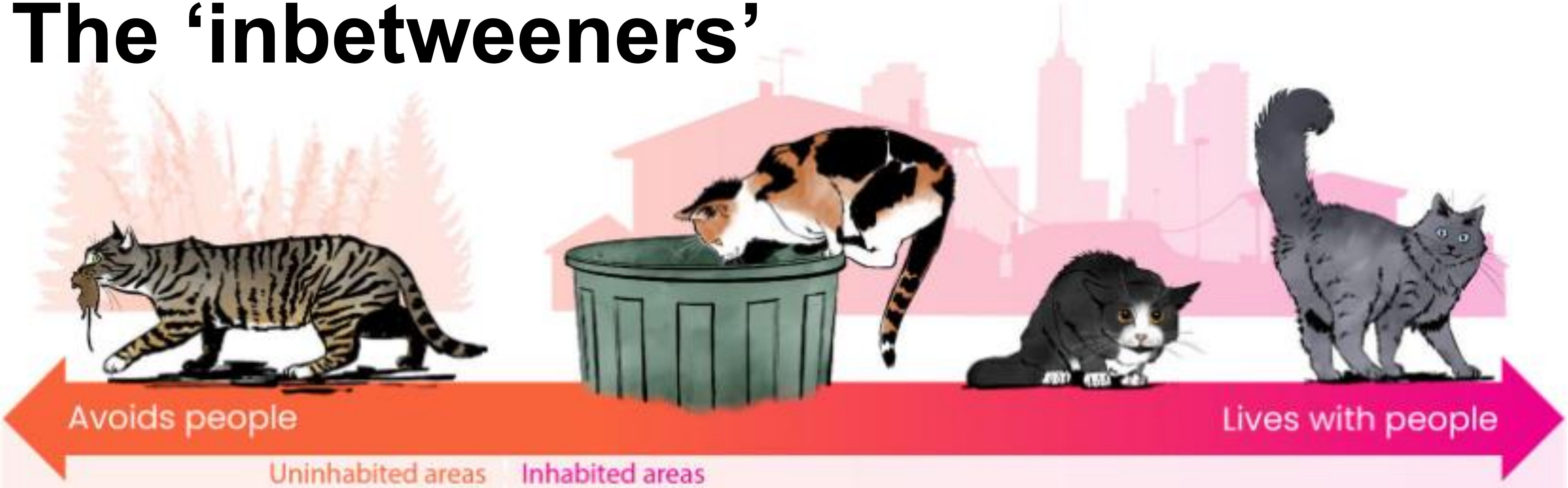

Feral Cat	Street Cat	Inbetweener Cat	Pet Cat
Adapted to free-roaming	Adapted to free-roaming	Not adapted to free-roaming or life in domestic home	Adapted to life in domestic home
TNR, if practical given population density	TNR	Alternative lifestyle (Facilitated by homing organisations)	Homing Centre Foster Care

What might impact interactions?

- Environment
- Age & health status
- Previous experiences
- Timing
- Communication
- Stress

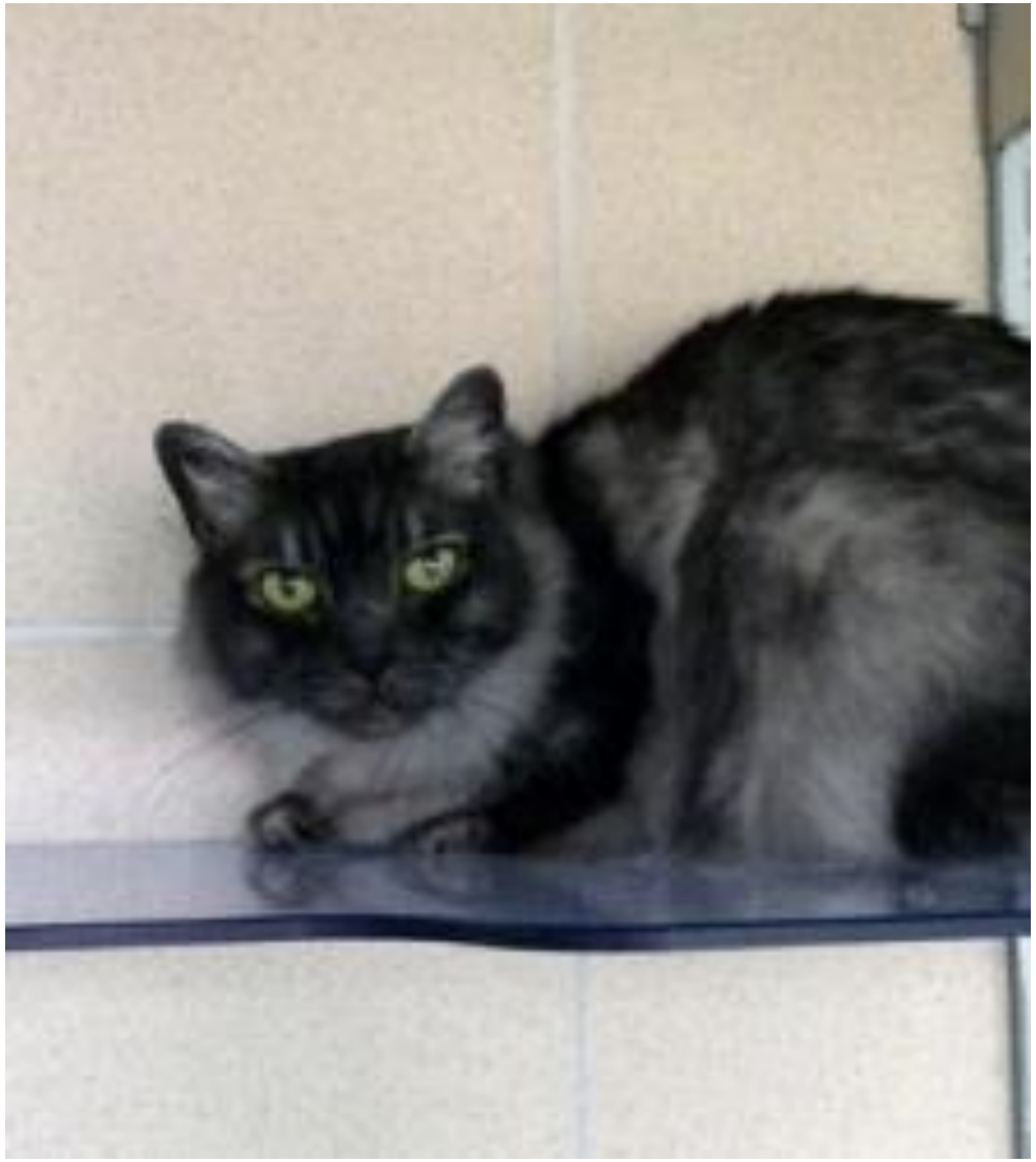

Stress

"The behavioural, physiological and emotional response used to attempt to cope with challenges within the environment".

Behaviour Guide pg.111

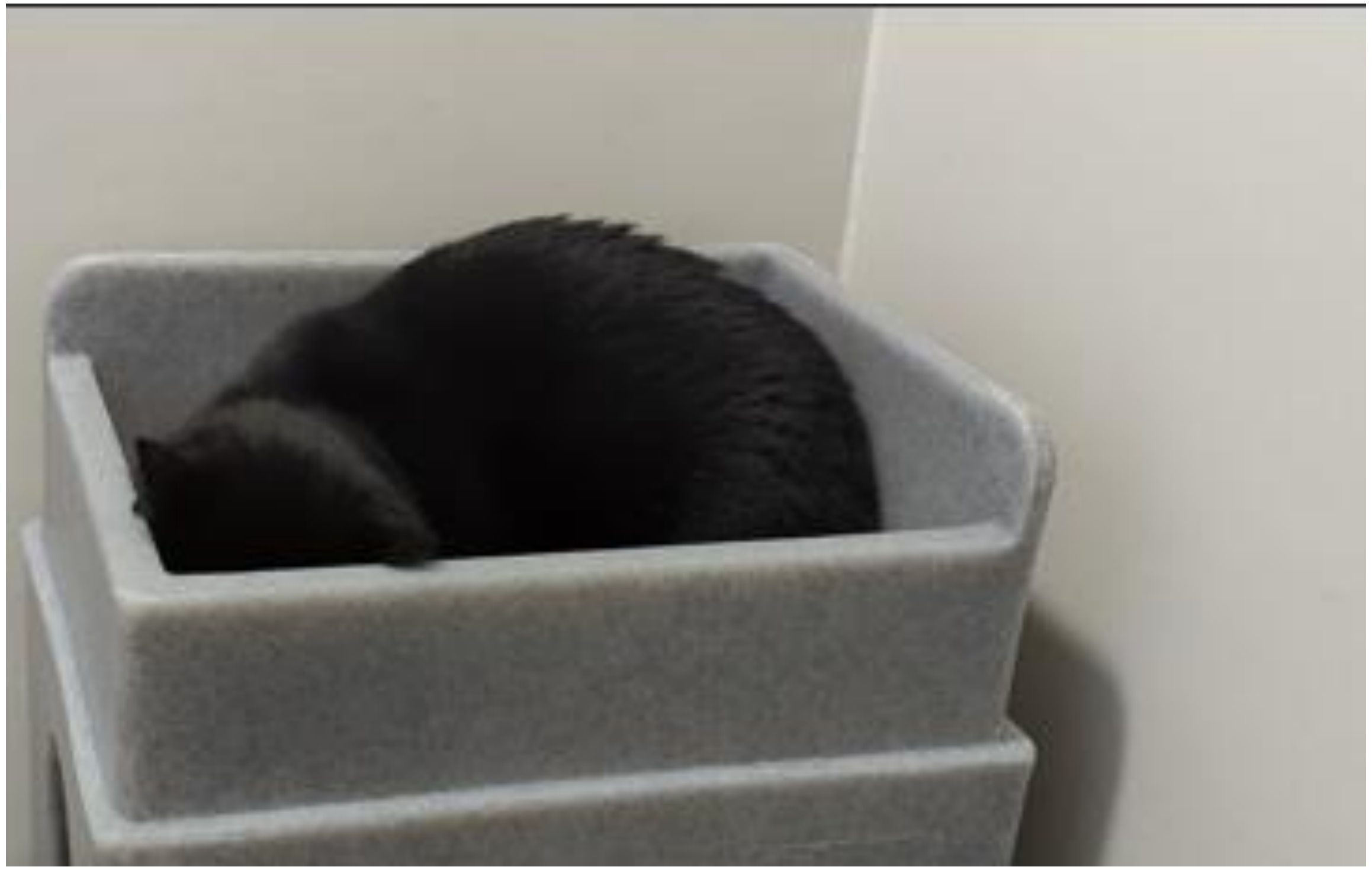

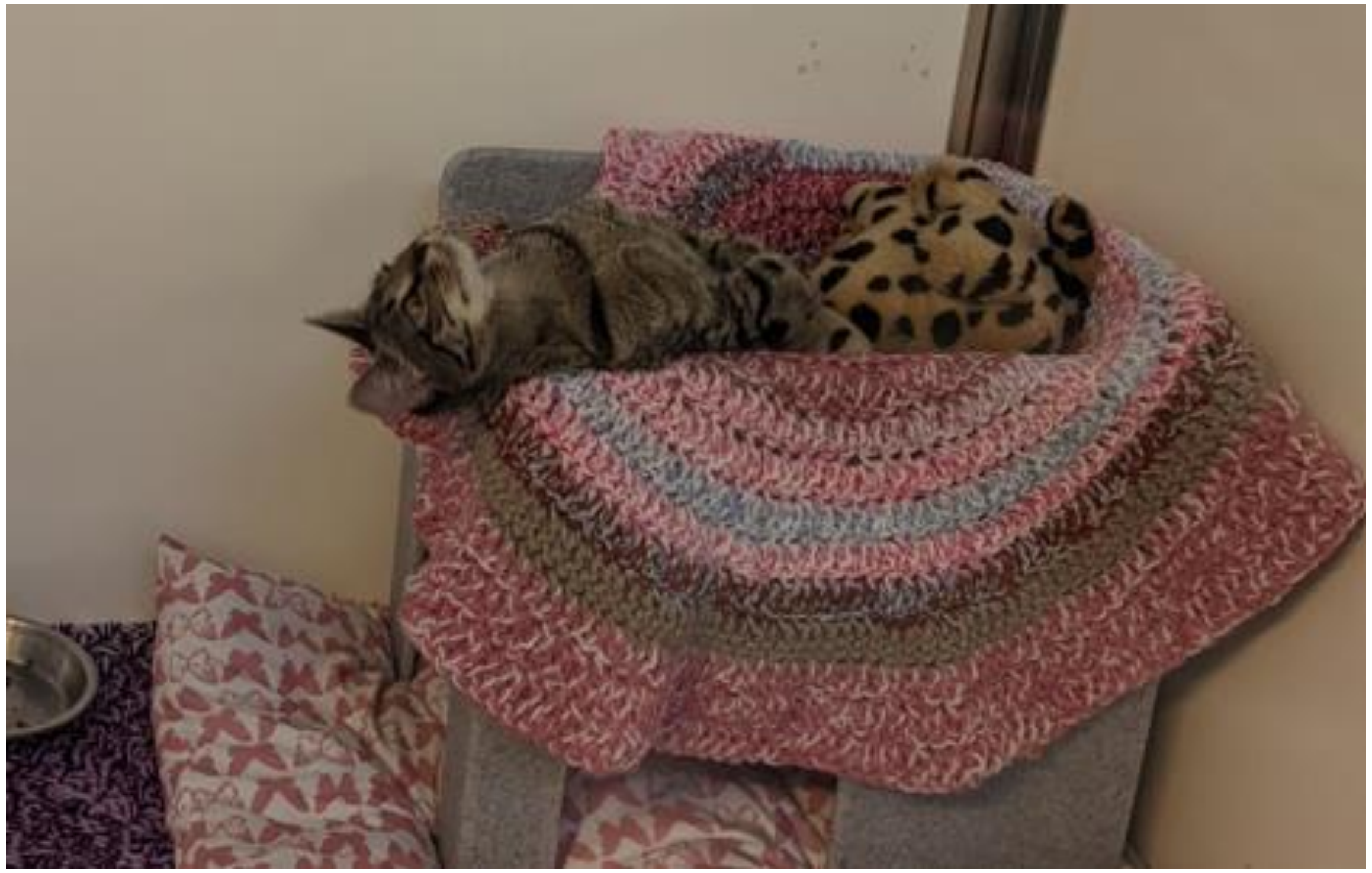

Trigger stacking

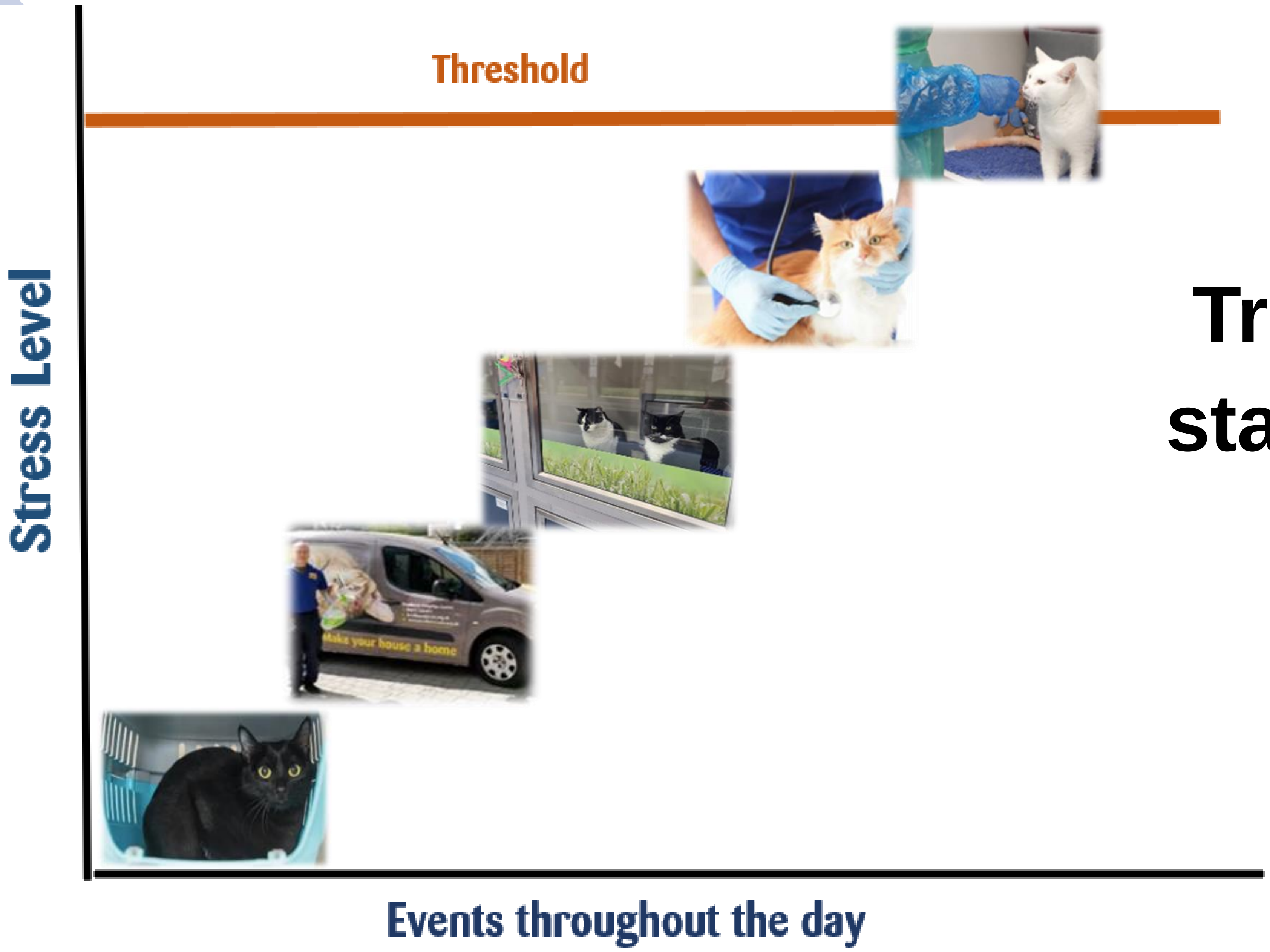

Natural Behaviours

The 4 F's: What responses do cats use in stressful situations?

F

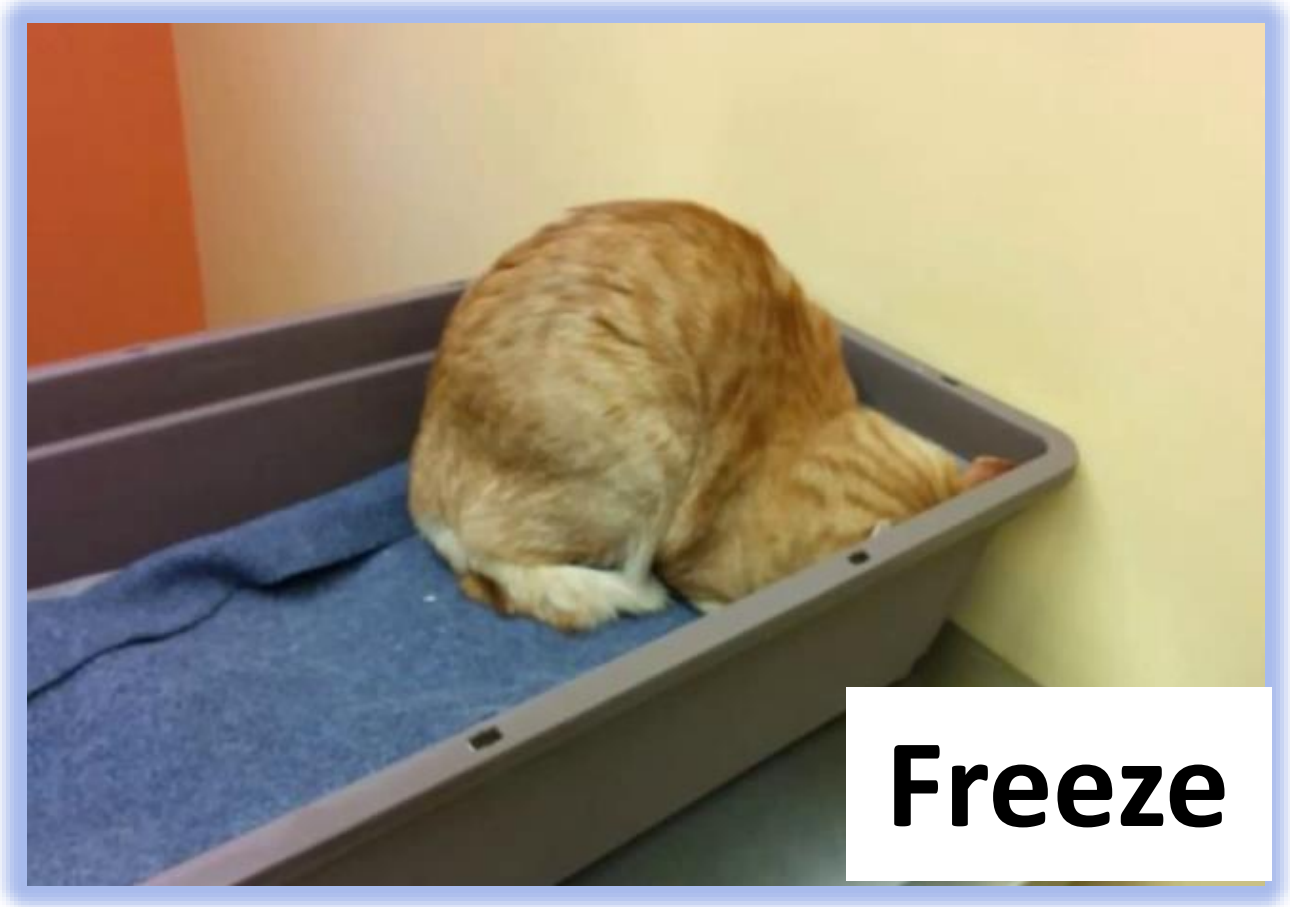

F

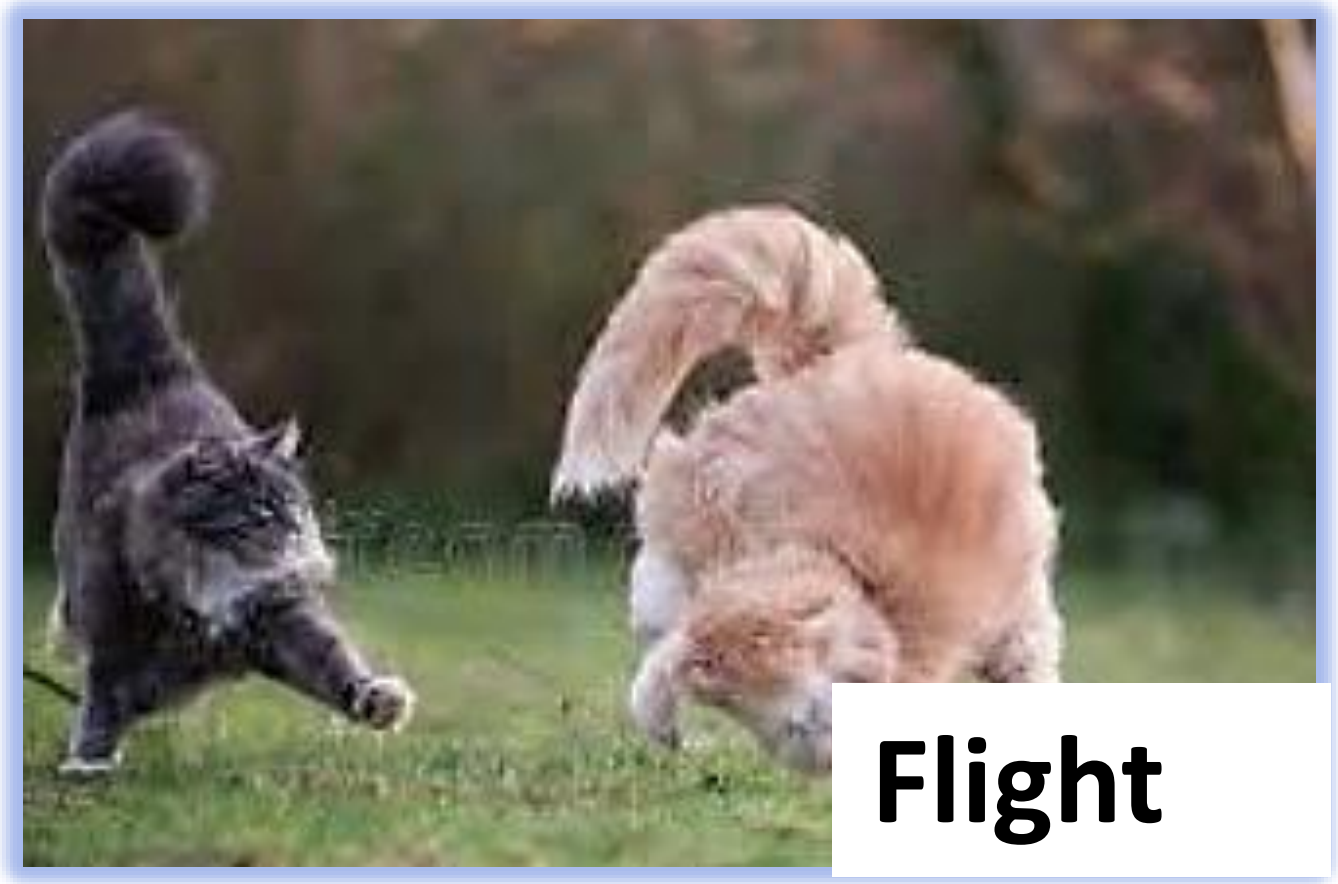

F

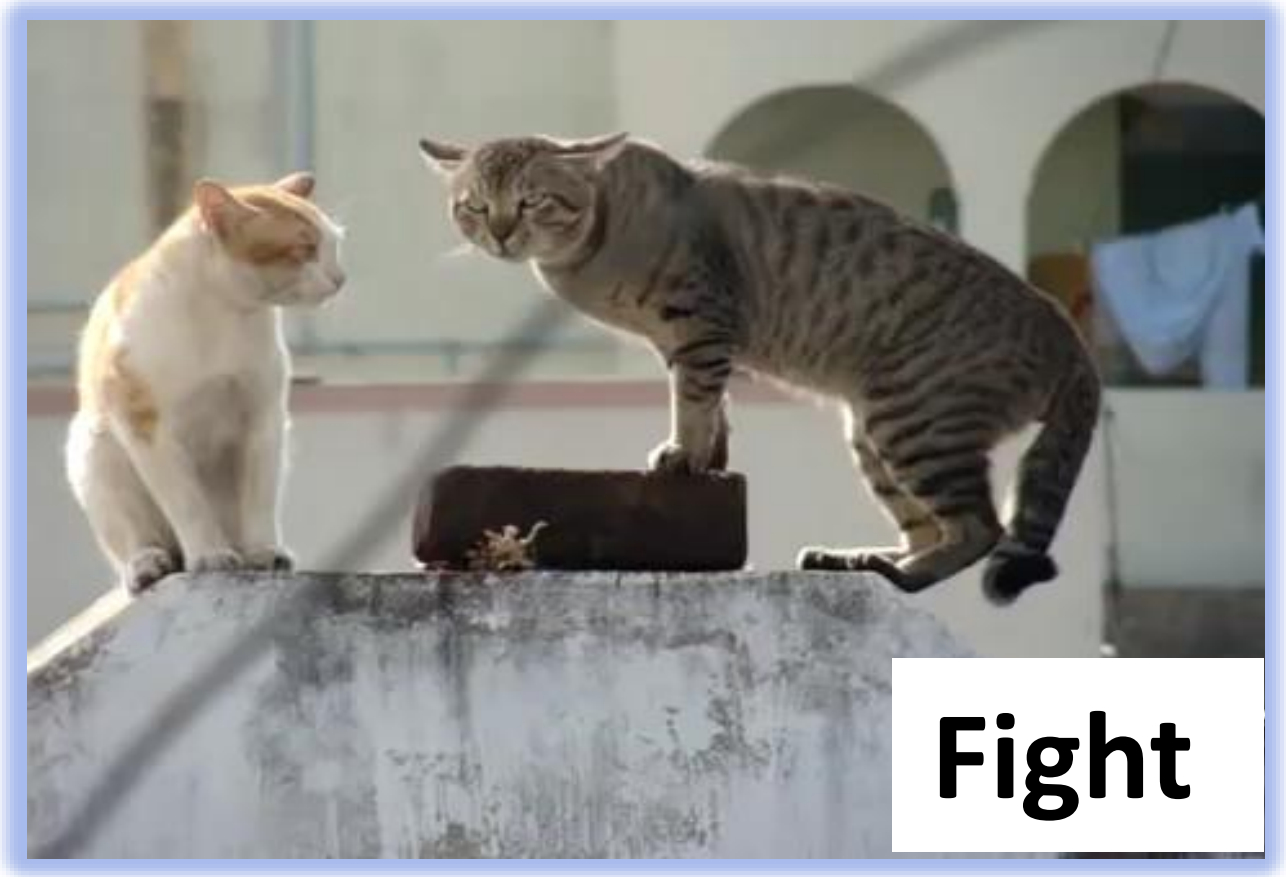

F

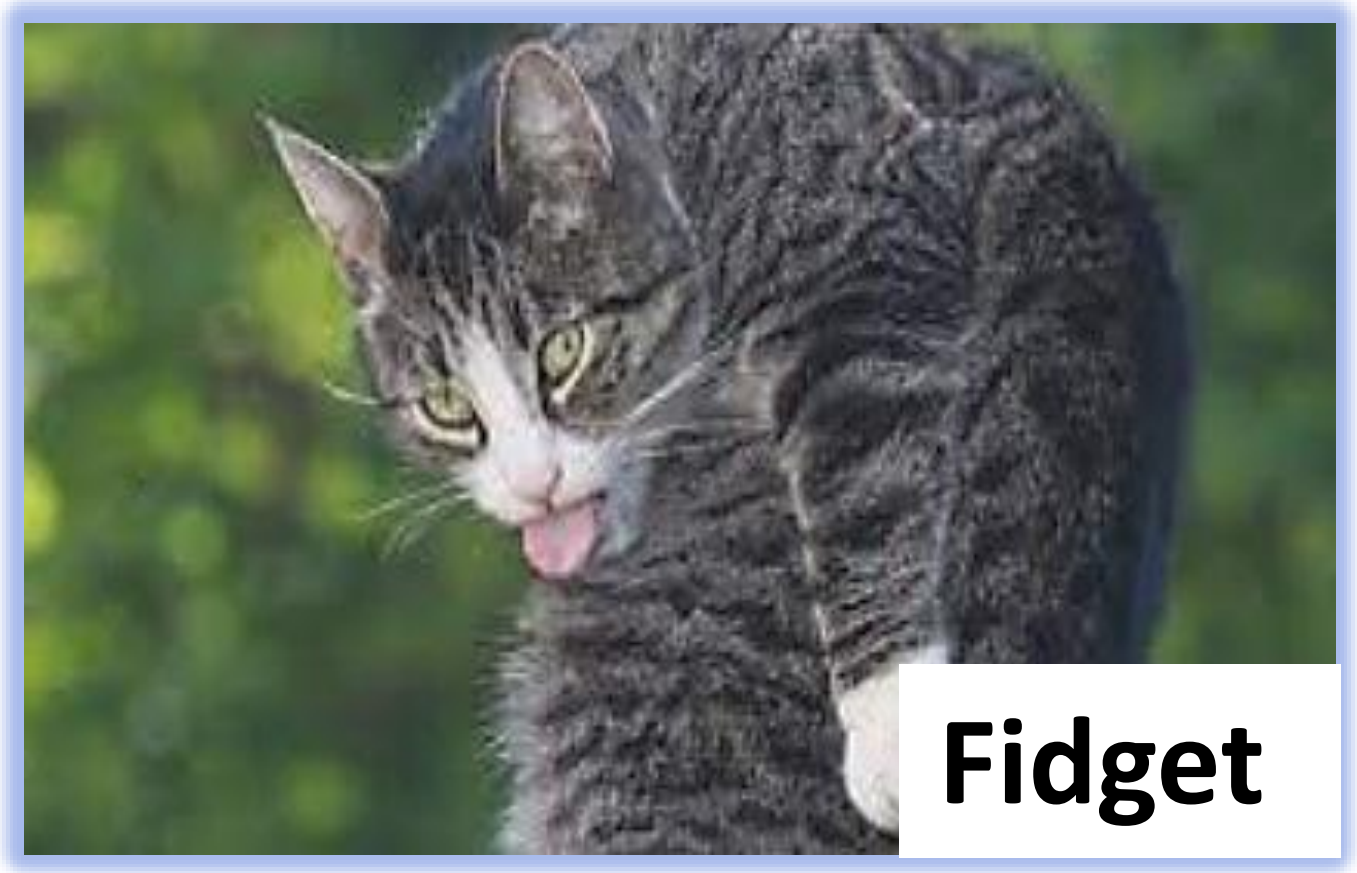

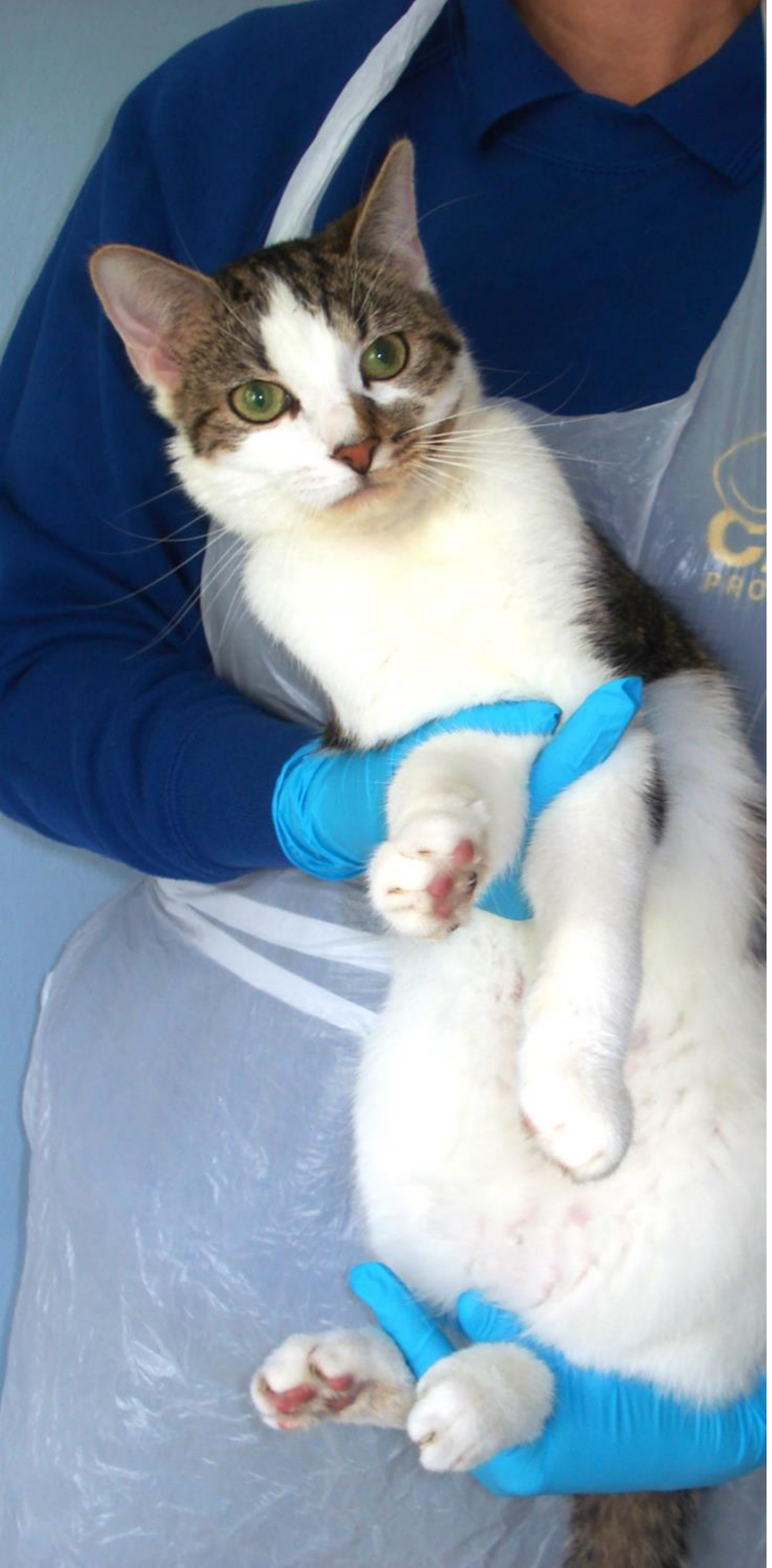

Positive Interactions

Why are we interacting with cats in our care?

Is it task or social?

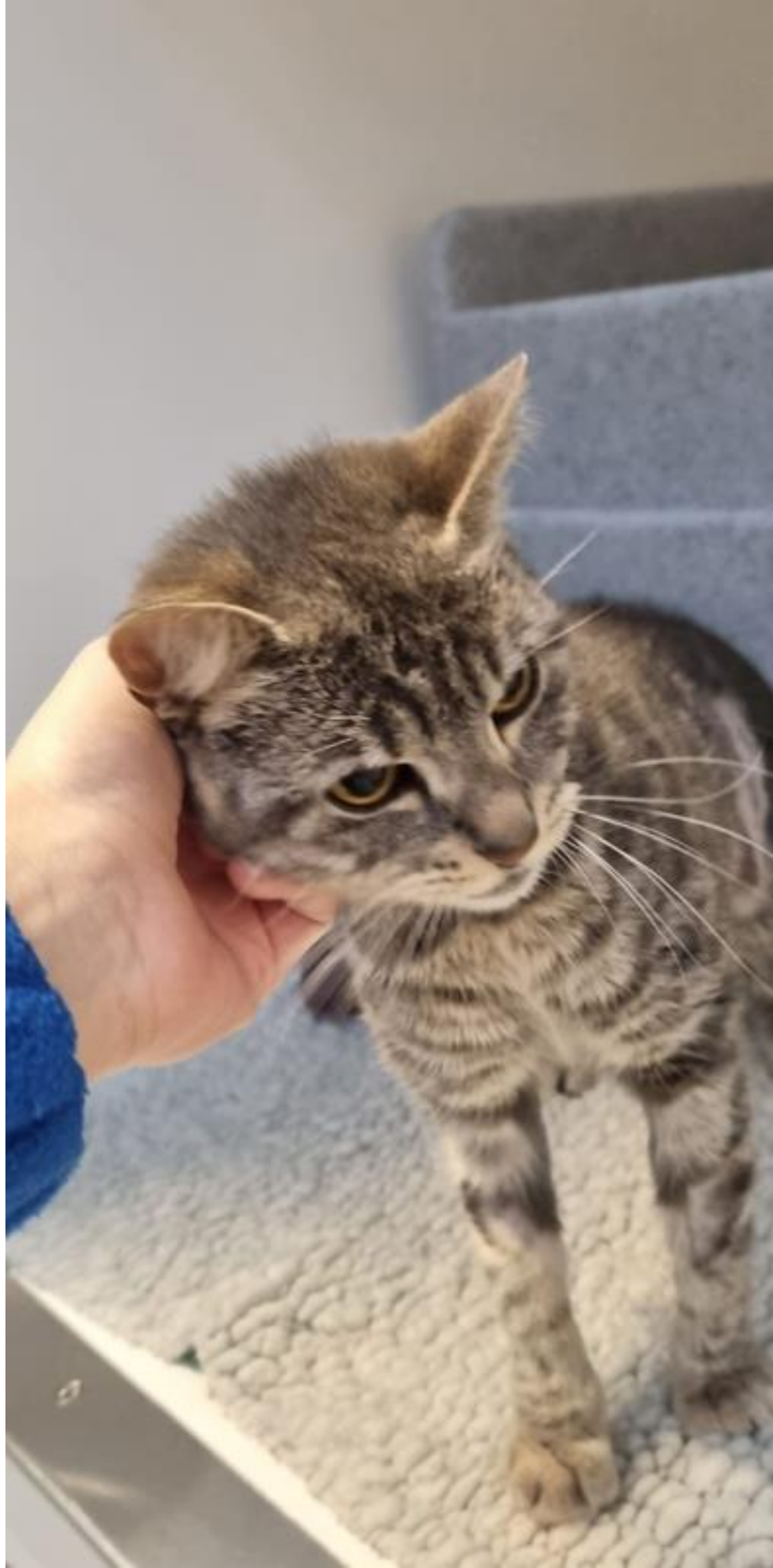

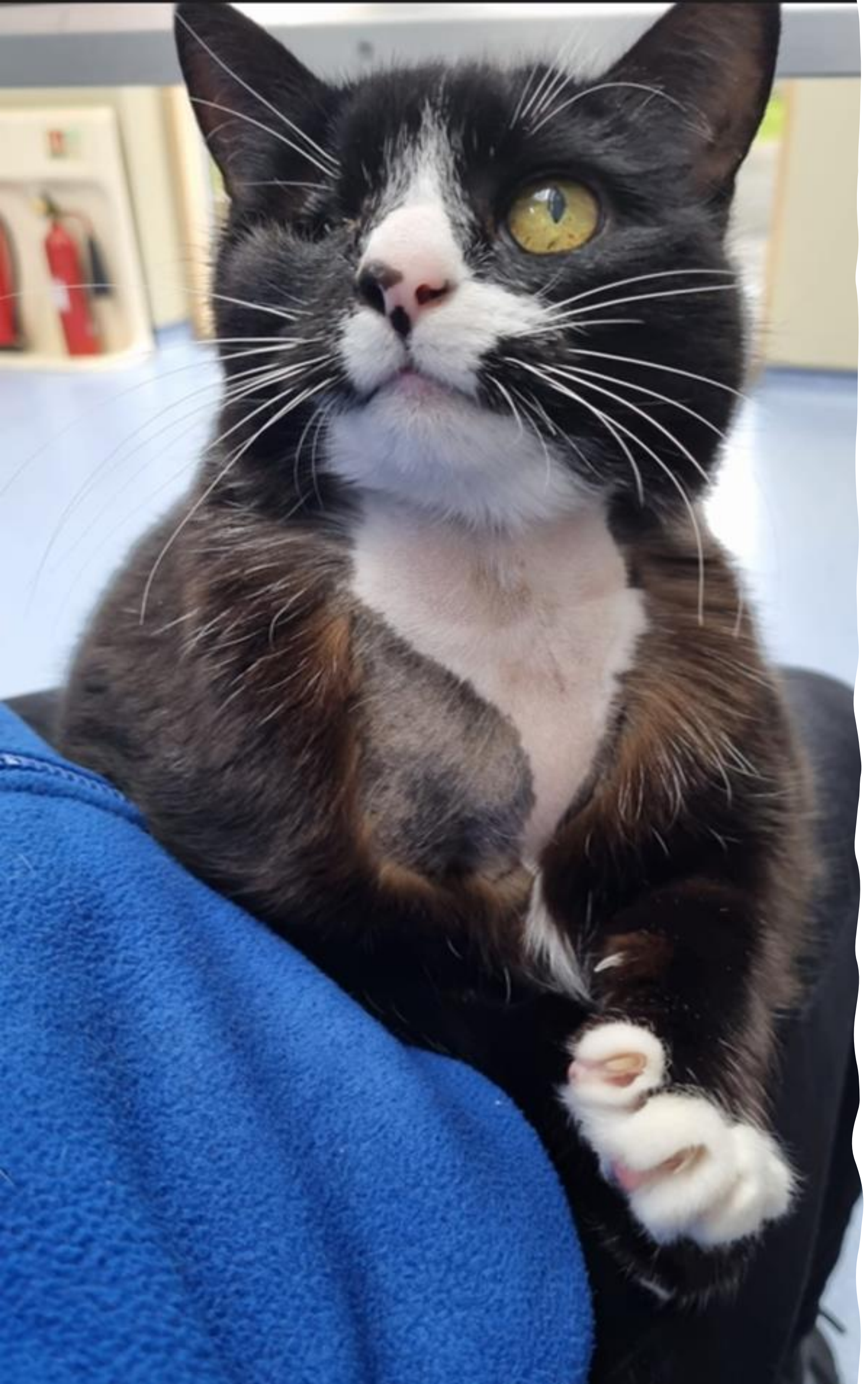

CAT

- C: Did I give the cat a **CHOICE** about whether or not they wanted to interact?
- A: Am I paying **ATTENTION** and looking out for any subtle signs that the cat is uncomfortable?
- T: Where am I **TOUCHING** the cat and does the cat want me to keep touching?

Reference: Finka *et al* (2021) Providing Humans With Practical, Best Practice Handling Guidelines During Human-Cat Interactions Increases Cats' Affiliative Behaviour and Reduces Aggression and Signs of Conflict

C – Did I give the cat choice about whether they wanted to interact with me?

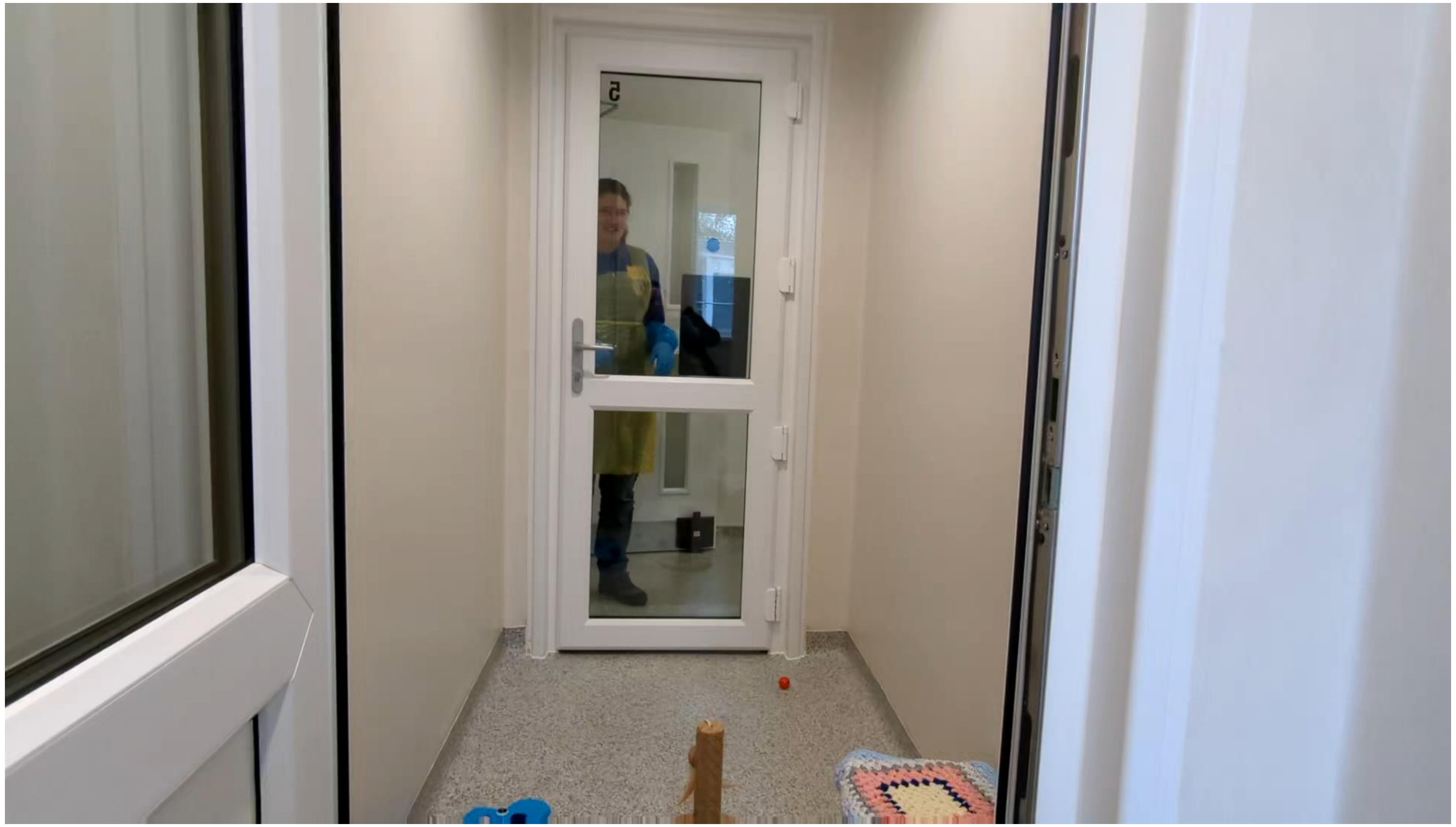

A - Am I paying attention and looking out for any subtle signs that the cat is uncomfortable?

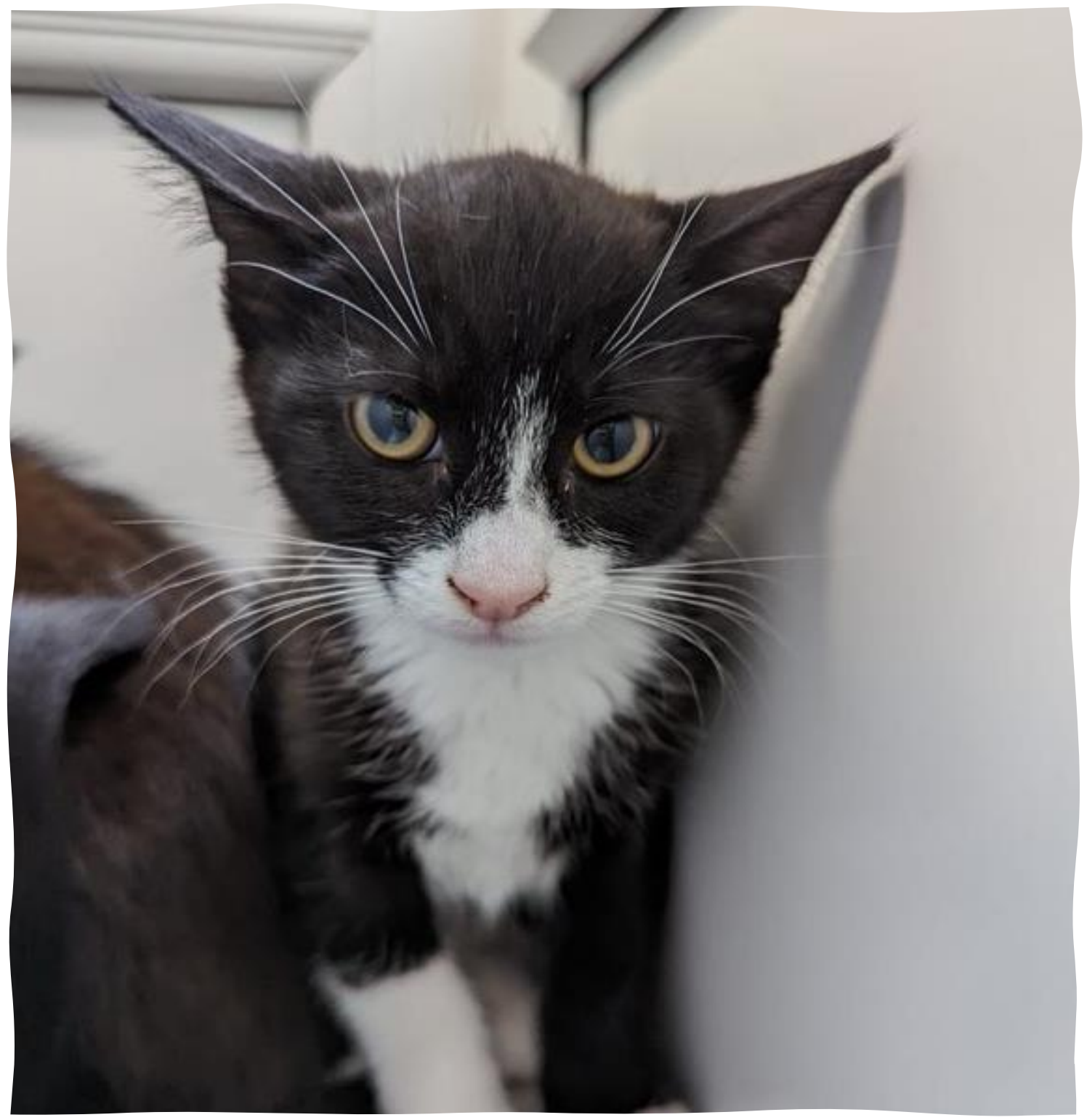

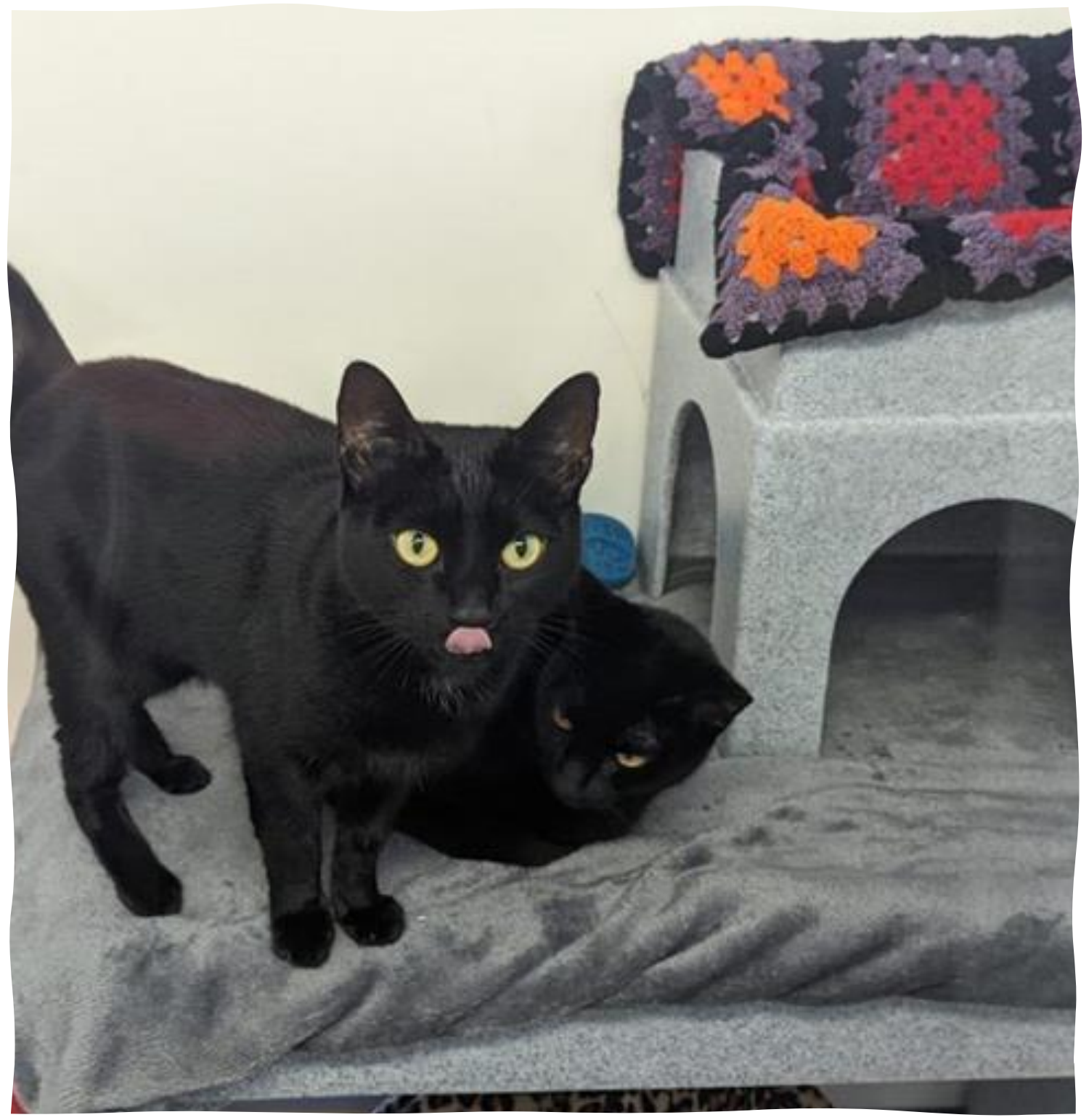

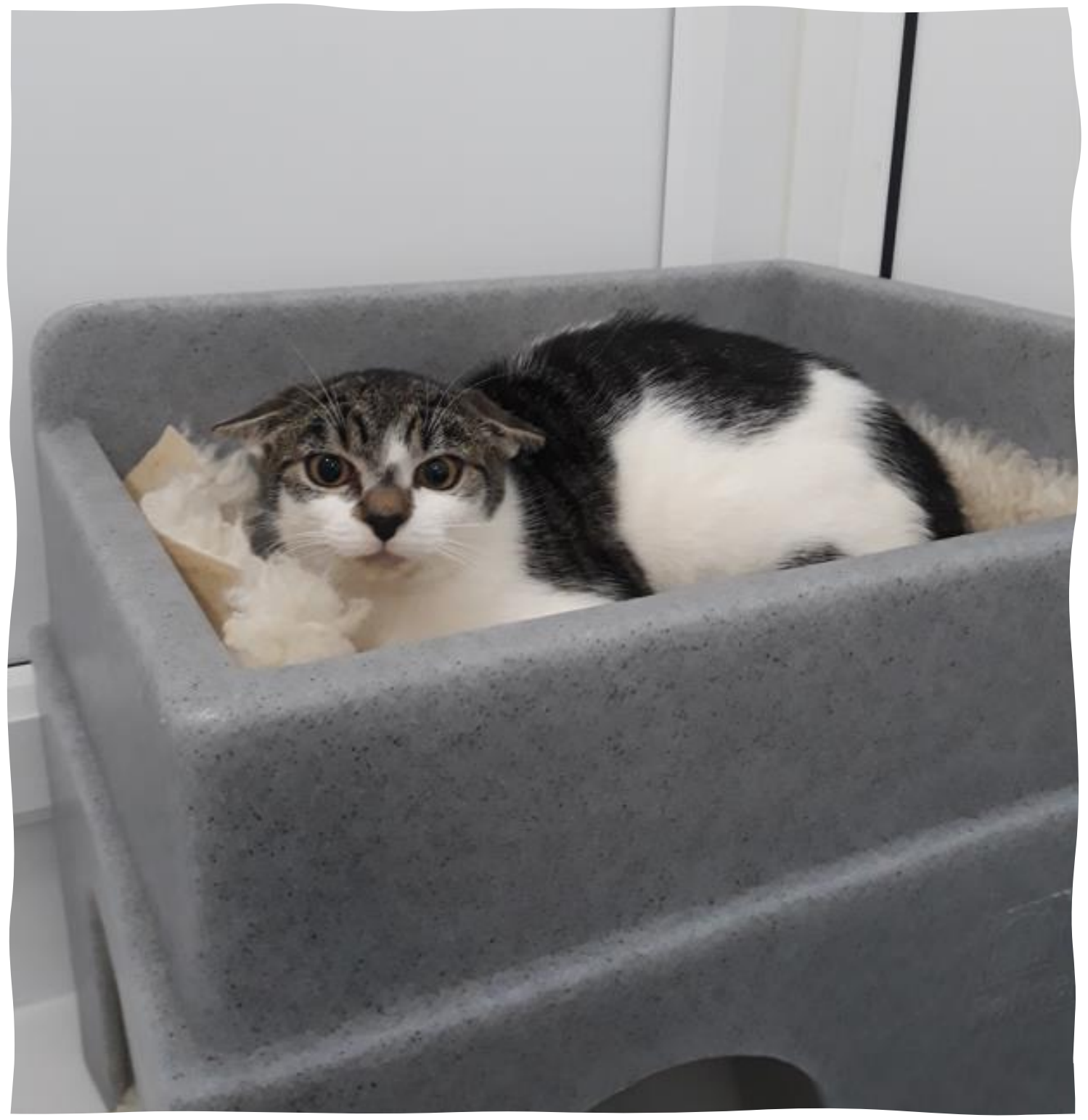

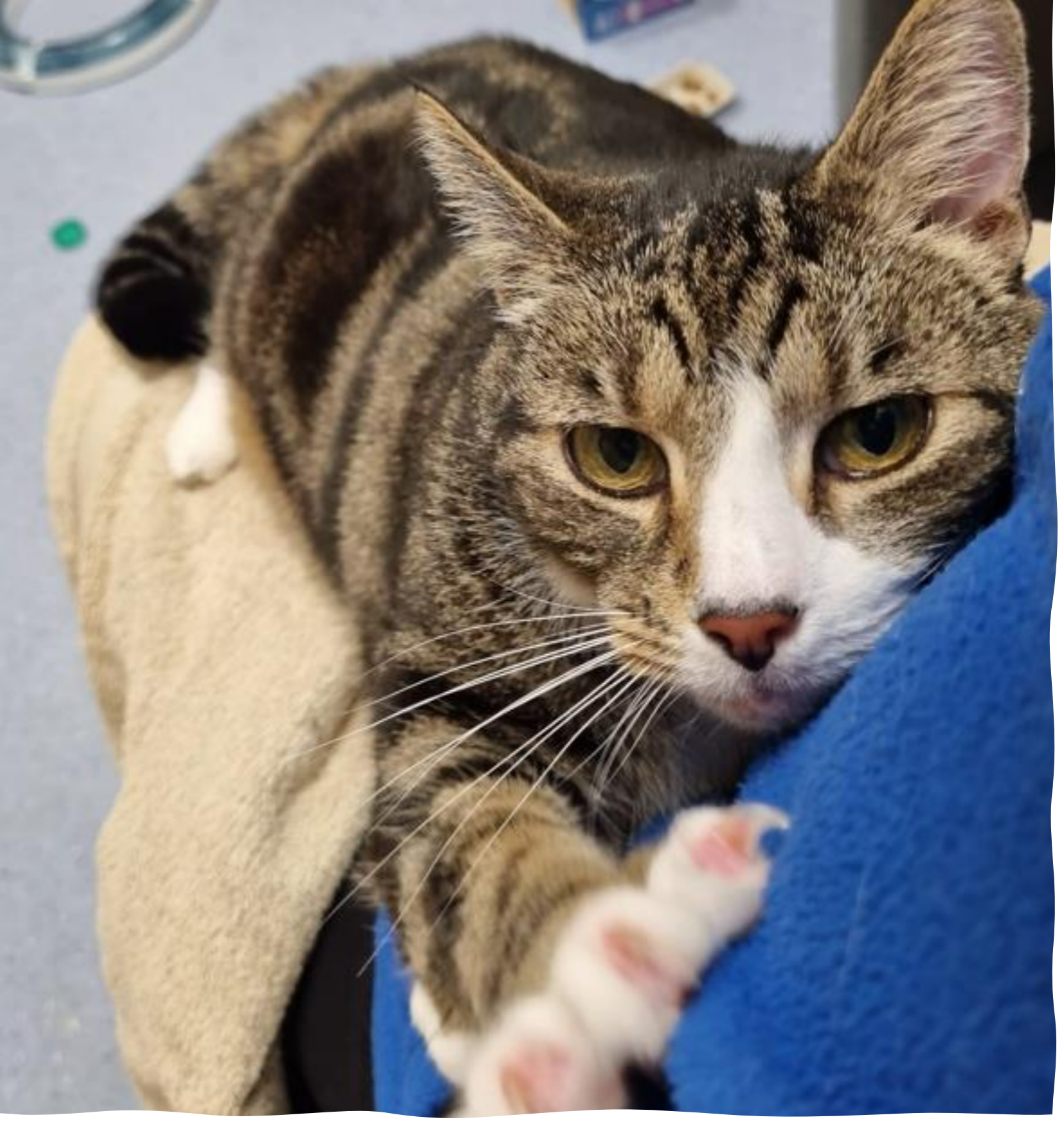

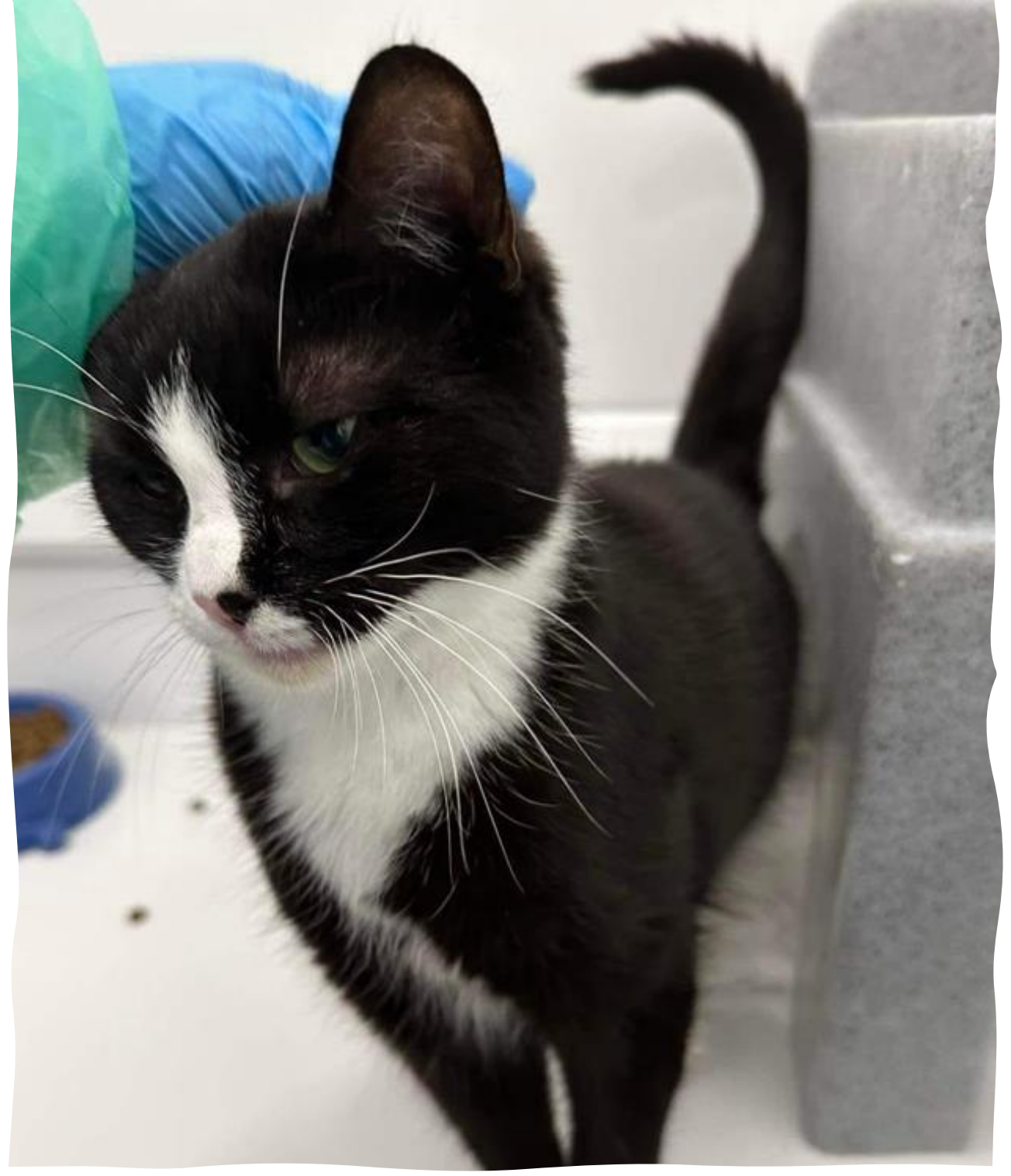

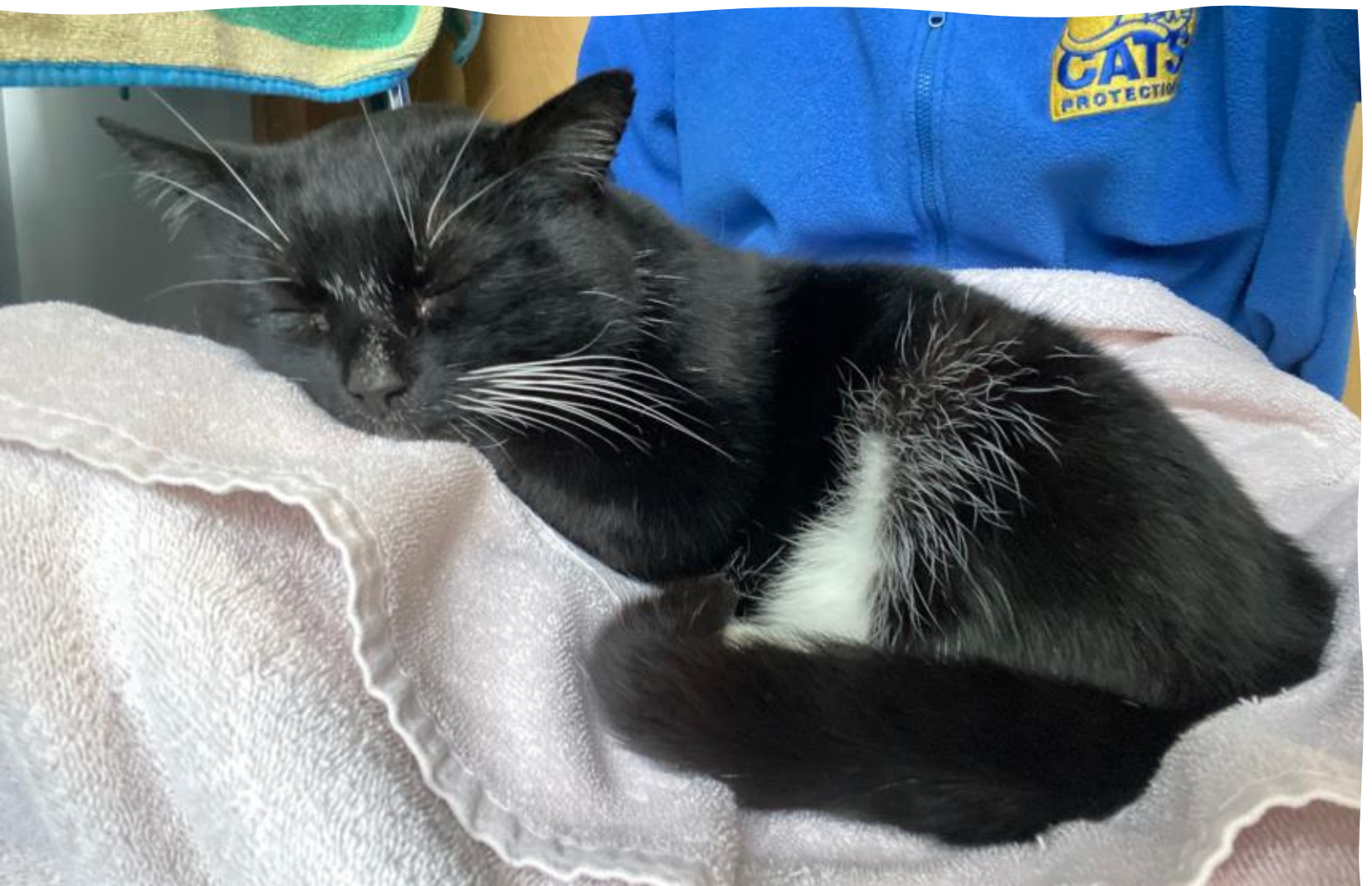

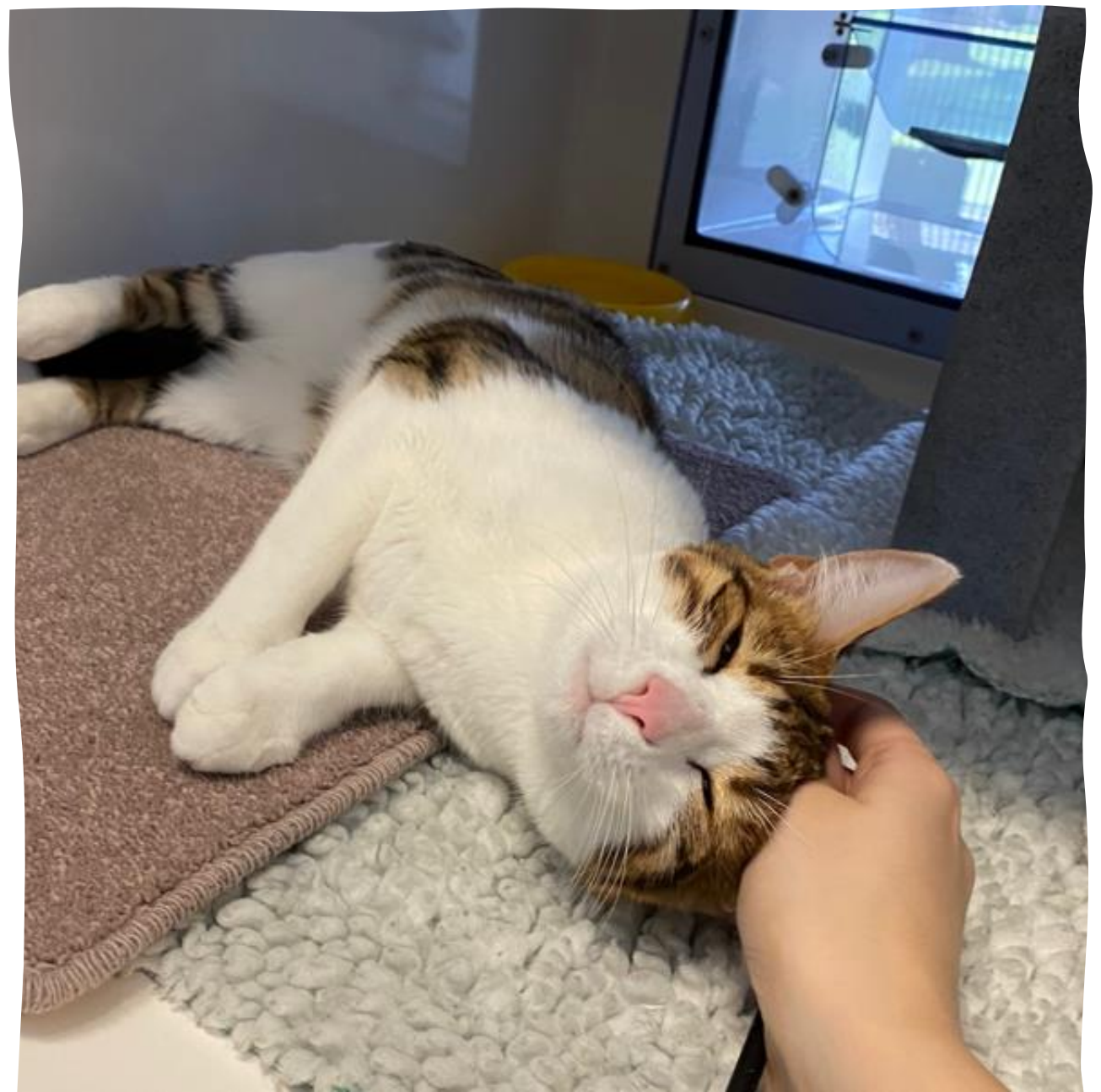

What signs may suggest a cat is enjoying the interaction?

- On person
- Presents area for touching
- Rubs person
- Kneading
- Gazes up at person
- Relaxed horizontal posture
- Ears upright and relaxed

Monitoring Interactions

- How do you monitor your interactions with cats?
- Why are written observations important?

PROTECTION

Cat name: Pen: Start weight: End weight:

Cat ID: Age: Sex: Colour:

Reason for observation:
(e.g. Admission, ill, under/overweight, food monitoring, water intake etc)

Diet: Wet / Dry (delete as appropriate) Comments:

Defecation Grade (DG): 1-Watery 2-Sloppy 3-Soft 4-Semi solid 5-Solid 6-Hard Dry
*=Tick/cross/? In box then add comments if needed

Date	Time	Eating*		Drinking	Urination		DG		Comment	Initial
		Wet	Dry		Inside	Outside	Inside	Outside		
	AM									
	Noon									
	PM									
	AM									
	Noon									
	PM									
	AM									
	Noon									
	PM									

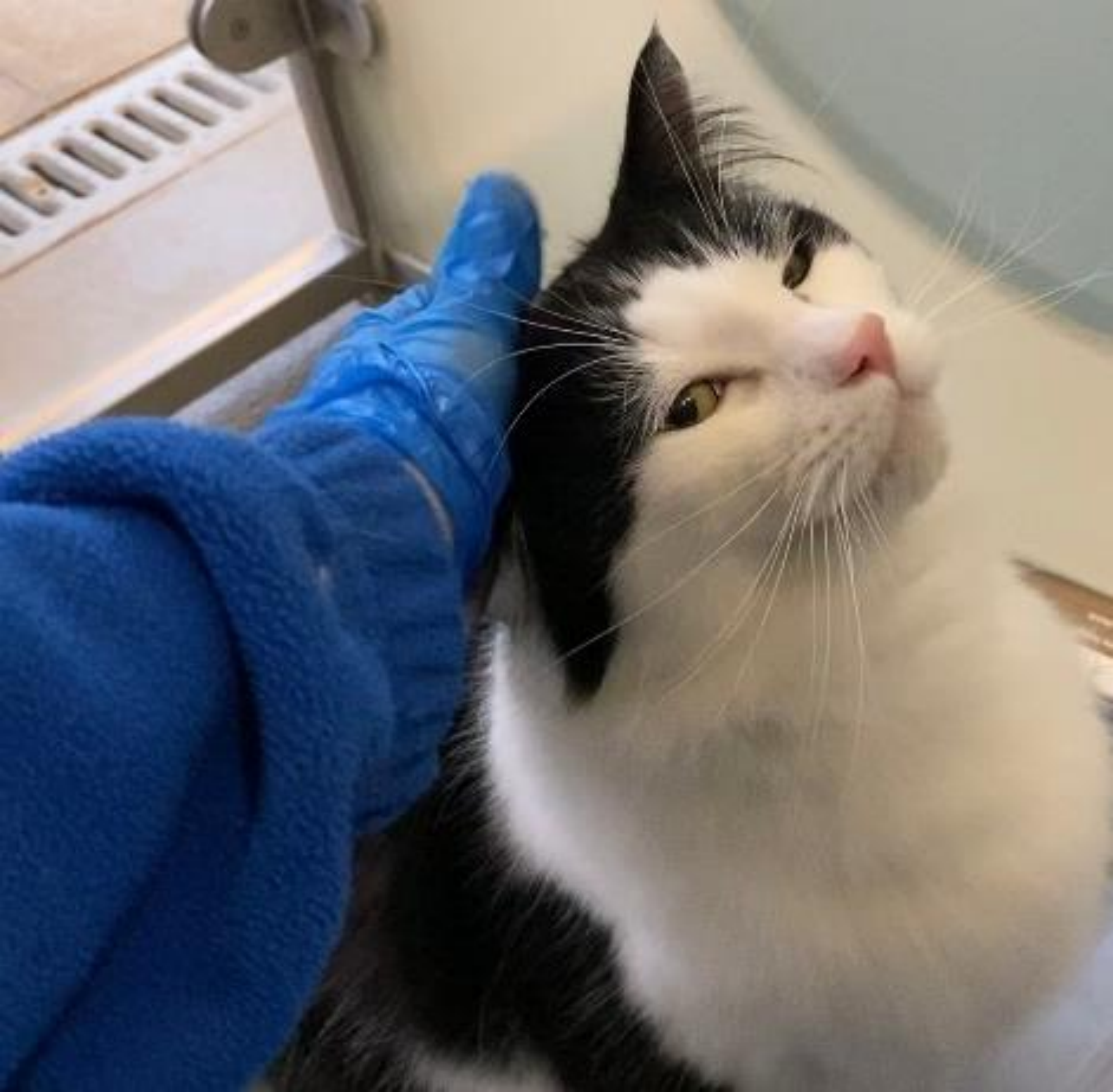

T – Where am I touching the cat and does the cat want me to keep touching?

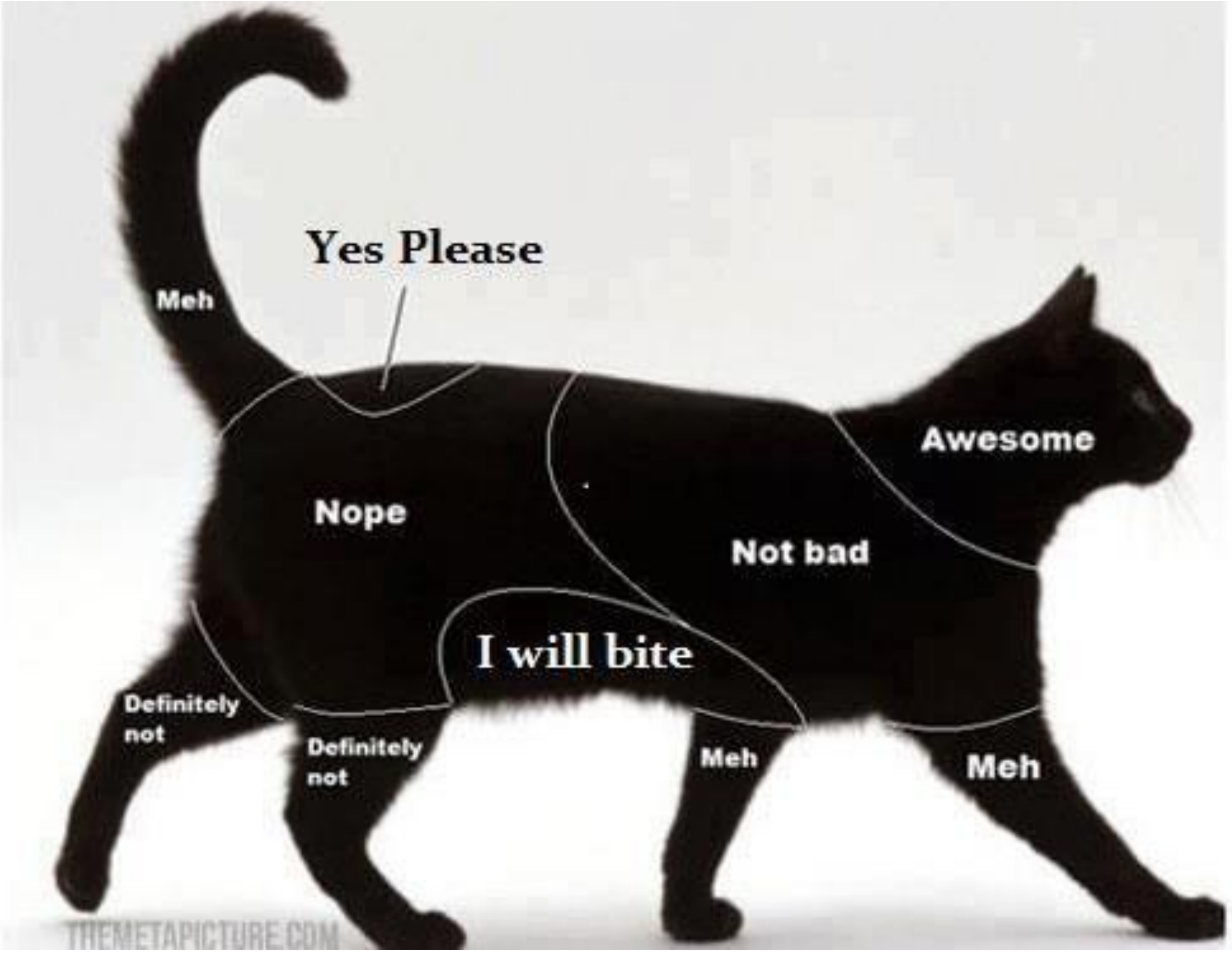

Top tip - **3 second** rule of touch!

Flooding

What is flooding?

- The exposure to something the cat finds scary or very distressing without opportunity to escape or hide.

- If this is combined with not allowing the cat to display its normal coping mechanisms, this can result in the cat shutting down = **learned helplessness.**

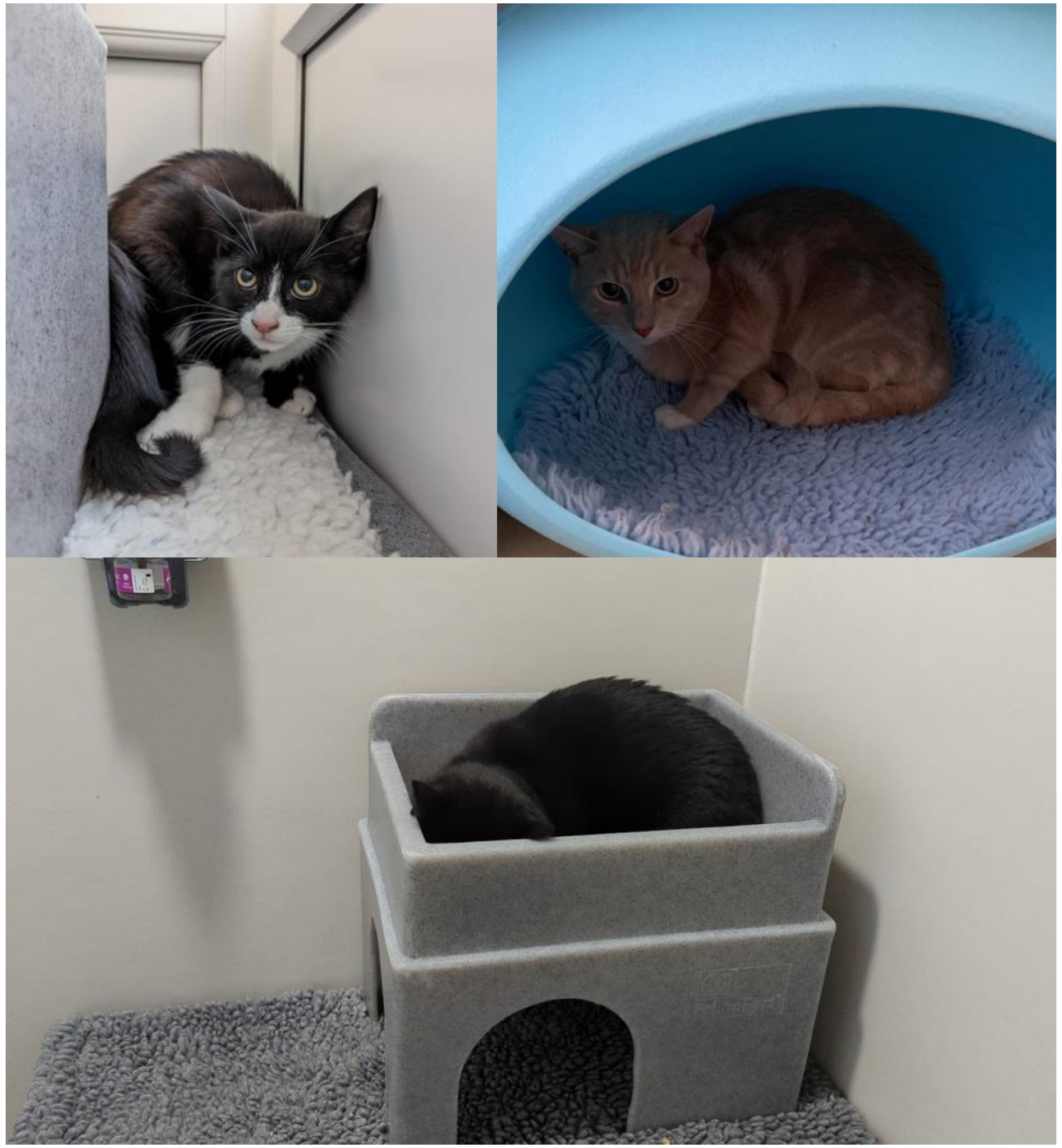

Human behaviour change

What will you take away from today?

Change:

- Needs to start with us!
- Our assumptions
- Our expectations
- Acceptance
- Putting the CAT in to practice
- Educating others

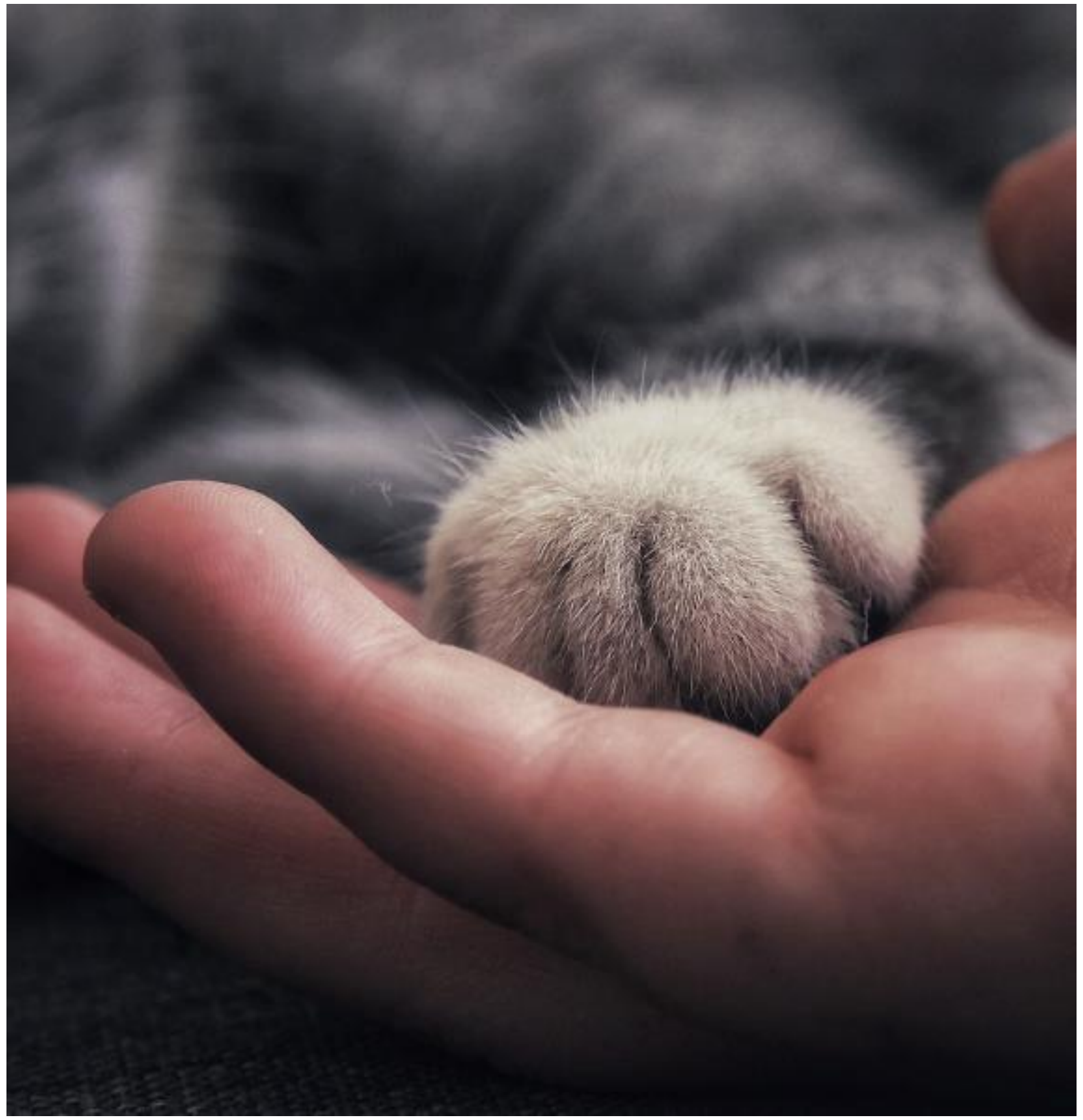

What did we look at today?

- Explored our relationship with cats and how that affects our interaction with cats
- The impact stress has on any interaction and why it is important to manage stress
- Identified the importance of positive interactions and approach with cats in our care
- Recognised how to adapt to a cat's body language

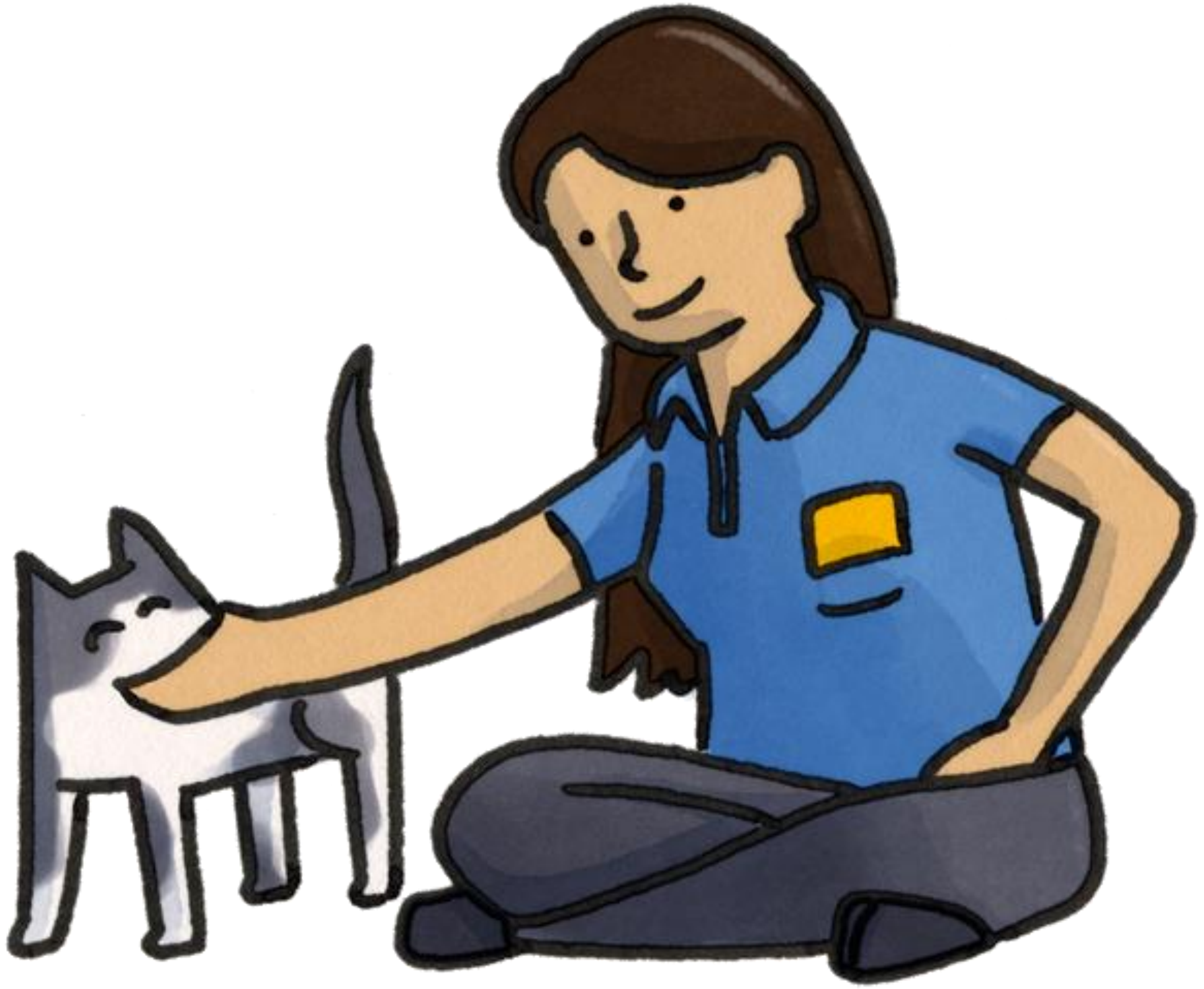

Support with Cat Welfare Learning enquiries

- For any Cat Welfare Learning related queries, please contact us directly on catwelfarelearning@cats.org.uk

- We look forward to welcoming you onto another Cat Welfare Learning workshop again soon!

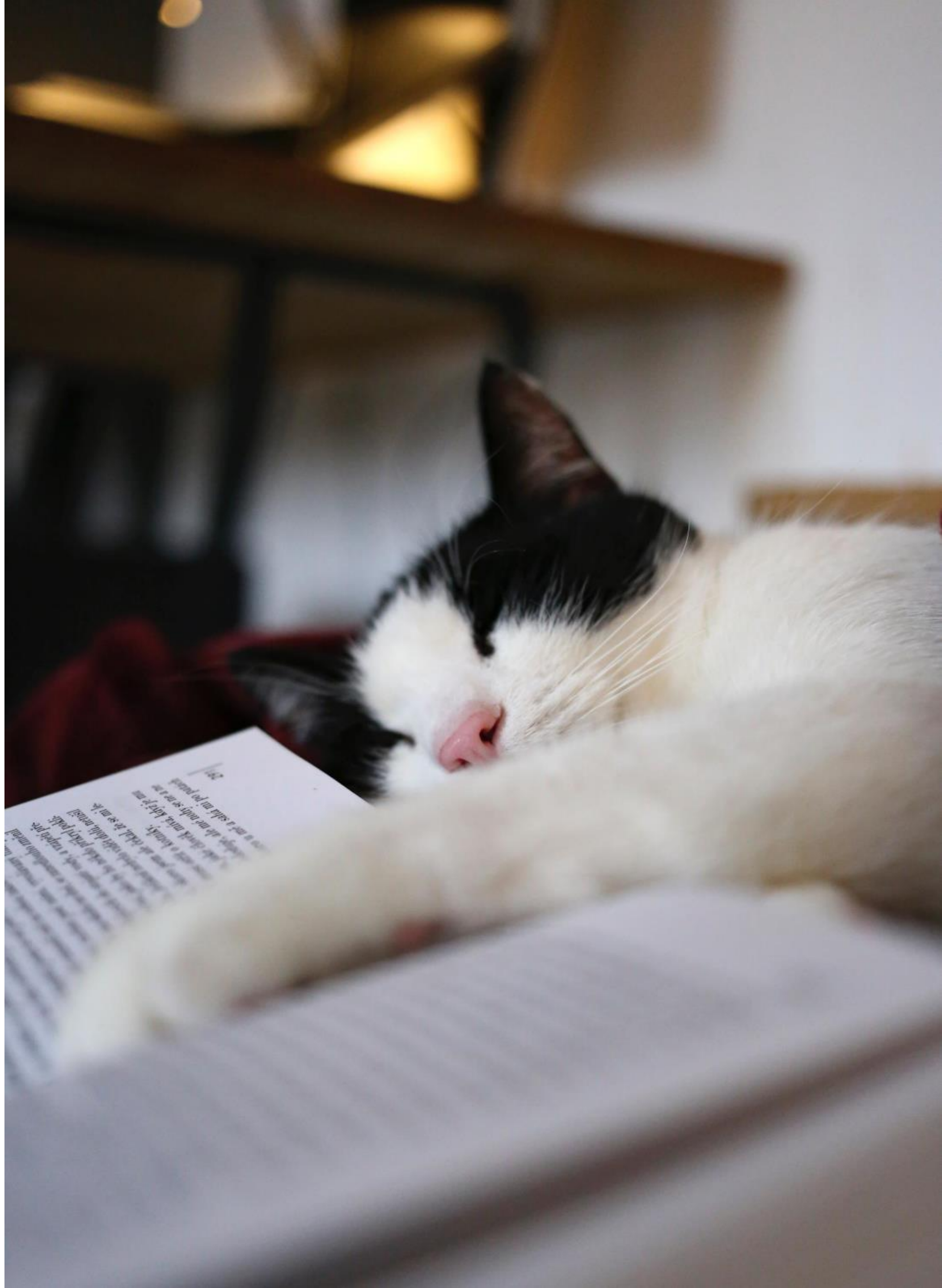

Support with cat welfare

- For support with cat welfare, please contact your Cat Welfare Advisor directly or email catwelfareteam@cats.org.uk
- The Cat Welfare team carry out annual welfare support visits to fosterers across Cats Protection branches and are also here to help with any cat welfare support need you may have
- Please do get in touch – we are happy to help!

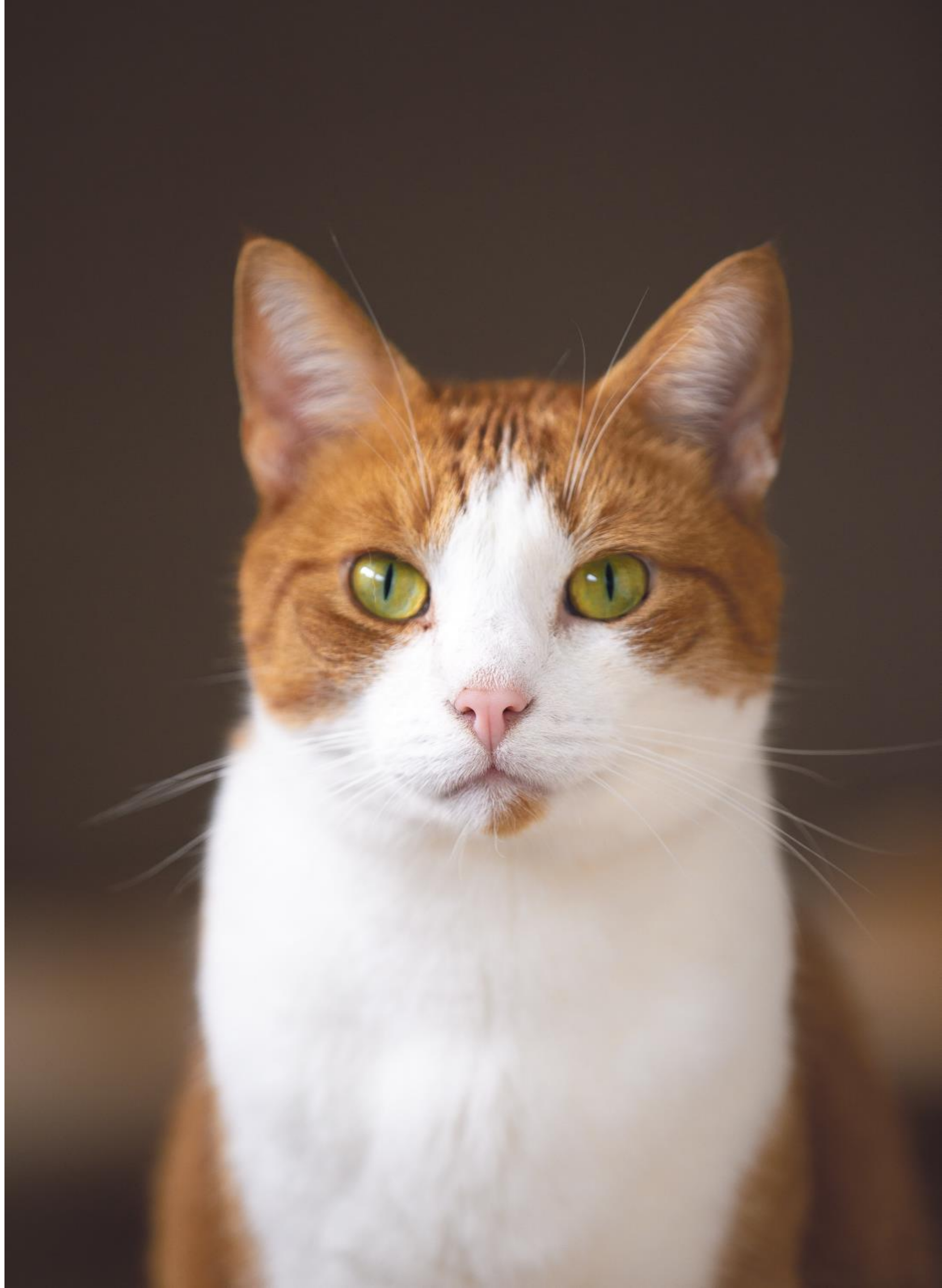

Support with veterinary queries

- For any branch-related veterinary queries, please contact the Feline Welfare Directorate - veterinary@cats.org.uk

- The team can help assist with ongoing/ complicated cases and is happy to offer case-specific input as needed

- Can also directly liaise with vets in practice to give vet to vet advice re. CP policies and guidance

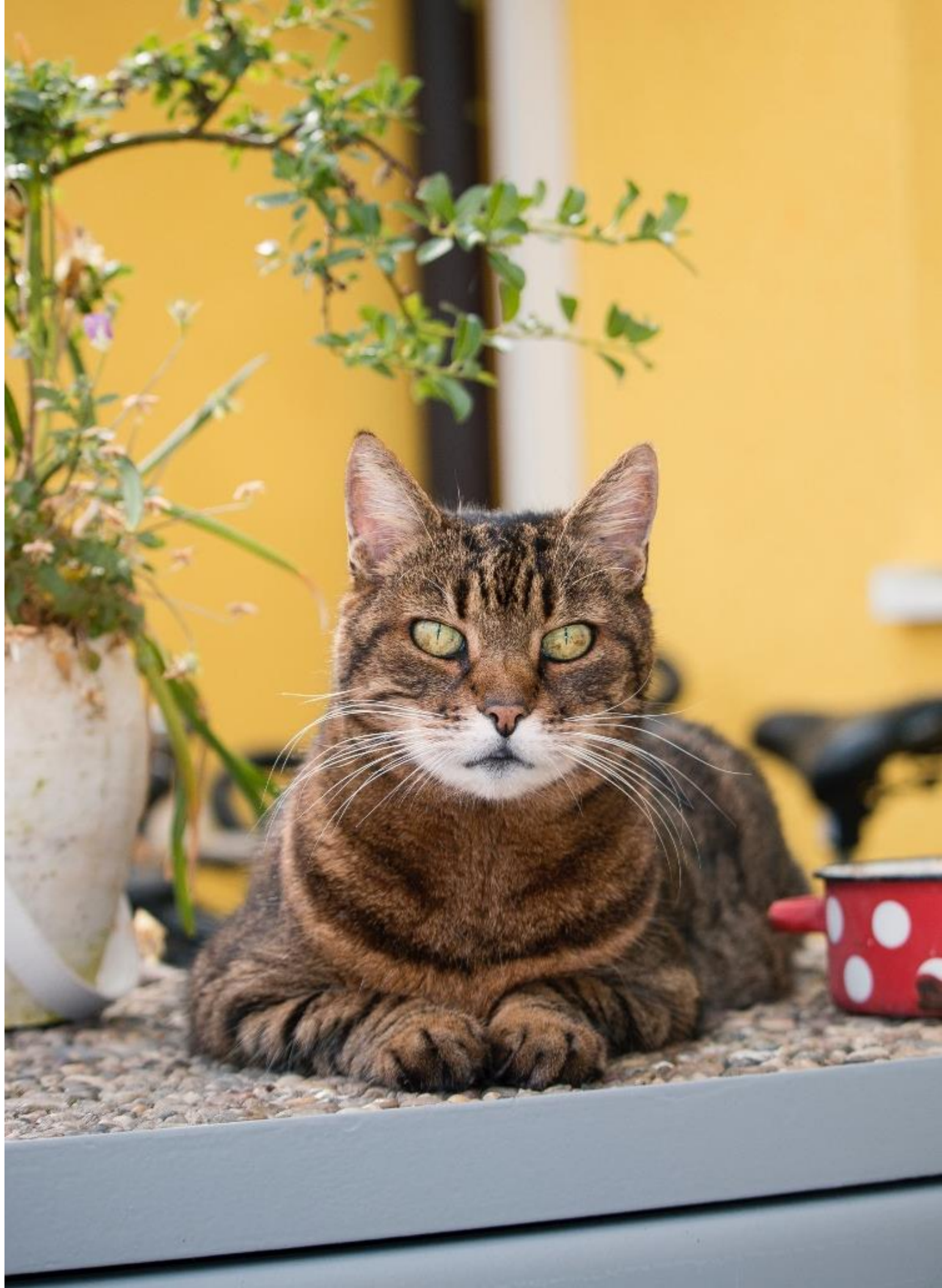

Support with feline behaviour

- For support with feline behaviour, contact the Behaviour team via behaviour@cats.org.uk

- Behaviour support available for all cats and caregivers within Cats Protection (branches and centres)

- Also available for cats rehomed up within the last 6 months

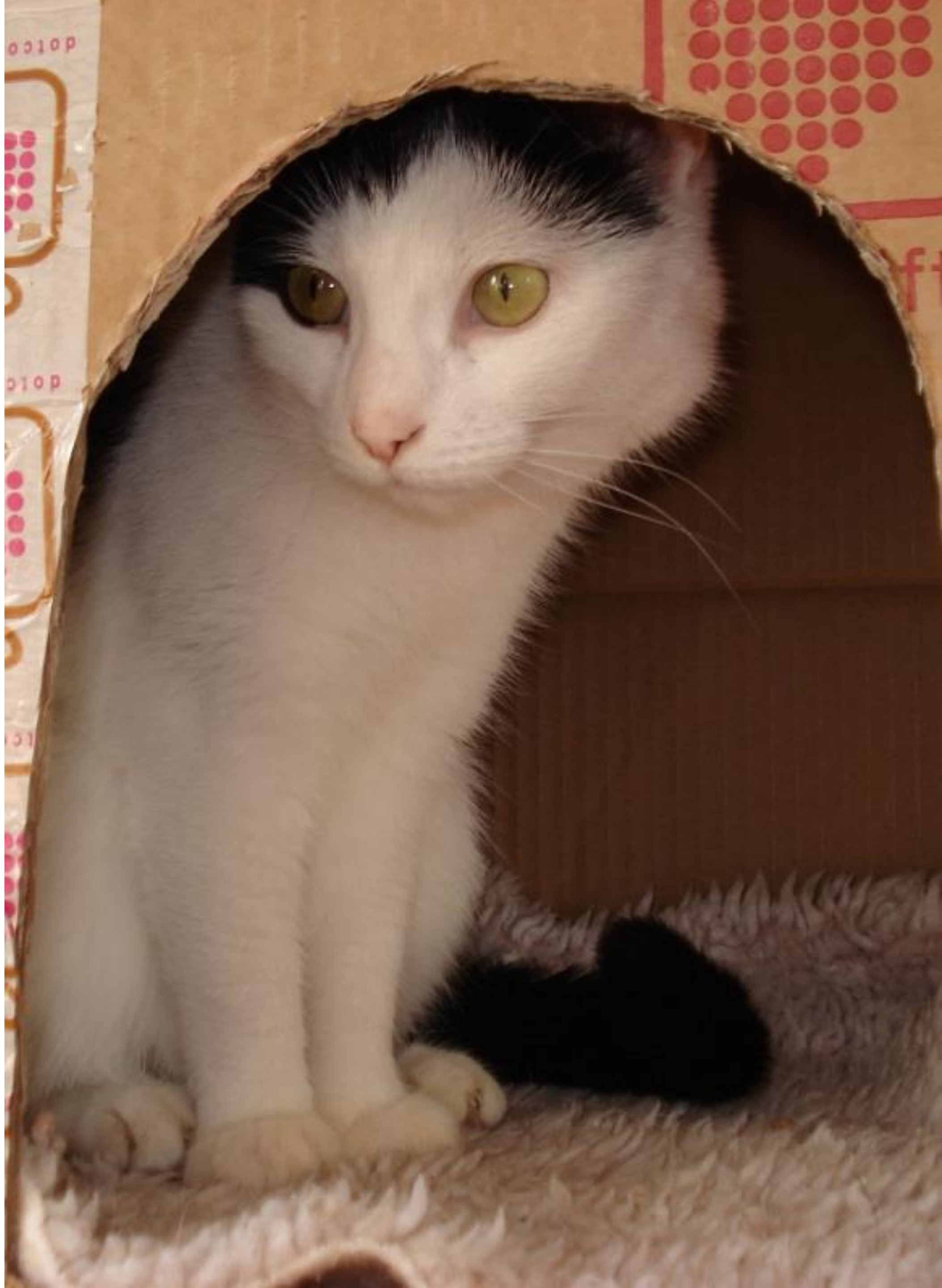

Supplement: Supplementary file 2 [file Data_Sheet_3.PDF]
