# Supplementary material for: Sustained improvements in human–cat interaction in the shelter sector: a study of a best-practice educational intervention
Source: Front Vet Sci. 2026 Aug 10;13:1880180. doi: 10.3389/fvets.2026.1880180 (PMC13500327; doi:10.3389/fvets.2026.1880180)
Supplement: Supplementary file 3 [file Data_Sheet_2.DOCX]

**Pre-Test Questionnaire**

***Section 1***

**1.Name:**

**2. Please indicate your age:**

( ) 18-25

( ) 26-35

( ) 36-45

( ) 46-55

( ) 56-65

( ) 66-75

( ) 76+

( ) Prefer not to say

**3. Please indicate your gender:**

( ) Female

( ) Male

( )Prefer not to say

( ) Prefer to use own term (Please indicate here):

**4. Please indicate whether you are a volunteer or employee:**

( ) Volunteer

( ) Employee

**5. Please indicate the length of time you have been a volunteer/employee at Cats Protection:**

( ) 0-12months

( ) 1-2 years

( ) 2-3 years

( ) 3-4 years

( ) 5-10 years

( ) 11- 15 years

( ) 16-19 years

( ) 20 years +

**6. Please indicate if you have attended the ‘Feline Interactions & Approach’ training session previously:**

( ) Yes (In person)

( ) Yes (Online)

( ) No

**If you answered yes, then please indicate how long ago you attended the session:**

( ) Under 6 months

( ) 6-12 months

( ) 12 months +

***Section 2: Attitudes***

**Please answer the following questions as honestly as possible:**

**1	2	3	4	5**

Strongly Disagree	Disagree	Neither agree	Agree	Strongly

or disagree	Agree

| Social interactions with humans are a core basic need of cats |  |  |  |  |  |
| --- | --- | --- | --- | --- | --- |
| Interactions with CP cats should always be equally beneficial for both the human and the cat |  |  |  |  |  |
| Cats relinquished from owners should always be homed as pet cats |  |  |  |  |  |
| If a CP cat appears fearful, unfriendly or behaves aggressively when stroked, it is important to keep up regular stroking sessions to increase their acceptance to handling |  |  |  |  |  |
| Cats coming in to shelters as strays should always be homed as pet cats |  |  |  |  |  |
| Each cat will have specific preferences for how they like to interact with humans |  |  |  |  |  |
| It is important to give cats a choice about whether they want to interact with humans or not |  |  |  |  |  |
| If a cat is anxious, shy or avoidant then it is important to ‘make the first move’ (e.g. by reaching out and touching them) during interactions to encourage them to respond |  |  |  |  |  |
| If a cat doesn't actively approach you and rub against you it means they would prefer not to be touched |  |  |  |  |  |
| It is hard to tell when cats are comfortable during interactions |  |  |  |  |  |
| I think that there are specific areas on the body that most cats do not like to be touched |  |  |  |  |  |
| It is hard to tell when cats are uncomfortable during interactions |  |  |  |  |  |
| It is most important to pay attention to the cat’s reactions when there is a risk of aggression |  |  |  |  |  |
| I am very confident about identifying signs that a cat is/isn’t enjoying interacting with me |  |  |  |  |  |
| The best way to tell if a cat has ‘had enough’ is when they bite, swipe, hiss or growl at you |  |  |  |  |  |

***Section 3: Practices***

**Please answer the following statements as honestly as possible:**

1. **When interacting with cats you don’t know/don’t know well, do you typically ….**

**1	2	3	4	5**

Never	Rarely	Sometimes Often	Always

| Remain a distance from the cat and wait to see if they approach you or not |  |  |  |  |  |
| --- | --- | --- | --- | --- | --- |
| Offer the cat your hand to sniff or rub against before actively touching them |  |  |  |  |  |
| If the cat behaves aggressively (i.e. hiss, growl, bite, swipe), briefly pause the interaction and then attempt to stroke them again |  |  |  |  |  |
| Avoid touching a cat that is hiding, attempting to hide or is resting in an elevated location such as a shelf |  |  |  |  |  |
| Stop stroking the cat at the point that they behave aggressively (i.e. hiss, growl, bite, swipe) |  |  |  |  |  |
| Take regular pauses when stroking the cat to ‘check in’, looking for signs that they want you to continue (e.g. leaning in towards your hand or rubbing against you) |  |  |  |  |  |
| Move towards the cat to carry on stroking them if they move or walk away from you |  |  |  |  |  |
| Pay close attention to the cat’s behaviour and body language during the interaction and adjust your interaction style accordingly |  |  |  |  |  |
| Stroke or touch the cat across a range of areas including their back, sides, tail |  |  |  |  |  |
| Allow the cat to move or walk away from you during the interaction |  |  |  |  |  |
| Stroke or touch the cat mostly around their facial areas (i.e. chin, cheeks and base of their ears) |  |  |  |  |  |
| Pick the cat up to hold them or place on your lap |  |  |  |  |  |
| Avoid or stop touching a cat that appears stressed, anxious or generally uncomfortable fearful |  |  |  |  |  |
| Stroke or touch the cat mostly on the areas of their body that they are actively choosing to rub against you/touch you with |  |  |  |  |  |
| Stroke or touch the cat’s legs, base of tail or tummy |  |  |  |  |  |
| Avoid or stop touching the cat if you see any of the following behaviours:  • The cat turns it head or moves its body away from you  • Their ears become flattened or rotate backwards  • They shake their head and/or body  • The fur on their back appears to ripple  • They lick their nose  • They go a bit still or appear to ‘freeze’  • They sharply turn their head to face you or your hand  • They suddenly start grooming themselves, lasting only a few seconds  • Their tail twitches or ‘swishes' vigorously, usually when held horizontally or close to the ground , They have a tense, upright but crouched posture, They appear a bit jumpy or on high alert |  |  |  |  |  |
| Avoid or stop touching the cat if they are not displaying behaviours such as purring, dribbling rubbing against you or kneading with their paws |  |  |  |  |  |
| In situations where you are worried about a cat behaving aggressively, use a fake hand or other inanimate object to touch the cat with |  |  |  |  |  |
| Avoid touching the cat entirely if they make no attempt to approach you or initiate physical interaction |  |  |  |  |  |
| Make the first move by actively approaching the cat to touch them |  |  |  |  |  |
| Avoid disturbing or touching a cat that is sleeping, or in the middle of eating or grooming themselves |  |  |  |  |  |
| End the interaction early if the cat makes no attempt to approach you or initiate physical interaction |  |  |  |  |  |

2. **Have you heard about the C.A.T handling guidelines before?**

( ) Yes (please briefly explain what you know about them and where you heard about them)

( ) No

If you answered yes to the above question, briefly explain what you know about them and where you heard about them
