# Supplementary material for: Sustained improvements in human–cat interaction in the shelter sector: a study of a best-practice educational intervention
Source: Front Vet Sci. 2026 Aug 10;13:1880180. doi: 10.3389/fvets.2026.1880180 (PMC13500327; doi:10.3389/fvets.2026.1880180)
Supplement: Supplementary file 4 [file Data_Sheet_4.DOCX]

**Participant Information**

**Sheet**

My name is Elle Boden, Regional Behaviour Officer at Cats Protection, and this research forms part of my Masters at Glyndŵr University. This is your chance to be involved in a new research project at Cats Protection focused on Human-Cat interactions. The results of this study will help us to better understand how we optimise human-cat relationships in different settings.

Before you agree to participate, it is important that you understand the purpose and nature of the research and what your participation will involve, if you agree. Please read the following information carefully and feel free to ask us if you would like more information or if there is anything you do not understand. Contact details are given at the end of this information sheet.

**Do I have to take part?**

Participation is entirely voluntary. It is up to you to decide whether to take part. If you do agree to take part, you will be asked to sign a consent form. If you agree to take part, you may still withdraw at any time, without giving a reason. If that happens, please note that you will not be able to withdraw your data after it has been anonymised on 20^th^ June 2024.

**What will taking part involve?**

1. Prior to the training session we will ask you to record a 90 second video of you interacting with a cat (this can be either your own cat, a foster cat or adoption centre cat but must not be a Lifeline cat). All videos will need to be sent to us via the messaging service ‘WhatsApp’ using the telephone number 07824 115147

2. After we have received your video, we will then send you an email with a very short questionnaire for you to complete and return.

3. Attend the ‘Feline Interactions and Approach’ session.

4. Once you have attended the training session, we would like you to record a second 90 second video of you interacting with a cat (this again can be either your own cat, a foster cat or adoption centre cat but must not be a Lifeline cat). All videos will need to be sent to using the same number as above via ‘WhatsApp’.

5. Lastly, we will then email you with a second very short questionnaire for you to complete and return.

**What are the potential risks and benefits to taking part?**

The results of this study will help us to better understand how we optimise human-cat relationships in different settings. However, interacting with any cat poses a risk to both the cat and human, therefore we have put together a brief hand out ‘Recording your video’ outlining how to mitigate these risks and ensure the welfare of the cat is always upheld.

**Will my participation be confidential?**

All information about you collected during the study will be kept strictly confidential and stored securely in accordance with the Data Protection Act. The only people who will have access to the data are the researcher and Cats Protection research team. All data will be anonymised after the 20^th^ June 2024, the questionnaires and video footage will then be destroyed by being permanently deletion from all devices. Anonymised data will then be held for 6 years on the Cats Protection secure VPN with access only by the internal research team.

**What will happen to the results of the study?**

The results of this study will be available once graded by Wrexham university, details will be circulated internally within Cats Protection. The study may also be published externally and access to this will again be communicated via the internal comms team at Cats Protection. Participants will not be identifiable from the results.

**What will happen if I want to stop taking part?**

You can withdraw participation at any time without disadvantage or explanation. If you change your mind, you can contact the researcher up to 20^th^ June 2024 before the data has become fully anonymised.

**What if I am unhappy or if there is a problem?**

If you are unhappy, or if there is a problem, please feel free to let us know by contacting Dr Tamsin Young ([t.young@glyndwr.ac.uk](mailto:t.young@glyndwr.ac.uk)) and we will try to help. If you remain unhappy or have a complaint which you feel you cannot come to us with then you should contact the Research Ethics Sub- Committee at [rescadmin@glyndwr.ac.uk](mailto:rescadmin@glyndwr.ac.uk). When contacting the Research Ethics Sub-Committee please provide details of the name or description of the study (so that it can be identified), the researcher(s) involved, and the details of the complaint you wish to make.

**Contact for further information**

*If anything is not clear, or if you want more information, please do contact me:*

Elle Boden, Regional Behaviour Officer

Email: elle.boden@cats.org.uk

# PRIVACY NOTICE INFORMATION SHEET FOR PARTICIPANTS IN RESEARCH PROJECTS

| **Introduction** | |
| --- | --- |
| Please note that this standard information should be considered alongside information provided by the researcher for each project, which is usually in the form of a Participant Information Sheet (PIS). The PIS will include further details about how personal information is processed in the particular project, including: what data is being processed; how it is being stored; how long it will be retained for, and any other recipients of the personal information. It is usually given to participants before they decide whether or not they want to participate in the research. | |
| **Data Controller and the Data Protection Officer** | |
| Glyndŵr University is the data controller under data protection legislation. This means that the University is responsible for how your personal data is used and for responding to any requests from you in relation to your personal data. Any queries regarding your personal information should be made to the University’s Data Protection Officer at [dpo@glyndwr.ac.uk](mailto:dpo@glyndwr.ac.uk) | |
| **Legal Basis for processing your personal information** | |
| If you are participating in a research project, we may collect your personal information. The type of information that we collect will vary depending on the project. Our basis for collecting this information is outlined below: | |
| **Type of Information** | **Basis for Processing** |
| Your personal information and associated research data collected for the purposes of conducting research | It is necessary for the performance of a task carried out in the public interest |
| Certain types of personal information such as information about your race, ethnic origin, politics, religion, trade union membership, genetics, biometric (where used for ID purposes) health sex life, or sexual orientation are defined as @special Category’ data under the legislation | It is necessary for the performance of a task carried out in the public interest, and  It is necessary for scientific or historical research purposes in accordance with the relevant legislation (Data Protection Act 2018. Schedule 1, Part 1 Para 4) |
| Criminal conviction/offence data | It is necessary for the performance of a task carried out in the public interest, and  Is processed in accordance with Article 10 of the General Data Protection Regulation and the Data Protection Act 2018, Schedule 1 Part 1 Para 4 |
| **Retention of consent forms** | |
| If you participate in a research project, you may be asked to sign a participant consent form. Consent forms will typically be retained by the University for at least as long as the identifiable research data are retained, In most cases they will be retained for longer, the exact time frame will be determined by the need for access to this information in the unfortunate case of an unanticipated problem or a complaint six (6) years after the research is completed for many projects, but beyond 20 years will be considered for any longitudinal or ‘high risk’ studies involving children, adults without capacity or a contentious research outcome. | |
| **Your Rights** | |
| You have the right to: be informed about the collection and use of your personal data; to request access to the personal data we hold about you; you are entitled to request to have personal data rectified if it is inaccurate or incomplete; you have the right to request to object to your data being processed and you can request to restrict the processing of your personal information. To exercise these rights please contact [dpo@glyndwr.ac.uk](mailto:dpo@glyndwr.ac.uk) | |
| **Right to complain to supervisory authority** | |
| If you have any concerns/issues with the way the University has processed your personal data, you can contact the data protection officer at [dpo@glyndwr.ac.uk](mailto:dpo@glyndwr.ac.uk). You also have the right to lodge a complaint against the University regarding data protection issues with the Information Commissioners Office (<https://ico.org.uk/concerns/>) | |

# **Consent Form**

**Version number & date:** 1, March 2024

Researcher: Elle Boden, Regional Behaviour Officer (elle.boden@cats.org.uk)

Please read each statement and sign below.

| I confirm that I have read and understood the Information Sheet dated March 2024 for the above study, or it has been read to me. If I have asked for clarification or for more information, I have had satisfactory responses. |  |
| --- | --- |
| I understand that taking part in the study involves a pre and post training session questionnaire and a video recording of me interacting with a cat (no lifeline cats should be used). |  |
| I understand and agree that my participation will involve recording myself on video*.* I am aware of and consent to your use of these recordings for the following purposes*: Solely for the use of research purposes, only the research team will be able to view these (videos & questionnaires containing personal data will be destroyed by permanently deleting them from 20^th^ June 2024).* |  |
| I understand that I can ask for access to the information I provide, and I can request the destruction of that information if I wish at any time prior to 20^th^ June 2024*.* I understand that following 20^th^ June 2024 I will no longer be able to request access to or withdrawal of the information I provide, as the data will have been anonymised. |  |
| I understand that the information I provide will be held securely and in line with data protection requirements reflected on the Glyndwr University Privacy Notice until it is fully anonymised and then deposited in the Cats Protection research archive for sharing and use by other authorised Cats Protection researchers to support other research in the future. |  |
| I agree to take part in the above study |  |

Participant name	Date	Signature
