# Supplementary material for: Sustained improvements in human–cat interaction in the shelter sector: a study of a best-practice educational intervention
Source: Front Vet Sci. 2026 Aug 10;13:1880180. doi: 10.3389/fvets.2026.1880180 (PMC13500327; doi:10.3389/fvets.2026.1880180)
Supplement: Supplementary file 5 [file Data_Sheet_5.DOCX]

**Recording your video**

**Below you will find guidance on how to record your video of you interacting with a cat.**

- **Start by propping up your phone or having someone record you whilst you video the interactions. We do not recommend trying to hold the phone yourself whilst recording as we need to be able to see both you and the cat throughout.**
- **Try to always stay seated and in the same place whilst recording your video. (Unless this is an adoption centre cat where sitting is not cannot be performed. In this case you should follow your usual protocol)**
- **Ensure you record the video in an area that the cat is already familiar with. If this is a Cats Protection cat, we would recommend their pen. If this is your own cat, then ideally choose a room in the house that they are comfortable with.**
- **We’d like you to interact with the cat in the way that you would normally interact with them, we simply want to see you naturally interact with cats.**
- **Please ensure the video is at least 90 seconds.**
- **Once you have recorded your video please send it to 07824 115147 via the messaging service ‘WhatsApp’, please state your full name and whether this is your own cat or a CP cat.**
- **We will then ask you to do a second video recording after your training session, ideally this will be the same cat as the first video. This should be sent within 2 weeks of the training session.**
